# Supplementary material for: Photocatalytic C(sp3)–C(sp3) cross-coupling of carboxylic acids and alkyl halides using a nickel complex and carbon nitride
Source: Nat Commun. 2025 Jul 31;16:7016. doi: 10.1038/s41467-025-61639-8 (PMC12313896; doi:10.1038/s41467-025-61639-8)
Supplement: Supplementary file 1 — Supplementary Information [file 41467_2025_61639_MOESM1_ESM.pdf]

Table of Content.

General information. page S3

Optimization studies. page S4

Synthesis and characterization of other catalytic materials. page S8

Recyclability tests and post-catalysis characterization. page S10

Mechanistic studies. page S13

- Stern-Volmer experiments. page S13
- Radical trap experiments. page S14
- EPR experiments. page S16
- DFT calculations. . page S17

Life cycle assessment and analysis of the GHG emission potential. page S25

Experimental data. page S25

NMR spectra. page S40

Supplementary references. page S67

General information.

Unless otherwise noted, the reagents and solvents were purchased from commercial suppliers and used without further purification. Also, unless specified, the reactions were performed without inert gas or anhydrous solvent, using a commercial Solstice™ parallel 12-Position LED Photoreactor (Uniqsis Ltd.), equipped with a Borealis™ LED light source (Uniqsis Ltd.) at 420 nm and 120 W (Figure S1a). Thin layer chromatography (TLC) was performed on pre-coated silica gel 60 F254 plates (Merck) and visualized using UV-light. Organic solutions were concentrated under reduced pressure on a Büchi rotary evaporator using a water bath. The crude products were analyzed *via* high-performance liquid chromatography (HPLC). The conversion of the limiting reagent was deduced using an Agilent 1200 instrument, with an ultraviolet-visible detector (G1315D) set at 210 nm, injecting the samples and the standard (5 µL) directly onto a 250 mm × 4.6 mm Hypersil GOLD™ aQ 5 µm 175 Å column (Thermo Scientific). NMR measurements of the isolated products were carried out using a 400 MHz Bruker AVANCE II with a BBO probe at 298 K. Chemical shifts are given in ppm relative to the residual solvent peak (^{1}H-NMR δ: $CDCl_3$ = 7.26. ^{13}C-NMR δ: $CDCl_3$ = 77.16) and all coupling constants (*J*) are given in Hertz (Hz) with their multiplicity: (s = singlet, d = doublet, t = triplet, q = quartet, p = quintet, m = multiplet, dd = doublet of doublets, dt = doublet of triplets, br = broad). X-band CW-EPR spectra were recorded on a Bruker EMX spectrometer (MW frequency 9.45 GHz) equipped with a cylindrical cavity. All the spectra were recorded at 77 K (a finger dewar was inserted inside the EPR cavity and filled with liquid nitrogen) with a modulation frequency of 100 kHz, a modulation amplitude of 0.5 mT and a microwave power of 1.047 mW (23 dB). A High Power SOLIS-LED from THORLABS with dominant wavelength at 415nm and 28W was used as source of light to perform the experiment.

Optimization studies.

The crude reaction products were analyzed using high-performance liquid chromatography (HPLC). Upon completion of the reaction, a 100 μL aliquot was taken and diluted with acetonitrile in a 2 mL HPLC vial. The conversion of product **3a** was determined using an Agilent 1200 instrument equipped with a UV-Vis detector (G1315D) set to 210 nm. Samples and standards (5 μL each) were injected directly onto a 250 mm × 4.6 mm Hypersil GOLD™ aQ column (Thermo Scientific) with 5 μm particle size and 175 Å pore size. A calibration curve was constructed (Figure S1b) by measuring the area under the curve (AUC) for different **3a** solutions corresponding to yields of 100% (0.1 M), 75% (0.075 M), 50% (0.05 M), 25% (0.025 M), and 12.5% (0.0125 M).

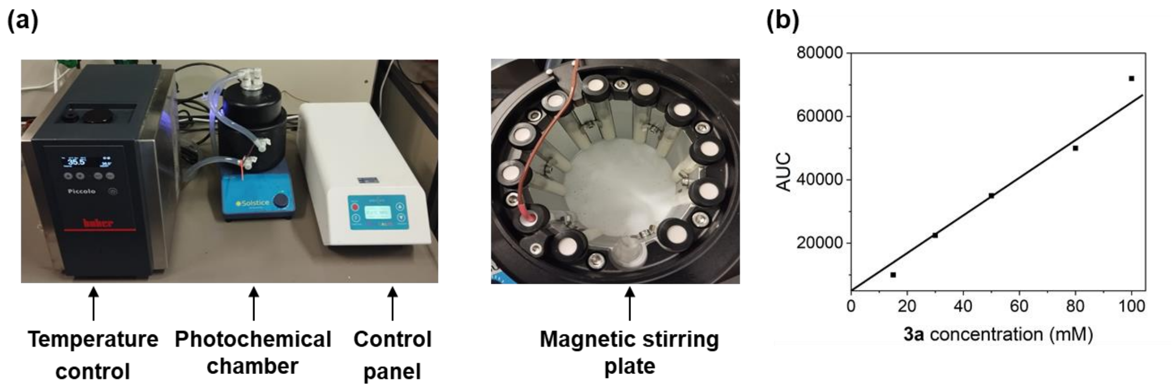

Figure S1. Optimization studies set-up. *Uniqsis photoreactor used for the photocatalytic experiments (**a**). High-performance liquid chromatography calibration curve for the model product **3a** (**b**).*

Table S1. *Optimization of the base.*

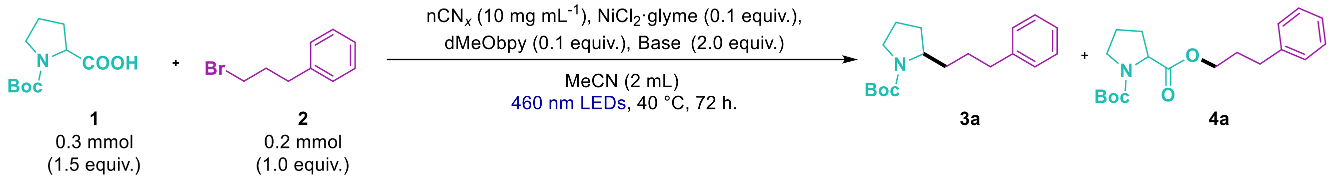

Entry	Base	Yield of **3a**[a]	Yield of **4a**[a]
1	Cs_2CO_3	8%	92%
2	K_2CO_3	34%	44%
3	Na_2CO_3	33%	17%
4	$NaHCO_3$	n.d.[b]	6%
5	Li_2CO_3	n.d.	n.d.
6	K_2HPO_4	28%	40%
7	NaOH	n.d.	15%
8	Triethylamine	6%	25%
9	DBU	n.d.	99%
10	Quinuclidine	n.d.	96%
11	*N*-*tert*-Butylisopropylamine	14%	19%
12	DIPEA	5%	29%
13	Dibutylamine	17%	20%
14	Disiopropylamine	23%	10%
15	2,6-Lutidine	n.d.	n.d.

[a]Yields were calculated by HPLC using a calibration curve. [b]Not detected.

Table S2. *Optimization of the base equivalents.*

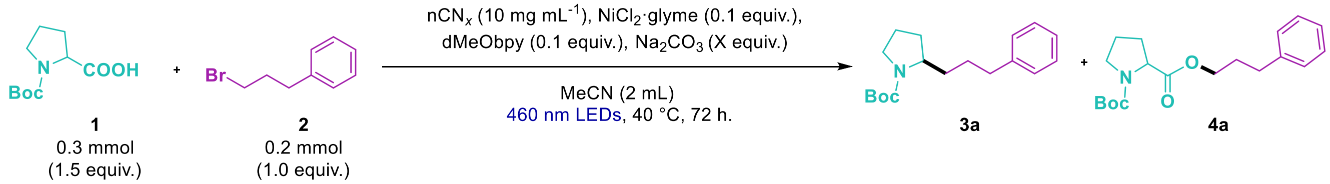

Entry	Na_2CO_3 (equiv.)	Yield of **3a**[a]	Yield of **4a**[a]
1	1.0	27%	24%
2	2.0	33%	17%
3	3.0	27%	20%
4	4.0	30%	19%

[a]Yields were calculated by HPLC using a calibration curve.

Table S3. *Optimization of the photocatalyst type.*

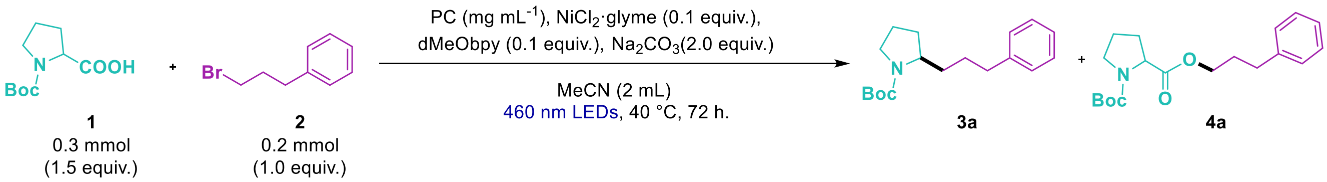

Entry	Photocatalyst (PC)	Yield of **3a**[a]	Yield of **4a**[a]
1	nCN$_x$ (10 mg mL^{-1})	33%	17%
2	nCN$_x$ (12.5 mg mL^{-1})	41%	23%
3	nCN$_x$ (15 mg mL^{-1})	17%	15%
4	mpgCN$_x$ (5 mg mL^{-1})	20%	12%
5	mpgCN$_x$ (7.5 mg mL^{-1})	34%	18%
6	mpgCN$_x$ (10 mg mL^{-1})	11%	23%
7	K-PHI (10 mg mL^{-1})	28%	43%

[a]*Yields were calculated by HPLC using a calibration curve.* [b]*Not detected.*

Table S4. *Optimization of the wavelenght.*

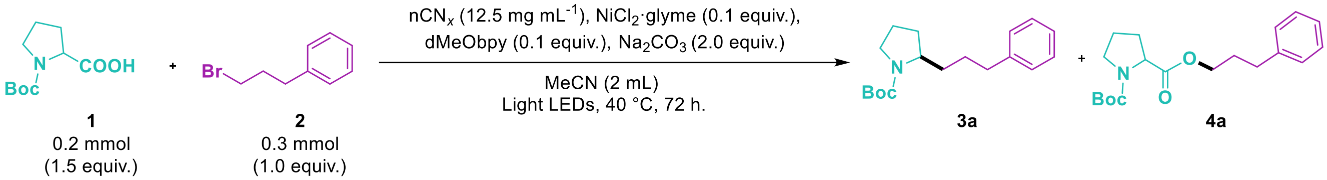

Entry	Light source	Yield of **3a**[a]	Yield of **4a**[a]
1	White LEDs	6%	51%
2	Green LEDs (500 nm)	n.d.[b]	11%
3	Blue LEDs (460 nm)	41%	23%
4	Purple LEDs (420 nm)	54%	20%

[a]*Yields were calculated by HPLC using a calibration curve.* [b]*Not detected.*

Table S5. *Optimization of the carboxylic acid equivalents.*

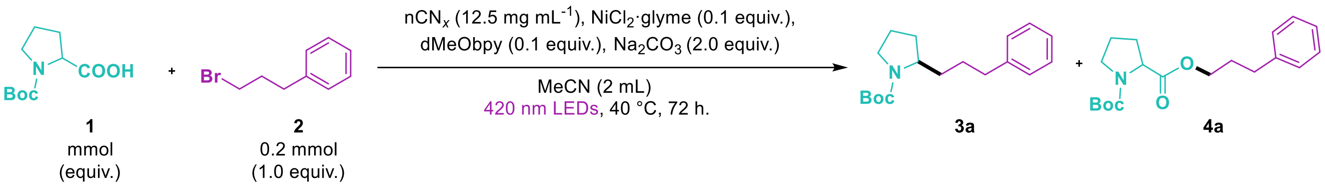

Entry	Carboxylic acid (equiv.)	Yield of **3a**[a]	Yield of **4a**[a]
1	1.0	31%	17%
2	1.5	54%	20%
3	3.0	39%	18%
4	5.0	18%	16%

[a]*Yields were calculated by HPLC using a calibration curve*.

Table S6. *Optimization of the nickel source.*

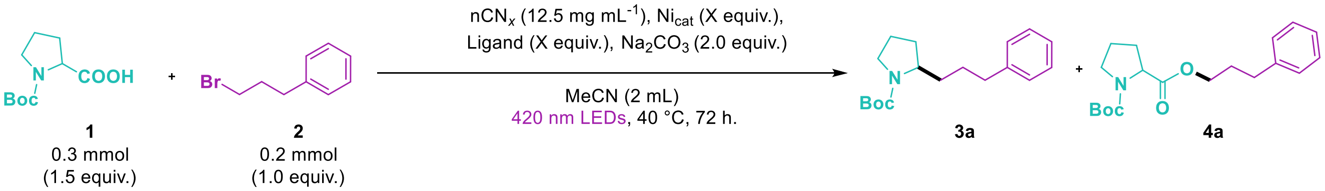

Entry	Ni_{cat} – ligand (equiv.)	Yield of **3a**[a]	Yield of **4a**[a]
1	$NiCl_2$ ·glyme – dMeObpy (0.1 equiv.)	54%	20%
2	$NiBr_2$ ·glyme – dMeObpy (0.1 equiv.)	14%	10%
3	$NiCl_2$ – dMeObpy (0.1 equiv.)	17%	14%
4	$Ni(Ac)_2$ ·4 H_2O – dMeObpy (0.1 equiv.)	41%	10%
5	NiO – dMeObpy (0.1 equiv.)	n.d.[b]	11%
6	$NiCl_2$ ·glyme – dtbbpy (0.1 equiv.)	45%	17%
7	$NiCl_2$ ·glyme – dMeObpy (0.2 equiv.)	60%	10%

[a]*Yields were calculated by HPLC using a calibration curve.* [b]*Not detected.*

Table S7. *Optimization of the reaction concentration.*

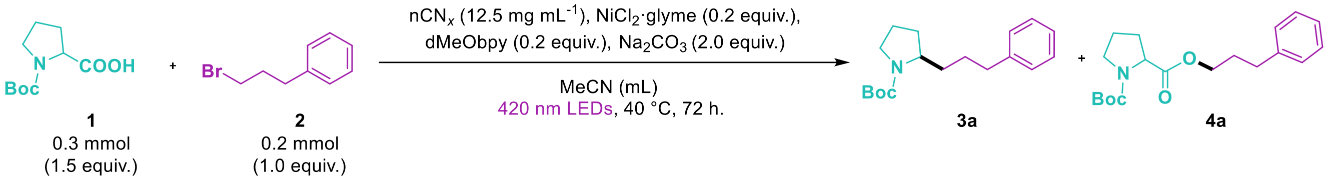

Entry	MeCN (mL)	Yield of **3a**[a]	Yield of **4a**[a]
1	1	30%	27%
2	2	60%	10%
3	4	75%	n.d.[b]

[a]*Yields were calculated by HPLC using a calibration curve.* [b]*Not detected.*

Synthesis and characterization of other catalytic materials.

mpgCN$_x$ was prepared following the reported literature procedure:[1] Melamine (3.0 g; Sigma Aldrich, 99%) and a 40% aqueous dispersion of 12 nm SiO_2 particles (7.5 g; Sigma Aldrich, 100%) were mixed in a 50 mL round-bottom flask and stirred at 70 °C for 16 h. After this time, the resulting mixture was placed into a ceramic crucible and heated to 550 °C (heating ramp: 2.2 °C min^{-1}), then the temperature was maintained at 550 °C for 4 h. The brown-yellow powder was washed with a 20% aqueous solution of NH_4HF_2 (Sigma Aldrich, 95%) and stirred at room temperature for 24 h. The suspension was filtered off, and the solids were washed three times with water and ethanol. Finally, the product was dried under vacuum at 60 °C overnight resulting in 1.3 g of mpgCN$_x$.[2] BET adsorption isotherms demonstrated a surface area of 184 m^2 g^{-1}. (Figure S2a) XRD diffractograms revealed two characteristic diffraction peaks at $2\theta = 13°$ and 27° corresponding, respectively, to the (100) and (002) planes of carbon nitride (Figure S2b). The C/N ratio was found to be 0.65, close to the ideal value of 0.7 (Table S8).

K-PHI was prepared following the reported literature procedure:[3] Melamine (4g; Sigma Aldrich, 99%) and KCl (6 g; Sigma Aldrich, 99%) were grinded in a pestle and mortar. The mixture was placed to a ceramic crucible containing $Mg(OH)_2$ (8 g; Sigma Aldrich, 99%) and heated to 550 °C (heating ramp: 15 °C min^{-1}), then the temperature was maintained at 550 °C for 4 h. After this time, the yellow powder was washed with washed with water and stirred in water for 10 h. The suspension was filtrated off and washed three times with water and ethanol. Finally, the product was dried under vacuum at 60 °C overnight resulting in 2.3 g of K-PHI.[4] BET adsorption

isotherms demonstrated a surface area of 32 m^2 g^{-1} (Figure S2c). XRD diffractograms revealed two characteristic diffraction peaks at $2\theta = 8°$ and 27° corresponding, respectively, to the (110) and (002) planes of carbon nitride (Figure S2d). The C/N ratio was found to be 0.64, close to the ideal value of 0.75. (Table S9). Additionally, ICP analysis showed a 7 wt% loading of potassium (Table S8).

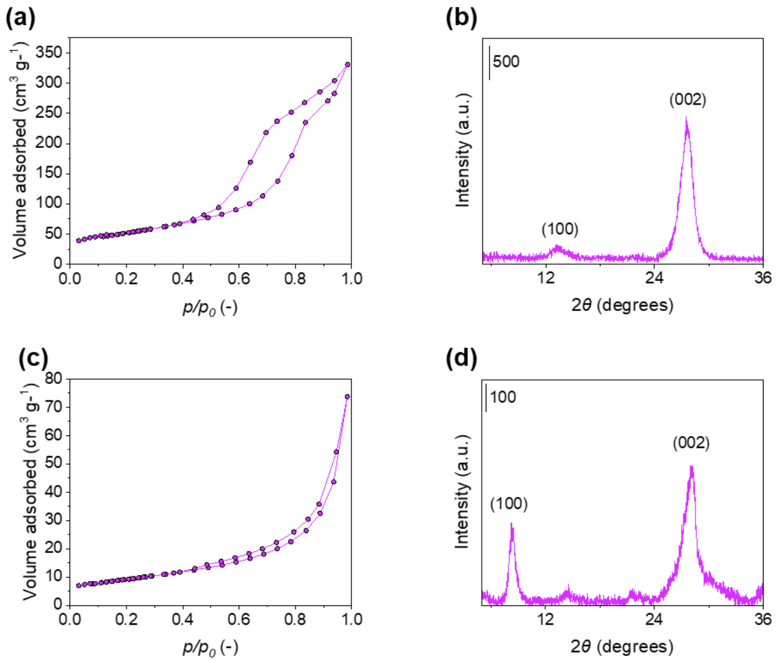

Figure S2. Alternative photocatayst characterizations. *N_2 physisorption isotherm. (**a**) and XRD pattern (**b**) of mpgCN$_x$. N_2 physisorption isotherm (**c**) and XRD pattern (**d**) of K-PHI.*

Table S8. *ICP-OES and CHN analysis of the samples.*

Entry	Catalyst	C[a]	N[a]	H[a]	K[b]
1	mpgCN$_x$	34.39 ± 0.3	52.96 ± 0.5	2.09 ± 0.1	n.d.[c]
2	K-PHI	26.02 ± 0.3	40.36 ± 0.5	1.82 ± 0.1	7.26 ± 0.1

[a]From CHN combustion analysis. [b]From ICP-OES analysis. [c]Not detected.

Recyclability tests and post-catalysis characterization.

nCN_x was recycled across five consecutive cycles under optimized conditions, consistently achieving the alkylated product **3a** in yields ranging from 77% to 81% (Table S9). After each reaction, the nCN_x catalyst was recovered via centrifugation, thoroughly washed using sonication with ethyl acetate (5 mL × 3; Sigma Aldrich, 99%) and water (5 mL × 3), and then dried overnight at 65 °C. The recovery rate of the catalyst was monitored, yielding quantitative recovery in each cycle.

Table S9. *nCN_x recyclability tests.*

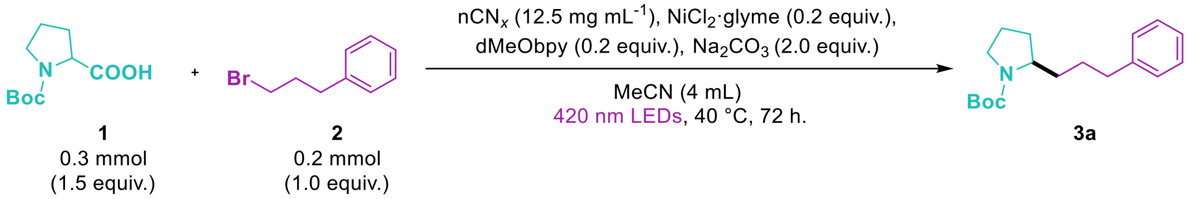

Entry	Number of Cycle	Yield of **3a**[a]
1	1	77%
2	2	78%
3	3	78%
4	4	81%
5	5	75%
6[b]	6a[c]	n.d.[d]
7[b]	6b[e]	72%

[a]*Yields were calculated by HPLC using a calibration curve.* [b]*After the 5 consecutive cycle, we have repeated the experiments two more times, but with modifications from the standard conditions reported in the reaction scheme above.* [c]*No Ni source was added to the reaction this time.* [d]*Not detected.* [e]*The nCN_x catalyst after the 5 consecutive cycles was addionally washed with 1M HCl, and reused for entry 7; the result shows that the activity was recovered.*

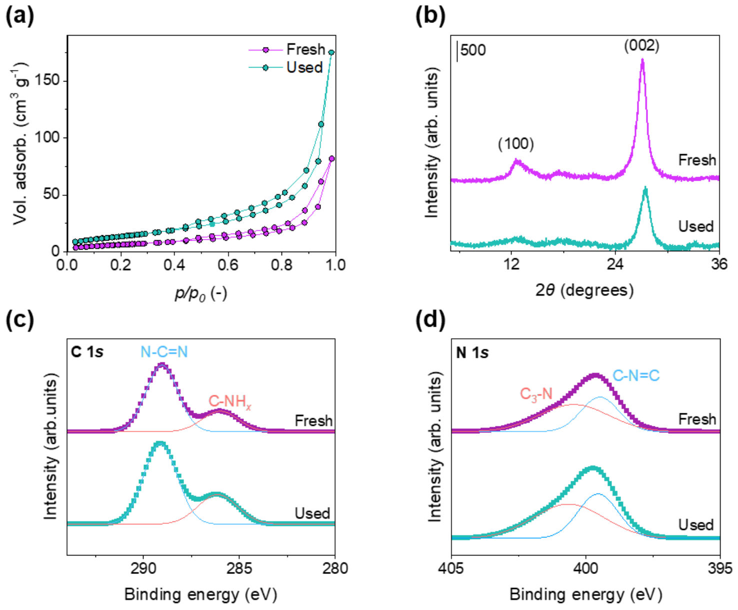

Figure S3. Fresh and recycled nCN$_x$ comparison. *N_2 physisorption isotherms (**a**), XRD patterns (**b**), C 1s XPS **(c)**, and N 1s XPS **(d)** of nCN$_x$ before reaction (fresh, purple) and after 5 consecutive catalytic tests (used, green).*

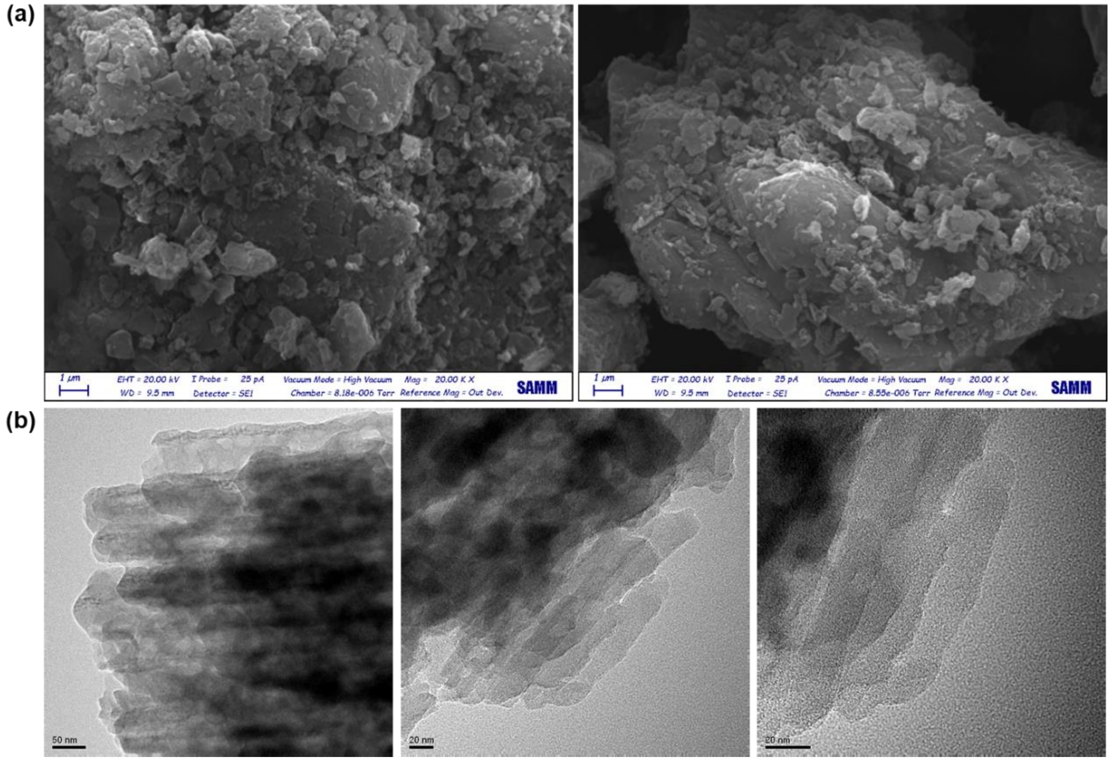

Figure S4. Microscopic analysis of the fresh nCN$_x$. *SEM (**a**, top) and TEM (**b**, bottom) micrographs of nCN$_x$ before reaction.*

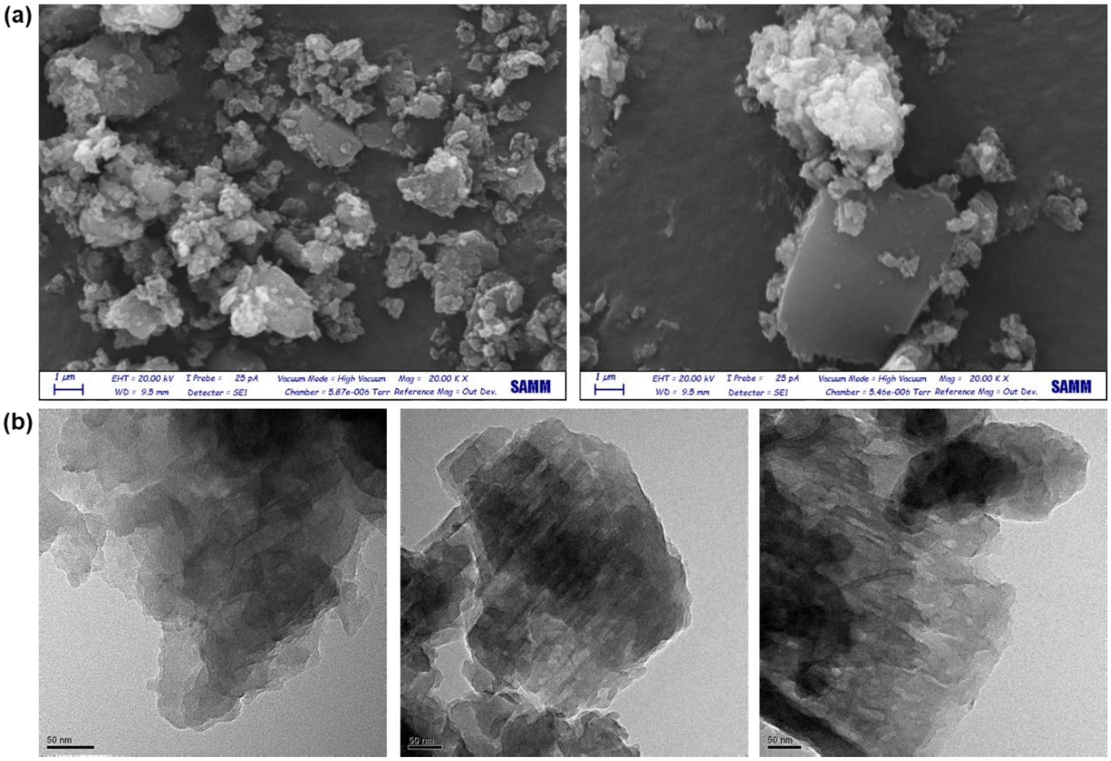

Figure S5. Microscopic analysis of the recycled nCN$_x$. *SEM (**a**) and TEM (**b**) micrographs of nCN$_x$ after 5 consecutive catalytic tests.*

Table S10. *Compositional analysis of the samples.*

Entry	Catalyst	C[a]	N[a]	H[a]	Ni[b]
1	nCN$_x$	34.96 ± 0.3	52.25 ± 0.5	1.87 ± 0.1	n.d.[c]
2	nCN$_x$[d]	31.35 ± 0.3	48.32 ± 0.5	2.80 ± 0.1	0.24 ± 0.1
3	nCN$_x$[e]	31.51 ± 0.3	48.12 ± 0.5	2.78± 0.1	0.01 ± 0.1

[a]*From CHN combustion analysis.* [b]*From ICP-OES analysis.* [c]*No detected.* [d]*The analysis refers to the 'used' sample, after 5 consecutive catalytic tests. The results show no major compositional variation in the sample.* [e]*The analysis refers to the 'used' sample, after 5 consecutive catalytic tests, and addionally washed with 1M HCl.*

Mechanistic studies.

Stern-Volmer experiments. The Stern-Volmer measurements were conducted in a 2 mL suspension of nCN_x in acetonitrile (0.25 mg mL^{-1}). The measurements were carried out in a 1 cm pathlength quartz cuvette, with continuous stirring of the suspension to ensure homogeneity. The sample was excited using a 375 nm continuous-wave (CW) laser operating at a power of 550 μW. The emitted light was filtered through a dichroic mirror to eliminate scattered laser light before being collected by a Maya1000 visible spectrometer. The excitation wavelength of 375 nm was specifically chosen to avoid overlap or interference with the nCN_x emission spectra. For each experiment, a 1000 μM stock solution of the molecule to analyze was prepared in a 1:1 mixture of water and acetonitrile. Incremental additions of 20 μL of this stock solution were made every 5 minutes to the cuvette containing the nCN_x suspension, resulting in a stepwise concentration increase of 10 μM per addition.

Stern-Volmer quenching constant K_{SV} of each experiment was calculated (Table S11). Within the concentration range where the relationship between fluorescence intensity and quencher concentration remains linear (Figure S6h), the quenching constant is purely dynamic and the following equation can be applied:

$$\frac{I_0}{I} = 1 + K_{SV}Q \qquad \text{(Eq. 1)}$$

where I_0 and I are the intensities of the emission of the nCN_x solution obtained by integrating the emission spectra without and with quencher respectively and Q is the concentration of the quencher molecule. In this range, the shape and the position of the emission peak don't change with the addition of the quencher (Figure S6g), this means that the environment is not affected by the addition of quencher molecules.

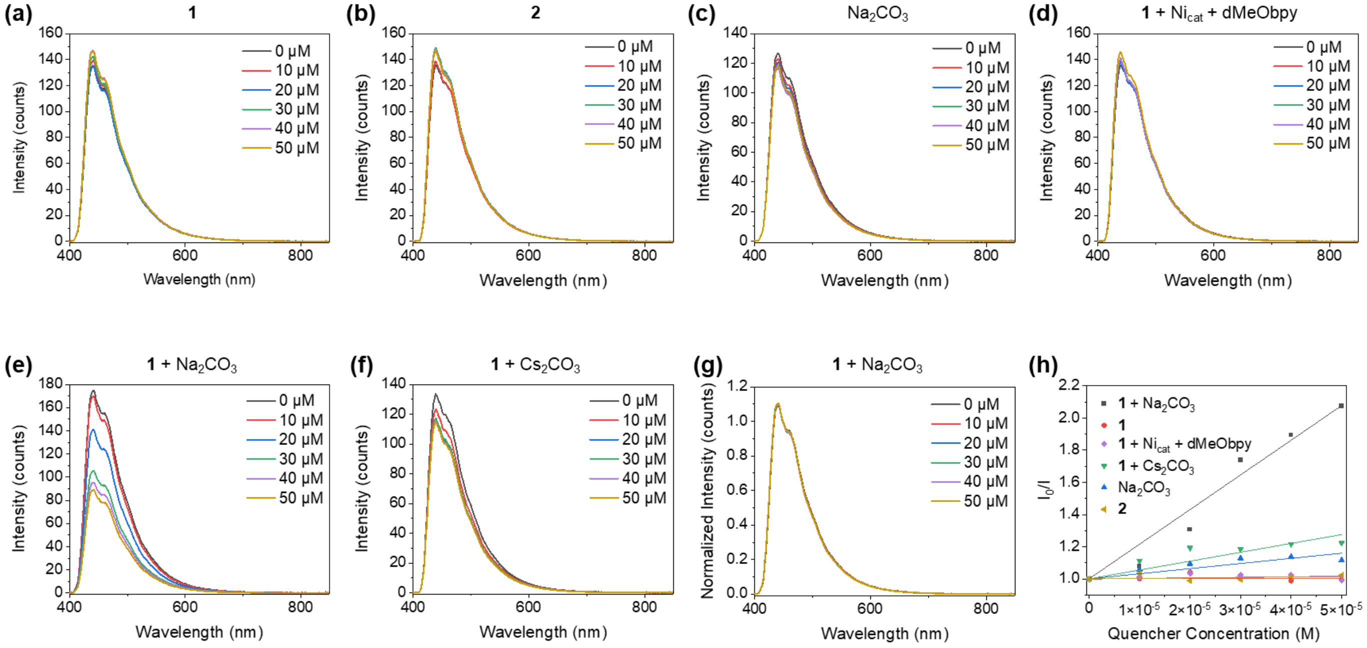

Figure S6. Stern-Vomer quenching spectra of different components of the reactions. *Boc-Pro-OH,* ***1*** *(**a**), 1-bromo-3-phenylpropane,* ***2*** *(**b**),* Na_2CO_3 *(**c**),* ***1*** *with* $NiCl_2$*·glyme and 4,4'-dimethoxy-2,2'-bipyridine 1:0.2:0.2 in molar ratio (**d**),* ***1*** *with* Na_2CO_3 *1:1.5 in molar ratio (**e**),* ***1*** *with* Cs_2CO_3 *in 1:1.5 molar ratio (**f**), the normalized fluorescence intensity of* ***1*** *with* Na_2CO_3 *1:1.5 in molar ratio (**g**), and quenching plots of each spectrum (**h**).*

Table S11. *Stern-Volmer quenching constant for the interaction with different molecules extrapolated by the fitting in Figure S6.*

Quenching experiment	K_{SV} [M^{-1}]
**1** + Na_2CO_3	2.15×10^4
**1** + Cs_2CO_3	5.5×10^3
**1**	101 (0)
**2**	294 (0)
Na_2CO_3	3.2×10^3
**1** + dMeObpy + $NiCl_2$·glyme	351 (0)

Radial trap experiments. For the experiment to evidence *radical proline formation*, in an 8 mL vial were added Boc-Pro-OH (0.3 mmol, 1.5 equiv.; BLD, 99%), $NiCl_2$·glyme (0.04 mmol, 0.2 equiv.; Sigma Aldrich, 99%), dMeObpy (0.04 mmol, 0.2 equiv.; Sigma Aldrich, 99%), nCN_x (50 mg) and Na_2CO_3 (0.4 mmol, 2.0 equiv.; Thermo Fisher, 99%). The mixture was dissolved in MeCN (4 mL; Sigma Aldrich, 99%) and, to the suspension, TEMPO

(0.2 mmol, 1.0 equiv.) was added. The vial was closed with a screw cap rubber septum and inserted in the photoreactor. The suspension was vigorously stirred under purple light (420 nm) at 40 °C for 72 h. After this time, the crude was filtered off though a PTFE syringe filter (0.45 µm) and washed with a saturated aqueous solution of $NaHCO_3$. The aqueous layer was washed with more EtOAc (3 × 5 mL). The organic layers were collected and dried over $MgSO_4$. The solvent was removed *in vacuo* to afford **6** (76 mg, 75% pure, 72% yield) as a pink oil. The ^{1}H-NMR data is consistent with literature precedent.[5]

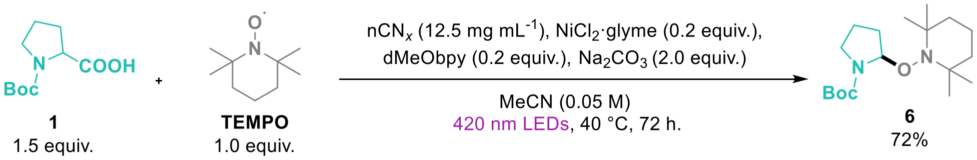

Figure S7. Radical formation experiment. *The ^{1}H-NMR yield was obtained using TMB as internal standard.*

For the experiment to evidence *the role of the radical proline*, in an 8 mL vial were added Boc-Pro-OH (0.3 mmol, 1.5 equiv.; BLD, 99%), $NiCl_2$·glyme (0.04 mmol, 0.2 equiv.; Sigma Aldrich, 99%), dMeObpy (0.04 mmol, 0.2 equiv.; Sigma Aldrich, 99%), nCN_x (50 mg) and Na_2CO_3 (0.4 mmol, 2.0 equiv.; Thermo Fisher, 99%). The mixture was dissolved in MeCN (4 mL Sigma Aldrich, 99%) and, to the suspension, 1-bromo-3-phenylpropane (0.2 mmol, 1.0 equiv.; Sigma Aldrich, 98%) and TEMPO (0.2 mmol, 1.0 equiv.) were added. The vial was closed with a screw cap rubber septum and inserted in the photoreactor. The suspension was vigorously stirred under purple light (420 nm) at 40 °C for 72 h. After this time, the crude was filtered off though a PTFE syringe filter (0.45 µm) and a sample was taken to check ^{1}H-NMR yield.

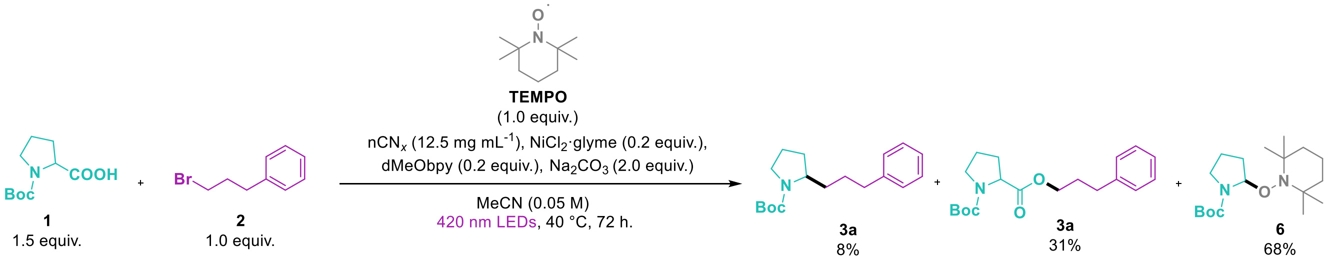

Figure S8. Radical quenching experiment. *The ^{1}H-NMR yield was obtained using TMB as internal standard.*

EPR experiments. EPR spectroscopy was employed to monitor the oxidation state of Ni ions. Ni(II) ions (d^8) exhibit an S = 1 spin state in octahedral or tetrahedral coordination, but adopt a low-spin (S = 0, EPR silent) configuration in square planar geometry. Due to large zero-field splitting (D), X-band (9.5 GHz) EPR spectra of S = 1, Ni(II) are often unobservable, except in cases of relatively high local symmetry.[6–8] Conversely, Ni(I) (d^9) and Ni(III) (d^7) exhibit distinct EPR spectra whose features depend on coordination environment.[9,10]

In order to verify the possibility to detect the presence of Ni(II), the $NiCl_2$·glyme precursor was dissolved in acetonitrile, the reaction solvent. The resulting EPR spectrum, recorded at 77 K, is reported in Figure S9a and shows a broad absorption signal centered at g = 2.245, consistent with an S = 1 Ni(II) species in a tetrahedral environment and small zero field splitting.[8,11] While the complete determination of the spin-Hamiltonian parameters is not possible at X-band frequency and would require high-field experiments, these results confirm the detectability of Ni(II) species under the current conditions. The spectrum of the reaction mixture, recorded before starting the photocatalytic reaction, is reported in black. The spectrum shows features similar to those of the Ni precursor indicating the presence of EPR active (S=1) Ni(II) species. After starting the photocatalytic reaction, sample aliquots were successively taken at different time intervals (Figures S9b,c). The intensity of the EPR signal was observed to decrease as a function of time and reach an almost stable value after 2 h, as a steady state is reached. After prolonged irradiation the observation of the carbon nitride paramagnetic defect becomes observable (asterisk in Figure S9b).[12]

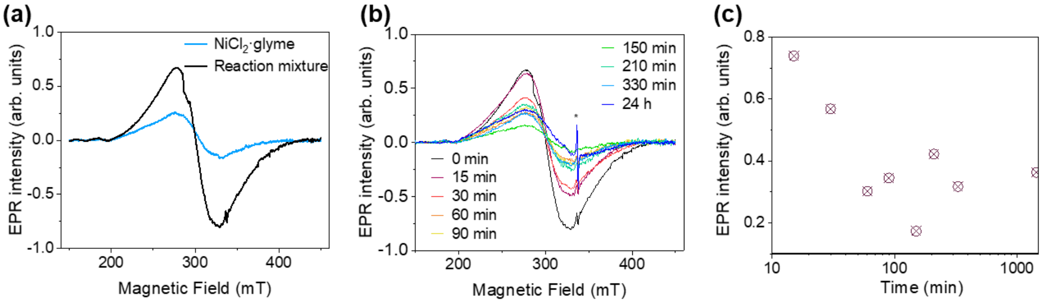

Figure S9*. *EPR experiments. *CW-EPR spectra of the Ni precursor in MeCN (light blue curve) and of the reaction mixture (black curve), at 77 K with 10 mW microwave power (**a**). X-band EPR spectra of the reaction mixture taken at subsequent irradiation time **(b)**; the asterisk in (b) indicates the signal of the CN radical defect. Integrated intensity of the EPR signal is plotted as a function of the irradiation time (**c**).*

DFT calculations. Spin-polarization Density Functional Theory (DFT) calculations were performed with the VASP code.[13–15] The electrons for each element were treated explicitly within the recommended VASP pseudopotentials, and the interaction between the inner electrons and the nuclei was treated with the Projector Augmented Wave method.[16,17] The exchange-correlation functional proposed by Perdew, Burke, and Ernzerhof (PBE) was adopted.[18] To account for the van der Waals interaction, the long-range dispersion was added to the potential according to Grimme's DFT+D3 scheme with a Becke-Johnson damping function.[19,20] For all the structures, single point calculations on top of PBE+D3 results were done with the hybrid functional PBE0[21,22] to improve the electronic structure description. The catalyst and reactants were simulated in a square box, 30×30Å, and the structure relaxations were performed at the G point with a cutoff of 400 eV recurring to a conjugated gradient algorithm. Benchmark calculations were performed to ensure that the Plane-Wave approach is suitable to describe the molecular system.[23] The threshold criteria for electronic and ionic loops were set to 1×10^{-5} eV and 1×10^{-2} eV/Å, respectively. Gibbs free energies profiles have been determined by adopting the computational approach pioneered by Nørskov and co-workers including zero-point energy (ZPE) correction and entropy terms.[24,25] The ZPE was calculated within the harmonic approximation, and the entropies for each species involved in the reaction were calculated through the formalism of the partition function. For adsorbates, the vibrational entropy contribution was considered.

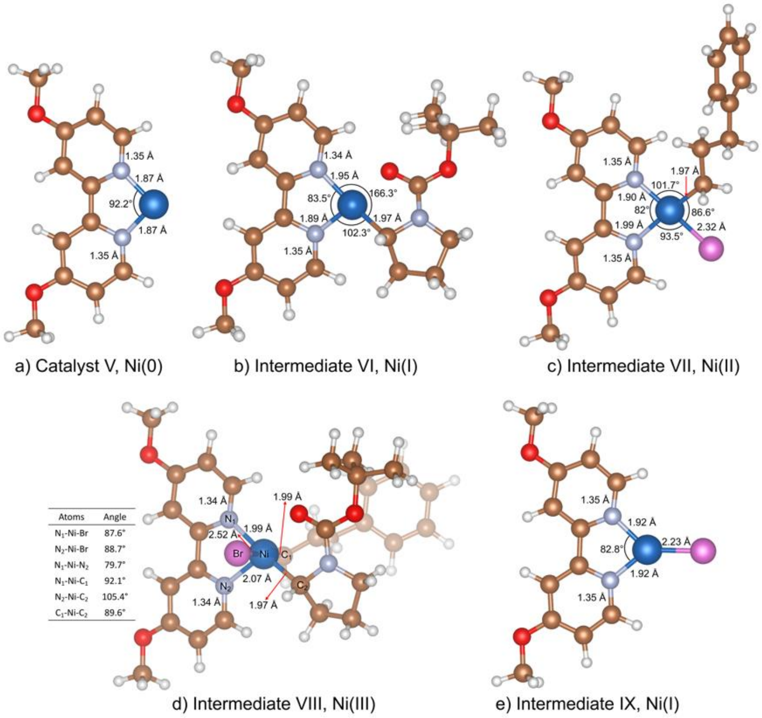

Figure S10. Bond distance and angles for the catalyst and each intermediate during C–C cross-coupling of a carboxylic acid (Boc-L-proline) and an alkyl halide (1-bromo-3-phenylpropane). *Brown, blue, light purple, pink, red, and white balls correspond to carbon, nickel, nitrogen, bromide, oxygen, and hydrogen atoms. Ni(0) featuring catalyst ground state (**a**), Ni(I) featuring intermediate VI bearing the ligand and the decarboxylated Boc-L-proline (**b**), Ni(II) featuring intermediate VII after the oxidative addition step bearing the ligand, bromide and the alkyl moiety (**c**), Ni(III) featuring intermediate VIII bearing the ligand, the decarboxylated Boc-L-proline, the bromide and the alkyl moiety (**d**), Ni(I) featuring intermediate IX after the reductive elimination step bearing the ligand and the bromide (**e**).*

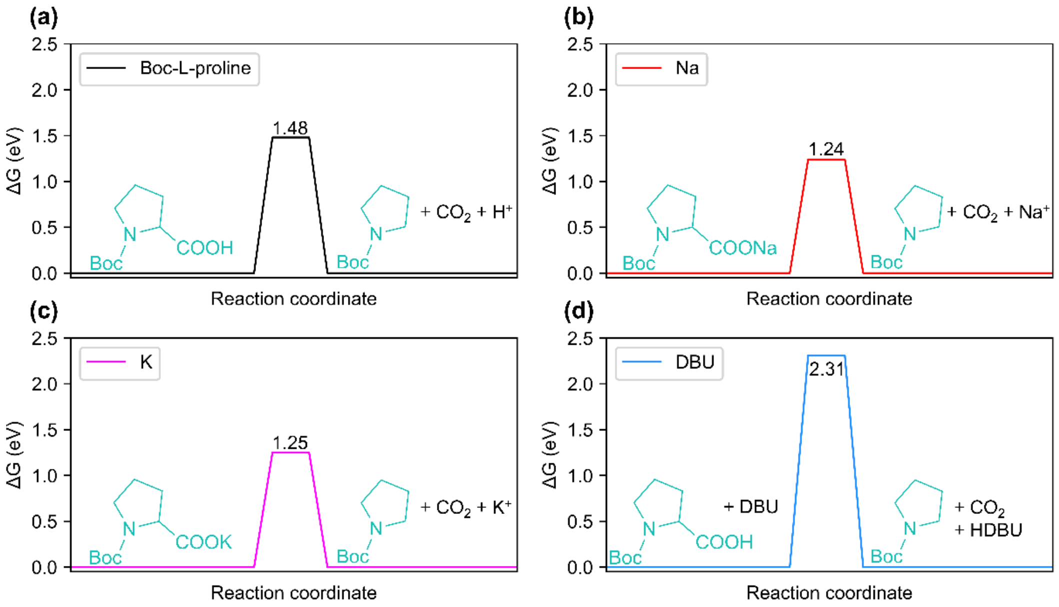

Figure S11. Influence of the presence and type of base in the decarboxylation of Boc-L-proline. *The analysis includes the energy to decarboxylate Boc-L-proline in the absence of any base (**a**), and by considering the interaction between Na*$^{+}$ *cations from Na*$_2$*CO*$_3$ *(**b**), K*$^{+}$ *cations from K*$_2$*CO*$_3$ *(**c**), and DBU (**d**). Deprotonated carboxylic acid are expected in **(b)** and **(c)** because of the type of base in the reaction mixture.*

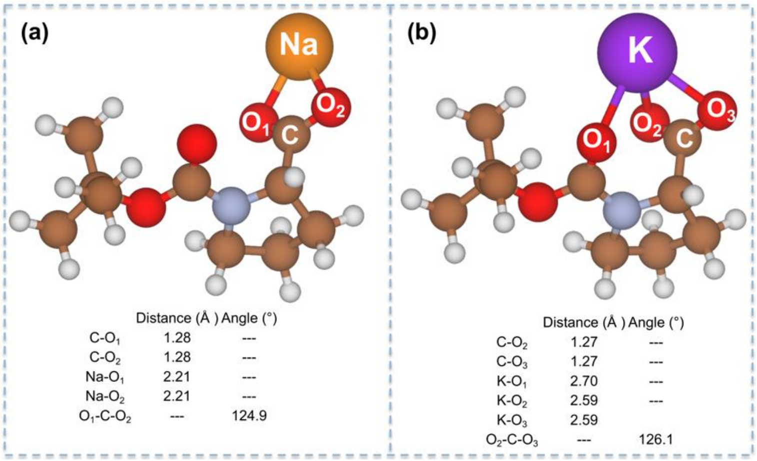

Figure S12. Bond distance and angles obtained from DFT for the decarboxylation of Boc-L-proline. *in the presence of sodium (**a**) and potassium (**b**) containing inorganic bases.*

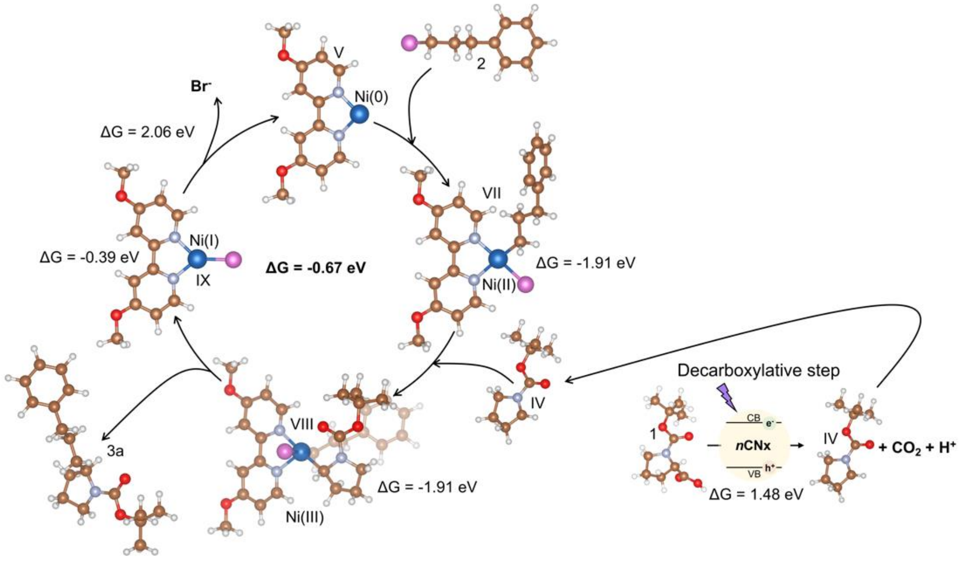

Figure S13. Chemical mechanism for the C–C cross-coupling of a carboxylic acid (Boc-L-proline) and alkyl halide (1-bromo-3-phenylpropane) through the formation of Ni(II) intermediate. *This is the thermodynamically more favorable path. Brown, blue, light purple, pink, red, and white balls correspond to carbon, nickel, nitrogen, bromide, oxygen, and hydrogen atoms.*

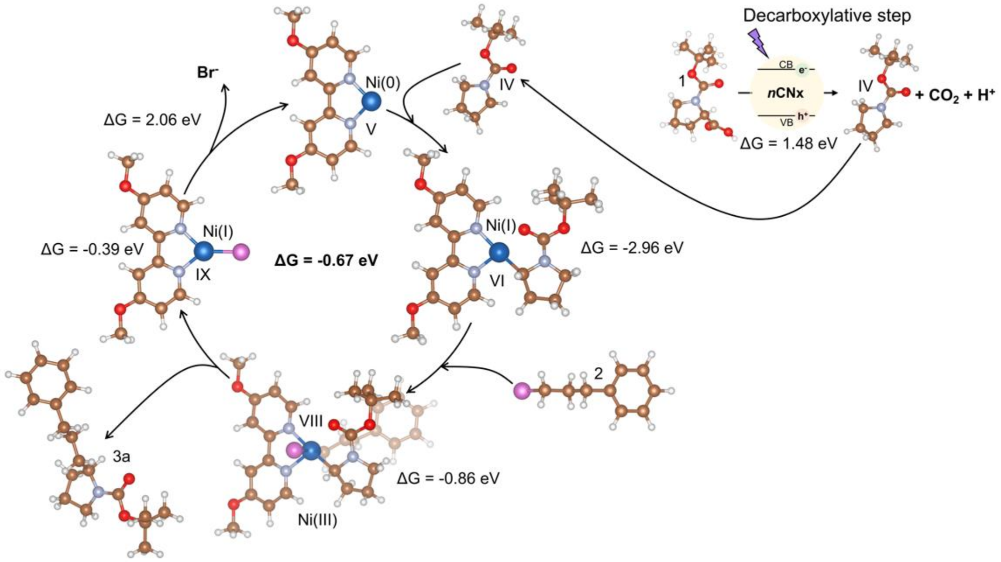

Figure S14. Chemical mechanism for the C–C cross-coupling of a carboxylic acid (Boc-L-proline) and an alkyl halide (1-bromo-3-phenylpropane) through the formation of Ni(II) intermediate. *Brown, blue, light purple, pink, red, and white balls correspond to carbon, nickel, nitrogen, bromide, oxygen, and hydrogen atoms.*

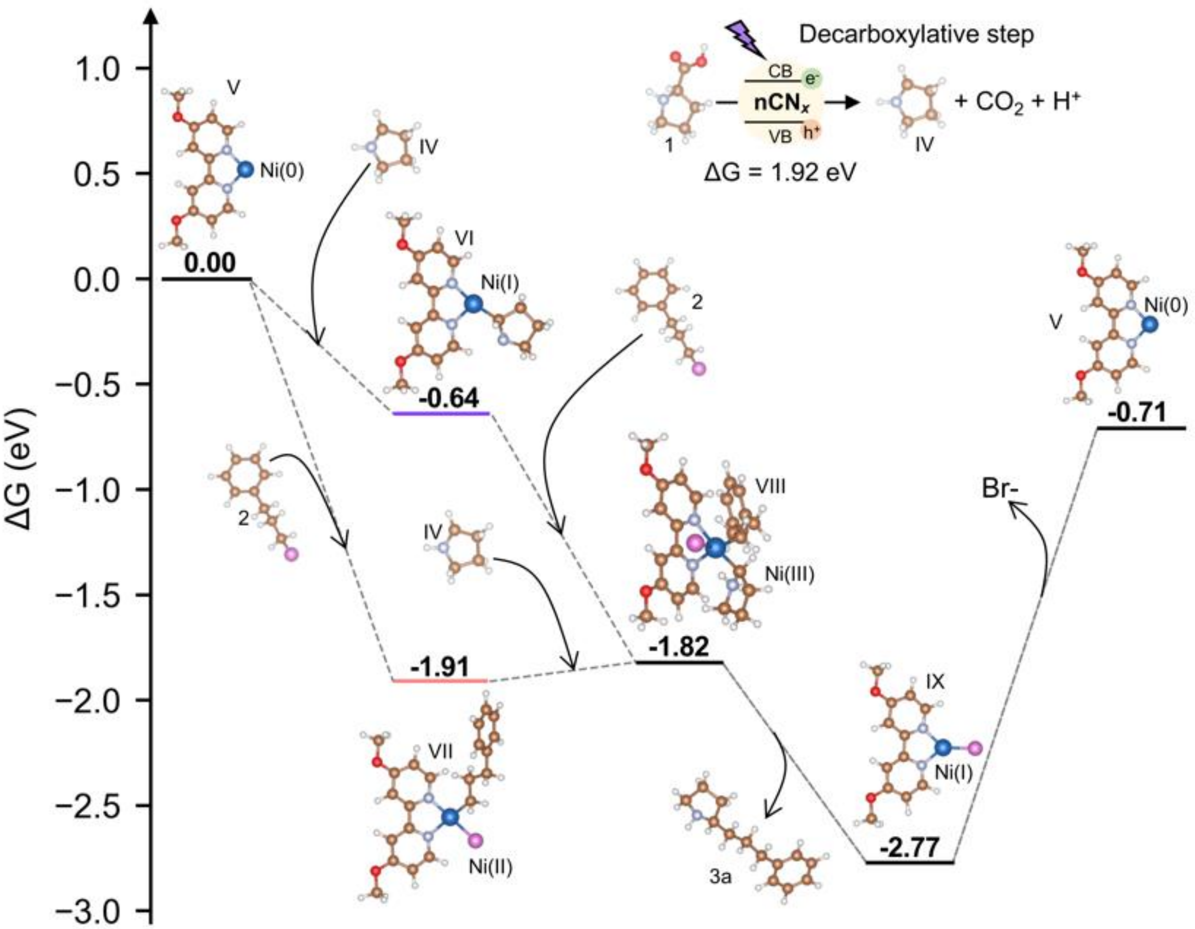

Figure S15. DFT-calculated energy profile for the decarboxylative C–C cross-coupling of a carboxylic acid (proline) and an alkyl halide (1-bromo-3-phenylpropane). *Since proline was employed, the reaction cycle does not consider the presence of a Boc protective group on the carboxylic acid. The insets represent the structures of the key intermediates (**V**, **VI**, **VII**, **VIII,** and **IX**). The red pathway describes a Ni(0)-Ni(II)-Ni(III) oxidation, while the purple pathway illustrates a Ni(0)-Ni(I)-Ni(III) mechanism. Brown, blue, light purple, pink, red, and white balls correspond to carbon, nickel, nitrogen, bromide, oxygen, and hydrogen atoms, respectively.*

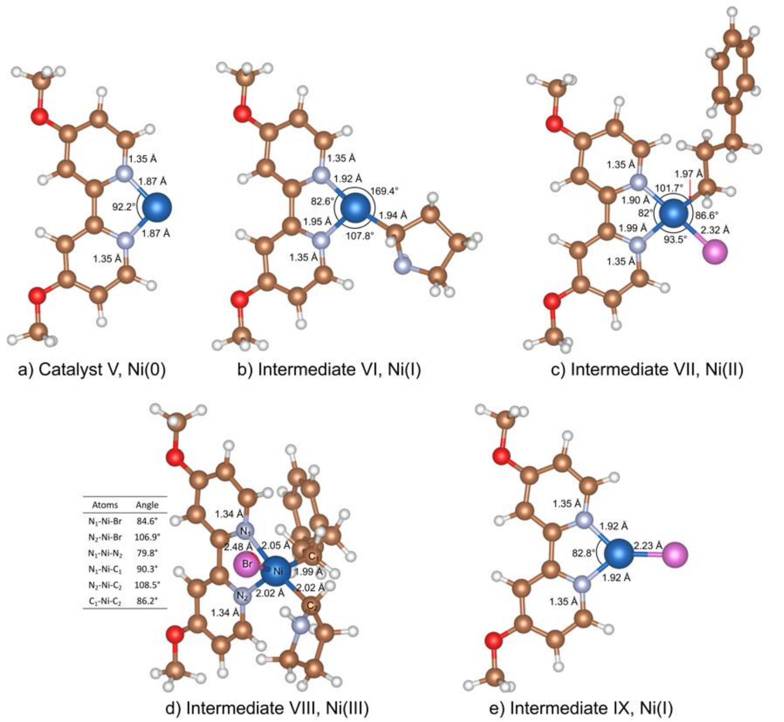

***Figure S16*. Bond distance and angles for the catalyst and each intermediate for the C–C cross-coupling of a carboxylic acid (proline) and an alkyl halide (1-bromo-3-phenylpropane).** *Since proline was employed, the reaction cycle does not consider the presence of a Boc protective group on the carboxylic acid. Brown, blue, light purple, pink, red, and white balls correspond to carbon, nickel, nitrogen, bromide, oxygen, and hydrogen atoms. Ni(0) featuring catalyst ground state bearing the ligand (**a**), Ni(I) featuring intermediate VI bearing the ligand and the decarboxylated proline (**b**), Ni(II) featuring intermediate VII after the oxidative addition step bearing the ligand, bromide and the alkyl moiety (**c**), Ni(III) featuring intermediate VIII bearing the ligand, the decarboxylated proline, the bromide and the alkyl moiety (**d**), Ni(I) featuring intermediate IX after the reductive elimination step bearing the ligand and the bromide (**e**).*

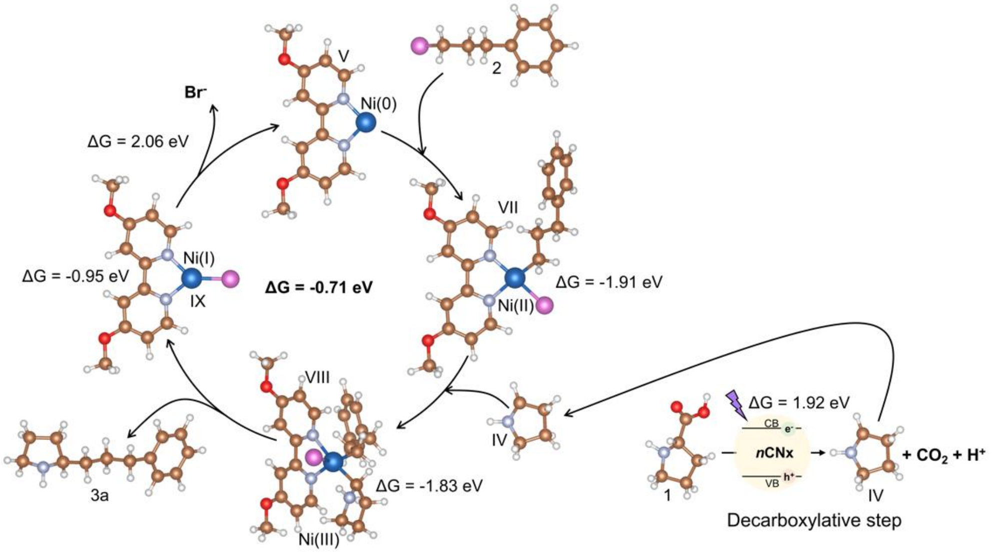

Figure S17. Chemical mechanism for the C–C cross-coupling of a carboxylic acid (proline) and an alkyl halide (1-bromo-3-phenylpropane) through the formation of Ni(II) intermediate. *This is the thermodynamically more favorable path. Since proline was employed, the reaction cycle does not consider the presence of a Boc protective group on the carboxylic acid. Brown, blue, light purple, pink, red, and white balls correspond to carbon, nickel, nitrogen, bromide, oxygen, and hydrogen atoms.*

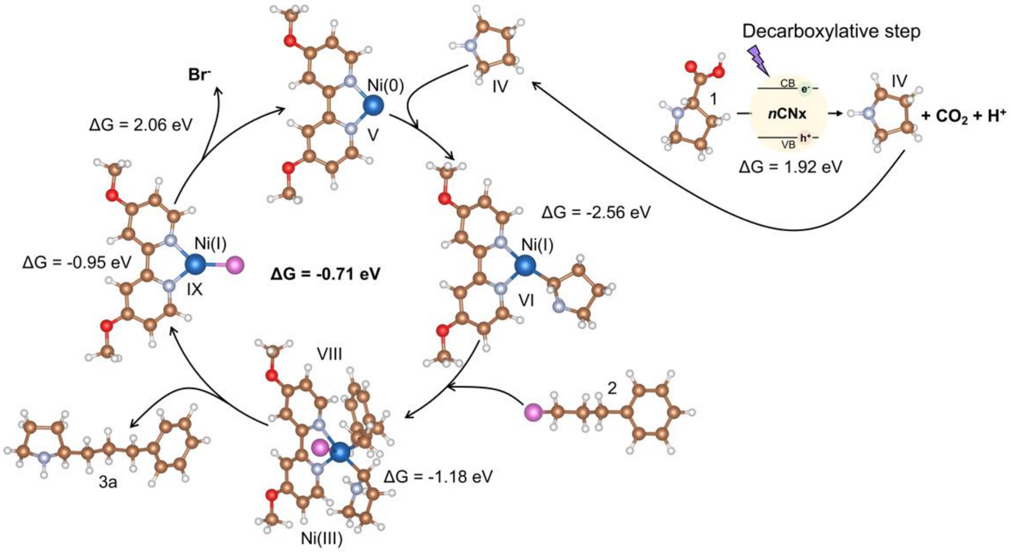

Figure S18. Chemical mechanism for the C–C cross-coupling of a carboxylic acid (proline) and an alkyl halide (1-bromo-3-phenylpropane) through the formation of Ni(I) intermediate. *Since proline was employed, the reaction cycle does not consider the presence of a Boc protective group on the carboxylic acid. Brown, blue, light purple, pink, red, and white balls correspond to carbon, nickel, nitrogen, bromide, oxygen, and hydrogen atoms.*

Life cycle assessment and analysis of the GHG emission potential.

The GHG emissions of the catalytic method were assessed reconstructing the whole synthetic process using the stoichiometric level 3-based LCA method, considering molar ratios, heat requirements, and yields. The Global Warming Potential (GWP100) metric with a 100-year time horizon, measured in kg CO_2 equiv, is employed, as outlined in the 6th IPCC assessment report. We consider a cradle-to-gate approach encompassing emissions associated with the main life cycle stages: material exploitation and acquisition, and production, excluding usage and disposal stages. The functional unit is defined as 1 kg of the targeted component at the end stage of the production process, produced in the European Union. Background life cycle inventories are retrieved from Ecoinvent 3.9.1 cut-off and Carbon Minds databases. Missing components are estimated using a retrosynthetic analysis, in order to deconstruct the respective component into more fundamental building blocks and associated energy requirements.

Experimental data.

General procedure for the decarboxylative alkylation (GP).

In an 8 mL vial were added the corresponding carboxylic acid **1** (0.3 mmol, 1.5 equiv.), $NiCl_2$·glyme (0.04 mmol, 0.2 equiv.; Sigma Aldrich, 99%), dMeObpy (0.04 mmol, 0.2 equiv.; Sigma Aldrich, 99%), nCN$_x$ (50 mg) and Na_2CO_3 (0.4 mmol, 2.0 equiv.; Thermo Fisher, 99%). The mixture was dissolved in MeCN (4 mL; Sigma Aldrich, 99%) and, to the suspension, the corresponding alkyl bromide **2** (0.2 mmol, 1.0 equiv.) was added. The vial was closed with a screw cap rubber septum and inserted in the photoreactor. The suspension was vigorously stirred under purple light (420 nm) at 40 °C for 72 h. After this time, the crude was filtered off through a PTFE syringe filter (0.45 µm) and washed with a saturated aqueous solution of $NaHCO_3$ (Sigma Aldrich, 99%). The aqueous layer was washed with more EtOAc (3 × 5 mL). The organic layers were collected and dried over $MgSO_4$ (Sigma Aldrich, 99%). The organic phase was thus purified *via* silica gel column chromatography to give product **3**.

***tert*-Butyl 2-(3-phenylpropyl)pyrrolidine-1-carboxylate (3a)**

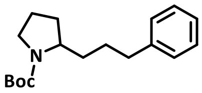

The reaction was performed according to the GP, using Boc-Pro-OH (0.3 mmol, 1.5 equiv.; BLD, 99%), $NiCl_2$·glyme (0.2 equiv.; Sigma Aldrich, 99%), dMeObpy (0.2 equiv.; Sigma Aldrich, 99%), nCN_x (50 mg), Na_2CO_3 (2.0 equiv.; Thermo Fisher, 99%) and 1-bromo-3-phenylpropane (1.0 equiv.; Sigma Aldrich, 98%) in 4 mL of MeCN (Sigma Aldrich, 99%). The mixture was purified via silica gel column chromatography (5 to 10% EtOAc/Hexane) to give product **3a** as a colorless oil (41 mg, 71% yield). Large scale reaction was carried out in a 50 mL round bottom flask, where Boc-Pro-OH (3 mmol, 1.5 equiv.; BLD, 99%), $NiCl_2$·glyme (0.2 equiv.; Sigma Aldrich, 99%), dMeObpy (0.2 equiv.; Sigma Aldrich, 99%), nCN_x (500 mg), Na_2CO_3 (2.0 equiv.; Thermo Fisher, 99%) and 1-bromo-3-phenylpropane (1.0 equiv.; Sigma Aldrich, 98%), were added in 40 mL of MeCN (Sigma Aldrich, 99%). Four kessil lamps (427 nm) at 6 cm distance, and with a fan placed above for cooling reasons, irradiated the suspension, which was vigorously stirred for 72 h, obtaining **3a** (362 mg, 63% yield).

R_f (10% EtOAc/Hexane): 0.3

^{1}H NMR (400 MHz, $CDCl_3$): δ 7.30 – 7.26 (2H, m), 7.19 – 7.16 (3H, m), 3.77 (1H, tt, *J* = 7.7, 3.5 Hz), 3.40 – 3.26 (2H, m), 2.63 (2H, hept, *J* = 6.9 Hz), 1.96 – 1.83 (1H, m), 1.82 – 1.72 (3H, m), 1.67 – 1.57 (3H, m), 1.44 (9H, s), 1.41 – 1.28 (1H, m)

^{13}C NMR (101 MHz, $CDCl_3$): δ 154.8, 142.7, 128.5, 128.4, 125.8, 79.0, 57.3, 46.4, 36.1, 34.3, 30.5, 28.7, 28.3, 23.5.

This data is consistent with literature precedent.[26]

***tert*-Butyl 2-(3-phenylpropyl)azetidine-1-carboxylate (3b)**

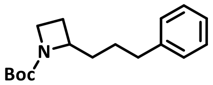

The reaction was performed according to the GP, using boc-azetidine-2-carboxylic acid (0.3 mmol, 1.5 equiv.; BLD, 95%), $NiCl_2$·glyme (0.2 equiv.; Sigma Aldrich, 99%), dMeObpy (0.2 equiv.; Sigma Aldrich, 99%), nCN_x (50 mg), Na_2CO_3 (2.0 equiv.; Thermo Fisher, 99%) and 1-bromo-3-phenylpropane (1.0 equiv.; Sigma Aldrich, 98%) in 4 mL of MeCN (Sigma Aldrich, 99%). The mixture was

purified via silica gel column chromatography (5 to 10% EtOAc/Hexane) to give product **3b** as a colorless oil (35 mg, 64% yield).

R_f (10% EtOAc/Hexane): 0.3.

^{1}H NMR (400 MHz, $CDCl_3$) δ 7.30 – 7.26 (2H, m), 7.20 – 7.16 (3H, m), 4.24 – 4.17 (1H, m), 3.85 – 3.74 (2H, m), 2.67 – 2.62 (2H, m), 2.27 – 2.19 (1H, m), 1.96 – 1.86 (1H, m), 1.84 – 1.76 (1H, m), 1.71 – 1.61 (3H, m), 1.42 (9H, s).

^{13}C NMR (101 MHz, $CDCl_3$): δ 156.8, 142.5, 128.5, 128.4, 125.9, 79.2, 62.1, 46.5, 36.0, 35.1, 28.6, 26.5, 22.0.

This data is consistent with literature precedent.[26]

***tert*-Butyl 2-(3-phenylpropyl)piperidine-1-carboxylate (3c)**

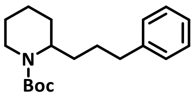

The reaction was performed according to the GP, using Boc-Pip-OH (0.3 mmol, 1.5 equiv.; BLD, 95%), $NiCl_2$·glyme (0.2 equiv.; Sigma Aldrich, 99%), dMeObpy (0.2 equiv.; Sigma Aldrich, 99%), nCN_x (50 mg), Na_2CO_3 (2.0 equiv.; Thermo Fisher, 99%) and 1-bromo-3-phenylpropane (1.0 equiv.; Sigma Aldrich, 98%) in 4 mL of MeCN (Sigma Aldrich, 99%). The mixture was purified via silica gel column chromatography (5 to 10% EtOAc/Hexane) to give product **3c** as a colorless oil (34 mg, 56% yield).

R_f (10% EtOAc/Hexane): 0.3.

^{1}H NMR (400 MHz, $CDCl_3$) δ 7.29 – 7.24 (2H, m), 7.19 – 7.15 (3H, m), 4.24 (1H, br s), 3.96 (1H, d, J = 13.2 Hz), 2.74 – 2.58 (3H, m), 1.76 – 1.67 (1H, m), 1.65 – 1.48 (8H, m), 1.44 (9H, s), 1.43 – 1.35 (1H, m).

^{13}C NMR (101 MHz, $CDCl_3$): δ 155.3, 142.6, 128.5, 128.4, 125.8, 79.2, 50.4, 38.9, 35.8, 29.3, 28.8, 28.6, 28.1, 25.8, 19.2.

This data is consistent with literature precedent.[26]

***tert*-Butyl (5-phenylpentan-2-yl)carbamate (3d)**

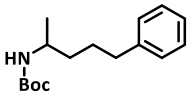

The reaction was performed according to the GP, using Boc-Ala-OH (0.3 mmol, 1.5 equiv.; BLD, 95%), $NiCl_2$·glyme (0.2 equiv.; Sigma Aldrich, 99%), dMeObpy (0.2 equiv.; Sigma Aldrich, 99%), nCN_x (50 mg), Na_2CO_3 (2.0 equiv.; Thermo Fisher, 99%) and 1-bromo-3-phenylpropane (1.0

equiv.; Sigma Aldrich, 98%) in 4 mL of MeCN (Sigma Aldrich, 99%). The mixture was purified via silica gel column chromatography (5 to 10% EtOAc/Hexane) to give product **3d** as a colorless oil (38 mg, 72% yield).

R_f (10% EtOAc/Hexane): 0.3.

^{1}H NMR (400 MHz, $CDCl_3$) δ 7.29 – 7.25 (2H, m), 7.19 – 7.16 (3H, m), 4.27 (1H, br s), 3.67 (1H, br s), 2.68 – 2.56 (2H, m) 170 – 1.58 (3H, m), 1.44 (10H, s) 1.1 (3H, d, J = 6.5 Hz).

^{13}C NMR (101 MHz, $CDCl_3$): δ 154.4, 141.3, 127.4, 127.3, 124.7, 78.0, 45.3, 35.9, 34.7, 27.4, 26.8, 20.3.

This data is consistent with literature precedent.[26]

***tert*-Butyl (2-methyl-6-phenylhexan-3-yl)carbamate (3e)**

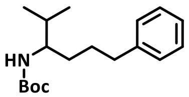

The reaction was performed according to the GP, using Boc-Val-OH (0.3 mmol, 1.5 equiv.; BLD, 97%), $NiCl_2$·glyme (0.2 equiv.; Sigma Aldrich, 99%), dMeObpy (0.2 equiv.; Sigma Aldrich, 99%), nCN_x (50 mg), Na_2CO_3 (2.0 equiv.; Thermo Fisher, 99%) and 1-bromo-3-phenylpropane (1.0 equiv.; Sigma Aldrich, 98%) in 4 mL of MeCN (Sigma Aldrich, 99%). The mixture was purified via silica gel column chromatography (5 to 10% EtOAc/Hexane) to give product **3e** as a colorless oil (39 mg, 67% yield).

R_f (10% EtOAc/Hexane): 0.3.

^{1}H NMR (400 MHz, $CDCl_3$) δ 7.29 – 7.25 (2H, m), 7.19 – 7.16 (3H, m), 4.24 (1H, br d, J = 9.4 Hz), 3.48 (1H, dt, J = 10.2, 5.2 Hz), 2.70 – 2.54 (2H, m), 1.72 – 1.60 (3H, m), 1.44 (10H, s), 1.33 – 1.24 (1H, m), 0.86 (6H, dd, J = 15.9, 6.9 Hz).

^{13}C NMR (101 MHz, $CDCl_3$), rotameric mixture, resonances for minor rotamer are enclosed in parenthesis: δ 156.2, 142.6, 128.6, 128.4, 125.8, 79.0, 55.4, 35.8, 32.3, (32.3), 28.6, (28.5), 28.2, 20.1, 19.3, (17.7)

***tert*-butyl (1,5-diphenylpentan-2-yl)carbamate (3f)**

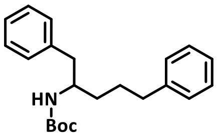

The reaction was performed according to the GP, using Boc-Phe-OH (0.3 mmol, 1.5 equiv.; BLD, 97%), $NiCl_2$·glyme (0.2 equiv.; Sigma Aldrich, 99%), dMeObpy (0.2 equiv.; Sigma Aldrich, 99%), nCN_x (50 mg), Na_2CO_3 (2.0 equiv.; Thermo Fisher, 99%) and 1-bromo-3-phenylpropane (1.0 equiv.; Sigma Aldrich, 98%) in 4 mL of MeCN (Sigma Aldrich, 99%). The mixture was

purified via silica gel column chromatography (5 to 10% EtOAc/Hexane) to give product **3f** as a colorless oil (33 mg, 48% yield).

R_f (10% EtOAc/Hexane): 0.2.

^{1}H NMR (400 MHz, $CDCl_3$) δ 7.30 – 7.12 (10H, m), 4.26 (1H, br s), 3.86 (1H, br s), 2.75 (2H, d, *J*= 6.3 Hz) 2.66 – 2.51 (2H, m), 1.72 – 1.60 (2H, m), 1.57 – 1.49 (1H, m), 1.44 – 1.24 (10H, m).

^{13}C NMR (101 MHz, $CDCl_3$): δ 155.6, 142.4, 138.4, 129.7, 129.0, 128.6, 128.5, 126.4, 125.9, 77.4, 51.5, 41.6, 35.7, 33.9, 28.5, 28.0.

This data is consistent with literature precedent.[26]

Benzyl *tert*-butyl (8-phenyloctane-1,5-diyl)dicarbamate (3g)

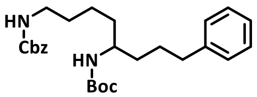

The reaction was performed according to the GP, using Boc-Lys(Z)-OH (0.3 mmol, 1.5 equiv.; BLD, 95%), $NiCl_2$·glyme (0.2 equiv.; Sigma Aldrich, 99%), dMeObpy (0.2 equiv.; Sigma Aldrich, 99%), nCN_x (50 mg), Na_2CO_3 (2.0 equiv.; Thermo Fisher, 99%) and 1-bromo-3-phenylpropane (1.0 equiv.; Sigma Aldrich, 98%) in 4 mL of MeCN (Sigma Aldrich, 99%). The mixture was purified via silica gel column chromatography (5 to 20% EtOAc/Hexane) to give product **3g** as a colorless oil (66 mg, 74% yield).

R_f (10% EtOAc/Hexane): 0.1.

^{1}H NMR (400 MHz, $CDCl_3$) δ 7.36 (3H, d, *J* = 4.4 Hz), 7.34 – 7.28 (1H, m), 7.27 – 7.25 (3H, m), 7.19 – 7.15 (3H, m), 5.09 (2H, s), 4.80 (1H, br s), 4.22 (1H, br s), 3.58 (1H, s), 3.18 (2H, q, *J* = 6.5 Hz), 1.70 – 1.60 (3H, m), 1.55 – 1.46 (3H, m), 1.43 (9H, s), 1.37 – 1.26 (4H, m).

^{13}C NMR (101 MHz, $CDCl_3$) δ 156.6, 156.0, 142.4, 136.8, 128.6, 128.6, 128.4, 128.3, 128.2, 125.9, 79.2, 66.7, 50.3, 41.0, 35.8, 35.5, 35.4, 29.8, 28.6, 27.9, 23.1.

This data is consistent with literature precedent.[26]

***tert*-Butyl 2-(3-phenylpropyl)octahydro-1H-indole-1-carboxylate (3h)**

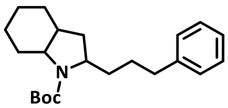

The reaction was performed according to the GP, using Boc-Oic-OH (0.3 mmol, 1.5 equiv.; BLD, 95%), $NiCl_2$·glyme (0.2 equiv.; Sigma Aldrich, 99%), dMeObpy (0.2 equiv.; Sigma Aldrich, 99%), nCN_x (50 mg), Na_2CO_3 (2.0 equiv.; Thermo Fisher, 99%) and 1-bromo-3-phenylpropane (1.0

equiv.; Sigma Aldrich, 98%) in 4 mL of MeCN (Sigma Aldrich, 99%). The mixture was purified via silica gel column chromatography (5 to 10% EtOAc/Hexane) to give product **3h** as a colorless oil (45 mg, 66% yield).

R_f (10% EtOAc/Hexane): 0.3.

^{1}H NMR (400 MHz, $CDCl_3$) δ 7.29 – 7.25 (2H, m), 7.19 – 7.15 (3H, m), 3.68 (2H, d, *J* = 32.7 Hz), 2.63 (2H, dd, *J* = 15.9, 8.0 Hz), 2.34 – 1.87 (4H, m), 1.65 – 1.57 (6H, m), 1.42 – 1.25 (12H, m), 1.16 – 1.02 (2H, m),

^{13}C NMR (101 MHz, $CDCl_3$): δ 154.8, 139.7, 128.5, 128.4, 125.8, 78.8, 57.9, 57.2, 56.8, 36.3, 36.2, 28.7, 26.3, 26.3, 24.3, 24.0, 21.0, 20.8.

***tert*-Butyl 3-(3-phenylpropyl)-2-azabicyclo[2.2.1]heptane-3-carboxylate (3i)**

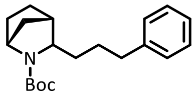

The reaction was performed according to the GP, using N-boc-2-azabicyclo[2.2.1]heptane-3-carboxylic acid (0.3 mmol, 1.5 equiv.; BLD, 95%), $NiCl_2$·glyme (0.2 equiv.; Sigma Aldrich, 99%), dMeObpy (0.2 equiv.; Sigma Aldrich, 99%), nCN$_x$ (50 mg), Na_2CO_3 (2.0 equiv.; Thermo Fisher, 99%) and 1-bromo-3-phenylpropane (1.0 equiv.; Sigma Aldrich, 98%) in 4 mL of MeCN (Sigma Aldrich, 99%). The mixture was purified via silica gel column chromatography (5 to 10% EtOAc/Hexane) to give product **3i** as a colorless oil (49 mg, 77% yield).

R_f (10% EtOAc/Hexane): 0.3.

^{1}H NMR (400 MHz, $CDCl_3$) δ 7.30 – 7.26 (2H, m), 7.20 – 7.16 (3H, m), 4.09 (1H, s), 3.16 (1H, brs), 2.70 – 2.56 (2H, m), 2.31 (1H, d, *J* = 4.1 Hz), 1.81 (1H, brs), 1.74 – 1.56 (6H, m), 1.44 (9H, s), 1.39 – 1.19 (3H, m).

^{13}C NMR (101 MHz, $CDCl_3$): δ 155.2, 142.7, 128.5, 128.4, 125.8, 79.0, 64.8, 57.2, 40.4, 36.2, 34.6, 33.9, 30.3, 29.0, 28.7, 27.9.

***tert*-Butyl 2-(3-phenylpropyl)-3-fluoropyrrolidine-1-carboxylate (3j)**

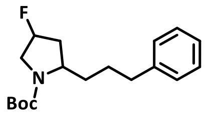

The reaction was performed according to the GP, using N-boc-4-fluoroproline (0.3 mmol, 1.5 equiv.; BLD, 95%), $NiCl_2$·glyme (0.2 equiv.; Sigma Aldrich, 99%), dMeObpy (0.2 equiv.; Sigma Aldrich, 99%), nCN$_x$ (50 mg), Na_2CO_3 (2.0 equiv.; Thermo Fisher, 99%) and 1-bromo-3-phenylpropane (1.0 equiv.; Sigma Aldrich, 98%) in 4 mL of MeCN (Sigma Aldrich, 99%). The mixture was

purified via silica gel column chromatography (5 to 20% EtOAc/Hexane) to give product **3j** as a colorless oil (30 mg, 49% yield).

R_f (20% EtOAc/Hexane): 0.2.

^{1}H NMR (400 MHz, $CDCl_3$) δ 7.29 – 7.25 (2H, m), 7.20 – 7.15 (3H, m), 5.09 (1H, d, J = 52 Hz), 3.98 – 3.85 (2H, m), 3.33 (1H, ddd, *J* = 36.8, 13.3, 3.3 Hz), 2.62 (2H, hept, *J* = 7.0 Hz), 2.43 – 2.31 (1H, m), 1.97 (1H, brs), 1.73 (1H, dddd, *J* = 39.0, 13.6, 8.4, 4.2 Hz), 1.61 – 1.53 (3H, m), 1.44 (9H, s).

^{13}C NMR (101 MHz, $CDCl_3$): δ 154.9, 142.4, 128.5, 128.5, 126.0, 92.6, (90.9), 79.8, 55.8, 53.4, (53.1), 38.8, 36.1, 34.5, 28.6, 27.6.

^{19}F NMR (376 MHz, $CDCl_3$): δ -177.4.

tert-Butyl 2-(3-phenylpropyl)-3-hydroxypyrrolidine-1-carboxylate (3k)

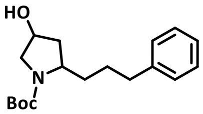

The reaction was performed according to the GP, using N-boc-4-hydroxyproline (0.3 mmol, 1.5 equiv.; BLD, 95%), $NiCl_2$·glyme (0.2 equiv.; Sigma Aldrich, 99%), dMeObpy (0.2 equiv.; Sigma Aldrich, 99%), nCN_x (50 mg), Na_2CO_3 (2.0 equiv.; Thermo Fisher, 99%) and 1-bromo-3-phenylpropane (1.0 equiv. Sigma Aldrich, 98%) in 4 mL of MeCN (Sigma Aldrich, 99%). The mixture was purified via silica gel column chromatography (5 to 20% EtOAc/Hexane) to give product **3k** as a colorless oil (48 mg, 78% yield).

R_f (20% EtOAc/Hexane): 0.2.

^{1}H NMR (400 MHz, $CDCl_3$) δ 7.30 – 7.26 (2H, m), 7.20 – 7.16 (3H, m), 4.40 – 4.34 (1H, m), 3.94 – 3.78 (1H, m), 3.70 – 3.53 (1H, m), 3.31 (1H, ddd, *J* = 49.3, 11.9, 4.1 Hz), 2.74 – 2.57 (2H, m), 2.32 – 1.92 (2H, m), 1.80 – 1.72 (1H, m), 1.67 – 1.52 (3H, m), 1.43 – 1.25 (10H, m).

^{13}C NMR (101 MHz, $CDCl_3$), rotameric mixture, resonances for minor rotamer are enclosed in parenthesis: δ 155.2, (154.7), 142.5, 128.5, (128.5), 128.4, (128.4), 125.9, (125.80), 79.5, (79.5), 69.7, 57.0, 55.9, 54.8, 40.2, (38.9), 36.0, (36.0), 35.0, (34.6), 28.6, (28.4).

Benzyl 2-(3-phenylpropyl)pyrrolidine-1-carboxylate (3l)

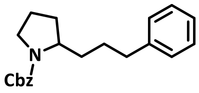

The reaction was performed according to the GP, using Z-Pro-OH (0.3 mmol, 1.5 equiv.; BLD, 99%), $NiCl_2$·glyme (0.2 equiv.; Sigma Aldrich, 99%), dMeObpy (0.2 equiv.; Sigma Aldrich,

99%), nCN$_x$ (50 mg), Na_2CO_3 (2.0 equiv.; Thermo Fisher, 99%) and 1-bromo-3-phenylpropane (1.0 equiv.; Sigma Aldrich, 98%) in 4 mL of MeCN (Sigma Aldrich, 99%). The mixture was purified via silica gel column chromatography (5 to 10% EtOAc/Hexane) to give product **3l** as a colorless oil (44 mg, 68% yield).

R$_f$ (10% EtOAc/Hexane): 0.2.

^{1}H NMR (400 MHz, CDCl$_3$) δ 7.37 – 7.26 (7H, m), 7.20 – 7.14 (3H, m), 5.18 – 5.10 (2H, m), 3.88 (1H, brs), 3.48 – 3.35 (2H, m), 2.67 – 2.56 (2H, m), 1.96 – 1.79 (7H, m), 1.44 – 1.35 (1H, m).

^{13}C NMR (101 MHz, CDCl$_3$), rotameric mixture, resonances for minor rotamer are enclosed in parenthesis**:** δ 155.1, 142.7, 137.2, 128.6, 128.5, 128.4, 128.0, 125.8, 66.8, (66.6), 58.0, (57.3), 46.3, (46.4), 36.1, 34.5, (33.9), 30.8, (30.1), 28.4, (28.1), 24.0, (23.2).

Benzyl 2-(4-ethoxy-4-oxobutyl)pyrrolidine-1-carboxylate (3m)

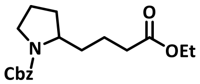

The reaction was performed according to the GP, using Z-Pro-OH (0.3 mmol, 1.5 equiv.; BLD, 99%), $NiCl_2$·glyme (0.2 equiv.; Sigma Aldrich, 99%), dMeObpy (0.2 equiv.; Sigma Aldrich, 99%), nCN$_x$ (50 mg), Na_2CO_3 (2.0 equiv.; Thermo Fisher, 99%) and ethyl 4-bromobutyrate (1.0 equiv.; BLD, 99%) in 4 mL of MeCN (Sigma Aldrich, 99%). The mixture was purified via silica gel column chromatography (5 to 10% EtOAc/Hexane) to give product **3l** as a colorless oil (61 mg, 96% yield).

R$_f$ (20% EtOAc/Hexane): 0.3.

^{1}H NMR (400 MHz, CDCl$_3$) δ 7.38 – 7.26 (5H, m), 5.16 – 5.08 (2H, m), 4.11 (2H, q, *J* = 7.1 Hz), 3.84 (1H, brs), 3.47 – 3.36 (2H, m), 2.33 – 2.24 (2H, m), 1.97 – 1.40 (7H, m), 1.40 – 1.35 (1H, m), 1.24 (3H, t, *J* = 7.4 Hz)

^{13}C NMR (101 MHz, CDCl$_3$), rotameric mixture, resonances for minor rotamer are enclosed in parenthesis: δ 173.7, 155.1, 137.3, 128.6, 128.0, 66.8, (66.6), 60.4, 57.8, (57.1), 46.7, (46.5), 34.3, (33.5), 30.7, (30.0), 24.0, (23.2), 21.8, 14.4.

This data is consistent with literature precedent.[26]

Benzyl 2-(4,4,4-trifluorobutyl)pyrrolidine-1-carboxylate (3n)

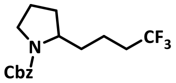

The reaction was performed according to the GP, using Z-Pro-OH (0.3 mmol, 1.5 equiv.; BLD, 99%), $NiCl_2$·glyme (0.2 equiv.; Sigma Aldrich, 99%), dMeObpy (0.2 equiv.; Sigma Aldrich,

99%), nCN_x (50 mg), Na_2CO_3 (2.0 equiv.; Thermo Fisher, 99%) and ethyl 4-bromo-1,1,1-trifluorobutane (1.0 equiv.; BLD, 99%) in 4 mL of MeCN (Sigma Aldrich, 99%). The mixture was purified via silica gel column chromatography (5 to 10% EtOAc/Hexane) to give product **3n** as a colorless oil (62 mg, 98% yield).

R_f (20% EtOAc/Hexane): 0.3.

^{1}H NMR (400 MHz, $CDCl_3$) δ 7.36 – 7.26 (5H, m), 5.17 – 5.09 (2H, m), 3.85 (1H, brs), 3.46 – 3.36 (2H, m), 2.09 – 1.82 (6H, m), 1.69 – 1.63 (2H, m), 1.54 (1H, brs), 1.45 – 1.36 (1H, m).

^{13}C NMR (101 MHz, $CDCl_3$), rotameric mixture, resonances for minor rotamer are enclosed in parenthesis: δ 155.2, 137.1, 128.6, 128.1, 128.0, 67.0, (66.7), 57.6, (56.9), 46.7, (46.5), 33.8 (q, J = 28.9 Hz), 33.3, 30.9, 30.1, 24.0, (23.2), 18.9 (q, J = 3.0 Hz)

^{19}F NMR (376 MHz, $CDCl_3$): δ -66.5 (t, J = 10.9 Hz)

***tert*-butyl-2-(2-(benzyloxy)ethyl)pyrrolidine-1-carboxylate (3o)**

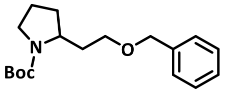

The reaction was performed according to the GP, using Boc-Pro-OH (0.3 mmol, 1.5 equiv.; BLD, 99%), $NiCl_2$·glyme (0.2 equiv.; Sigma Aldrich, 99%), dMeObpy (0.2 equiv.; Sigma Aldrich, 99%), nCN_x (50 mg), Na_2CO_3 (2.0 equiv.; Thermo Fisher, 99%) and ethyl benzyl 2-bromoethyl ether (1.0 equiv.; BLD, 95%) in 4 mL of MeCN (Sigma Aldrich, 99%). The mixture was purified via silica gel column chromatography (5 to 20% EtOAc/Hexane) to give product **3o** as a colorless oil (38 mg, 61% yield).

R_f (20% EtOAc/Hexane): 0.2.

^{1}H NMR (400 MHz, $CDCl_3$) δ 7.36 – 7.31 (5H, m), 5.29 – 5.26 (1H, m), 4.52 (1H, d, J = 11.6 Hz), 4.48 (1H, d, J = 11.4 Hz), 3.87 (1H, br s), 3.52 (1H, br s), 3.38 – 3.30 (2H, m), 2.07 (1H, br s), 1.97 – 1.73 (4H, m), 1.66 – 1.59 (1H, m), 1.45 (9H, s).

^{13}C NMR (101 MHz, $CDCl_3$), rotameric mixture, resonances for minor rotamer are enclosed in parenthesis: δ 154.8, 138.7, 128.5, 127.7, 127.6, 79.2, (79.2), 73.1, 68.4, (68.4), 55.4, (55.4), 46.2, (46.2), 34.9 (34.9), 31.3, 28.7, 23.2.

This data is consistent with literature precedent.[26]

***tert*-butyl 2-(pent-4-en-1-yl)pyrrolidine-1-carboxylate (3p)**

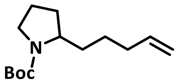

The reaction was performed according to the GP, using Boc-Pro-OH (0.3 mmol, 1.5 equiv.; BLD, 99%), $NiCl_2$·glyme (0.2 equiv.; Sigma Aldrich, 99%), dMeObpy (0.2 equiv.; Sigma Aldrich, 99%), nCN_x (50 mg), Na_2CO_3 (2.0 equiv.; Thermo Fisher, 99%) and 5-bromo-1-pentene (1.0 equiv.; BLD, 98%) in 4 mL of MeCN (Sigma Aldrich, 99%). The mixture was purified via silica gel column chromatography (5 to 10% EtOAc/Hexane) to give product **3p** as a colorless oil (43 mg, 90% yield).

R_f (10% EtOAc/Hexane): 0.3.

^{1}H NMR (400 MHz, $CDCl_3$) δ 5.79 (1H, ddt, *J* = 16.9, 10.2, 6.6 Hz), 5.01 – 4.91 (2H, m), 3.75 – 3.70 (1H, m), 3.38 – 3.25 (2H, m), 2.10 – 1.98 (2H, m), 1.96 – 1.67 (4H, m), 1.65 – 1.58 (1H, m), 1.44 (9H, s), 1.39 – 1.25 (3H, m).

^{13}C NMR (101 MHz, $CDCl_3$), rotameric mixture, resonances for minor rotamer are enclosed in parenthesis: δ 154.8, 138.9, 128.5, 114.5, 79.0, 57.3, (55.4), 46.3, (46.2), (34.2), 33.9, 30.8, (30.0), 28.7, 25.8, 23.9, (23.3).

This data is consistent with literature precedent.[26]

tert-Butyl 2-(3-hydroxypropyl)pyrrolidine-1-carboxylate (3q)

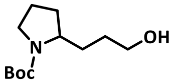

The reaction was performed according to the GP, using Boc-Pro-OH (0.3 mmol, 1.5 equiv.; BLD, 99%), $NiCl_2$·glyme (0.2 equiv.; Sigma Aldrich, 99%), dMeObpy (0.2 equiv.; Sigma Aldrich, 99%), nCN_x (50 mg), Na_2CO_3 (2.0 equiv.; Thermo Fisher, 99%) and 3-bromo-1-propanol (1.0 equiv.; BLD, 95%) in 4 mL of MeCN (Sigma Aldrich, 99%). The mixture was purified via silica gel column chromatography (5 to 30% EtOAc/Hexane) to give product **3q** as a colorless oil (29 mg, 64% yield).

R_f (20% EtOAc/Hexane): 0.15.

^{1}H NMR (400 MHz, $CDCl_3$) δ 3.80 (1H, tt, *J* = 7.8, 3.7 Hz), 3.66 (2H, t, *J* = 6.4 Hz), 3.38 – 3.26 (2H, m), 2.15 (1H, s), 1.96 – 1.73 (4H, m), 1.66 – 1.47 (3H, m), 1.45 (9H, s), 1.41 – 1. 33 (1H, m).

^{13}C NMR (101 MHz, $CDCl_3$) δ 155.0, 79.2, 62.8, 56.9, 46.4, 31.0, 30.6, 29.4, 28.7, 23.6.

This data is consistent with literature precedent.[26]

Benzyl 2-(3-chloropropyl)pyrrolidine-1-carboxylate (3r)

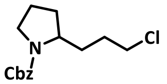

The reaction was performed according to the GP, using Z-Pro-OH (0.3 mmol, 1.5 equiv.; BLD, 99%), $NiCl_2$·glyme (0.2 equiv.; Sigma Aldrich, 99%), dMeObpy (0.2 equiv.; Sigma Aldrich, 99%), nCN_x (50 mg), Na_2CO_3 (2.0 equiv.; Thermo Fisher, 99%) and 1-chloro-3-bromopropane (1.0 equiv.; BLD, 99%) in 4 mL of MeCN (Sigma Aldrich, 99%). The mixture was purified via silica gel column chromatography (5 to 20% EtOAc/Hexane) to give product **3r** as a colorless oil (50 mg, 88% yield).

R_f (10% EtOAc/Hexane): 0.2.

^{1}H NMR (400 MHz, $CDCl_3$) δ 7.38 – 7.30 (5H, m), 5.16 – 5.08 (2H, m), 3.88 (1H, s), 3.58 – 3.36 (4H, m), 1.98 – 1.77 (7H, m), 1.51 (1H, s).

^{13}C NMR (101 MHz, $CDCl_3$), rotameric mixture, resonances for minor rotamer are enclosed in parenthesis: δ 155.2, 137.3, 128.6, 128.6, 128.0. 67.0 (66.7), 57.4, (56.8), 46.5, (45.1), 32.2, (31.8), 31.0, (30.3), 29.7, 25.9, (25.1), 23.9, (23.2).

This data is consistent with literature precedent.[26]

tert-butyl 2-neopentylpyrrolidine-1-carboxylate (3s)

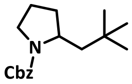

The reaction was performed according to the GP, using Z-Pro-OH (0.3 mmol, 1.5 equiv.; BLD, 99%), $NiCl_2$·glyme (0.2 equiv.; Sigma Aldrich, 99%), dMeObpy (0.2 equiv.; Sigma Aldrich, 99%), nCN_x (50 mg), Na_2CO_3 (2.0 equiv.; Thermo Fisher, 99%) and 1-bromo-2,2-dimethylpropane (1.0 equiv.; BLD, 95%) in 4 mL of MeCN (Sigma Aldrich, 99%). The mixture was purified via silica gel column chromatography (0 to 10% EtOAc/Hexane) to give product **3s** as a colorless oil (20 mg, 37% yield).

R_f (10% EtOAc/Hexane): 0.3.

^{1}H NMR (400 MHz, $CDCl_3$) δ 7.38 – 7.29 (5H, m), 5.17 – 5.08 (2H, m), 3.89 (1H, br d, J = 30.7 Hz), 3.38 (2H, t, J = 7.2 Hz), 2.02 – 1.66 (5H, m), 1.23 (1H, dd, J = 13.6, 10.1 Hz), 0.90 (9H, br d, J = 57.9 Hz).

^{13}C NMR (101 MHz, $CDCl_3$) δ 154.9, 135.2, 1128.5, 128.1, 127.9, 67.0, 54.7, 46.0, 33.4, 32.8, 30.5, 30.1, 24.0

This data is consistent with literature precedent.[26]

Benzyl 2-(oxetan-3-yl)pyrrolidine-1-carboxylate (3t)

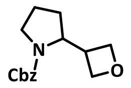

The reaction was performed according to the GP, using Z-Pro-OH (0.3 mmol, 1.5 equiv.; BLD, 99%), $NiCl_2$·glyme (0.2 equiv.; Sigma Aldrich, 99%), dMeObpy (0.2 equiv.; Sigma Aldrich, 99%), nCN_x (50 mg), Na_2CO_3 (2.0 equiv.; Thermo Fisher, 99%) and 3-bromooxetane (1.0 equiv.; BLD, 95%) in 4 mL of MeCN (Sigma Aldrich, 99%). The mixture was purified via silica gel column chromatography (5 to 20% EtOAc/Hexane) to give product **3t** as a colorless oil (41 mg, 79% yield).

R_f (10% EtOAc/Hexane): 0.2.

^{1}H NMR (400 MHz, $CDCl_3$), Rotameric mixture: δ 7.38 – 7.29 (5H, m), 5.14 – 5.06 (2H, m), 4.86 and 4.71 (1H, br s, rotamer), 4.61 – 4.43 (3H, m), 4.19 (1H, br s), 3.62 – 3.53 (1H, m), 3.36 (1H, dt, *J* = 10.8, 6.5 Hz), 3.24 (1H, br s), 2.01 (1H, dq, *J* = 12.4, 7.8 Hz), 1.83 (2H p, *J* = 7.2 Hz), 1.58 (1H, dq, *J* = 11.9, 5.8 Hz).

^{13}C NMR (101 MHz, $CDCl_3$) rotameric mixture, resonances for minor rotamer are enclosed in parenthesis: δ 155.6, 137.0, (136.6), (128.7), 128.6, (128.5), 128.3, (128.2) 128.0, 76.6, 73.8, (68.1), 67.2, (66.9), 59.8, (59.3), (47.4), 47.1, 40.3, (29.3), 28.5, 23.9, (23.2).

This data is consistent with literature precedent.[26]

Benzyl 2-(tetrahydropyran-4-yl)pyrrolidine-1-carboxylate) (3u)

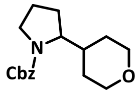

The reaction was performed according to the GP, using Z-Pro-OH (0.3 mmol, 1.5 equiv.; BLD, 99%), $NiCl_2$·glyme (0.2 equiv.; Sigma Aldrich, 99%), dMeObpy (0.2 equiv.; Sigma Aldrich, 99%), nCN_x (50 mg), Na_2CO_3 (2.0 equiv.; Thermo Fisher, 99%) and 4-bromotetrahydropyran (1.0 equiv.; BLD, 95%) in 4 mL of MeCN (Sigma Aldrich, 99%). The mixture was purified via silica gel column chromatography (5 to 20% EtOAc/Hexane) to give product **3u** as a colorless oil (43 mg, 75% yield).

R_f (10% EtOAc/Hexane): 0.2.

^{1}H NMR (400 MHz, $CDCl_3$) δ 7.38 – 7.25 (5H, m), 5.18 – 5.08 (2H, m), 3.98 (2H, br d, *J* = 11.0 Hz), 3.81 (1H, br d, *J* = 25.9 Hz), 3.58 (1H, br d, *J* = 26.4 Hz), 3.33 – 3.24 (3H, m), 2.08 (1H, br s), 1.86 – 1.78 (4H, m), 1.48 – 1.34 (4H, m).

^{13}C NMR (101 MHz, $CDCl_3$), rotameric mixture, resonances for minor rotamer are enclosed in parenthesis: δ 155.6, 137.2, 128.6, 128.0, 127.9. 68.3 (68.1), 66.8, 62.0, (61.4), 47.0, 38.9, (38.1), 30.1, 28.2, (27.9), 27.4, (26.5), 24.5, (23.6).

This data is consistent with literature precedent.[26]

Benzyl 2-cyclopentylpyrrolidine-1-carboxylate (3v)

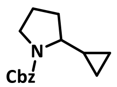

The reaction was performed according to the GP, using Z-Pro-OH (0.3 mmol, 1.5 equiv.; BLD, 99%), $NiCl_2$·glyme (0.2 equiv.; Sigma Aldrich, 99%), dMeObpy (0.2 equiv.; Sigma Aldrich, 99%), nCN_x (50 mg), Na_2CO_3 (2.0 equiv.; Thermo Fisher, 99%) and bromocyclopropane (1.0 equiv.; BLD, 97%) in 4 mL of MeCN (Sigma Aldrich, 99%). The mixture was purified via silica gel column chromatography (5 to 20% EtOAc/Hexane) to give product **3v** as a colorless oil (33 mg, 67% yield).

R_f (10% EtOAc/Hexane): 0.3.

^{1}H NMR (400 MHz, $CDCl_3$) δ 7.39 – 7.28 (5H, m), 5.13 (2H, q, *J* = 11.1), 3.45 (3H, dd, *J* = 8.3, 4.1), 2.00 – 1.75 (4H, m), 0.89 – 0.87 (1H, m), 0.64 – 0.33 (3H, m), 0.12 (1H, s).

^{13}C NMR (101 MHz, $CDCl_3$) δ 155.6, 137.2, 128.6, 128.1, 128.0, 66.8, 61.7, 46.9, 31.0, 24.0, 15.9, 4.6, 1.9.

This data is consistent with literature precedent.[26]

Benzyl 2-cyclopentylpyrrolidine-1-carboxylate (3w)

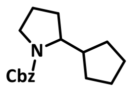

The reaction was performed according to the GP, using Z-Pro-OH (0.3 mmol, 1.5 equiv.; BLD, 99%), $NiCl_2$·glyme (0.2 equiv.; Sigma Aldrich, 99%), dMeObpy (0.2 equiv.; Sigma Aldrich, 99%), nCN_x (50 mg), Na_2CO_3 (2.0 equiv.; Thermo Fisher, 99%) and bromocyclopentane (1.0 equiv. BLD, 97%) in 4 mL of MeCN (Sigma Aldrich, 99%). The mixture was purified via silica gel column chromatography (5 to 20% EtOAc/Hexane) to give product **3w** as a colorless oil (33 mg, 60% yield).

R_f (10% EtOAc/Hexane): 0.3.

^{1}H NMR (400 MHz, $CDCl_3$) δ 7.38 – 7.25 (5H, m), 5.17 – 5.09 (2H, m), 3.93 (1H, br s), 3.53 (1H, br s), 3.34 (1H, ddd, *J* = 11.4, 8.0, 4.1 Hz), 2.11 (1H, br d, *J* = 27.2 Hz), 1.93 – 1.70 (3H, m), 1.62 – 1.50 (8H, m), 1.20 (1H, br s).

^{13}C NMR (101 MHz, $CDCl_3$) δ 155.7, 137.4, 128.5, 128.1, 127.9, 66.7, 61.5, 46.6, 44.3, 30.1, 28.9, 25.9, 25.4, 25.2.

This data is consistent with literature precedent.[26]

***tert*-Butyl 2-cyclohexylpyrrolidine-1-carboxylate (3x)**

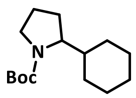

The reaction was performed according to the GP, using Boc-Pro-OH (0.3 mmol, 1.5 equiv.; BLD, 99%), $NiCl_2$·glyme (0.2 equiv.; Sigma Aldrich, 99%), dMeObpy (0.2 equiv.; Sigma Aldrich, 99%), nCN_x (50 mg), Na_2CO_3 (2.0 equiv.; Thermo Fisher, 99%) and bromocyclohexane (1.0 equiv.; BLD, 99%) in 4 mL of MeCN (Sigma Aldrich, 99%). The mixture was purified via silica gel column chromatography (5 to 20% EtOAc/Hexane) to give product **3x** as a colorless oil (30 mg, 59% yield).

R_f (10% EtOAc/Hexane): 0.3.

^{1}H NMR (400 MHz, $CDCl_3$) δ 3.67 (1H, br d, J = 29.5 Hz), 3.46 (1H, br d, J = 27.1 Hz), 3.20 (1H, dt, J = 9.7, 5.9 Hz), 1.83 – 1.60 (9H, m), 1.46 (9H, s), 1.30 – 0.84 (6H, m).

^{13}C NMR (101 MHz, $CDCl_3$) δ 155.3, 62.0, 51.8, 46.6, 41.2, 30.3, 28.7, 28.2, 26.8, 26.5, 24.4.

This data is consistent with literature precedent.[26]

***tert* -butyl (1-(3,4-dimethoxyphenyl)-5-phenylpentan-2-yl)carbamate (3y)**

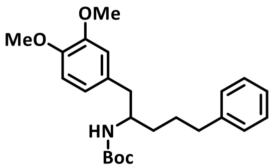

The reaction was performed according to the GP, using Boc-3,4-dimethoxy-Phe-OH (0.3 mmol, 1.5 equiv.; BLD, 99%), $NiCl_2$·glyme (0.2 equiv.; Sigma Aldrich, 99%), dMeObpy (0.2 equiv.; Sigma Aldrich, 99%), nCN_x (50 mg), Na_2CO_3 (2.0 equiv.; Thermo Fisher, 99%) and 1-bromo-3-phenylpropane (1.0 equiv.; Sigma Aldrich, 98%) in 4 mL of MeCN (Sigma Aldrich, 99%). The mixture was purified via silica gel column chromatography (5 to 20% EtOAc/Hexane) to give product **3y** as a colorless oil (57 mg, 71% yield).

R_f (20% EtOAc/Hexane): 0.1.

^{1}H NMR (400 MHz, $CDCl_3$) δ 7.39 – 7.35 (2H, m), 7.30 – 7.24 (3H, m), 6.90 (1H, d, J = 8.1 Hz), 6.80 – 6.78 (2H, m), 4.43 (1H, br s), 3.97 (3H, s), 3.97 (3H, s), 2.81 – 2.63 (4H, m). 1.86 – 1.73 (2H, m) 1.69 – 1.35 (12H, m)

^{13}C NMR (101 MHz, $CDCl_3$): δ 155.6, 148.8, 147.6, 142.3, 130.9, 128.4, 128.3, 125.8, 121.6, 112.7, 111.3, 79.0, 56.0, 55.9, 51.4, 41.0, 35.6, 33.8, 28.5, 27.9.

NMR spectra.

tert-Butyl 2-(3-phenylpropyl)pyrrolidine-1-carboxylate (3a)

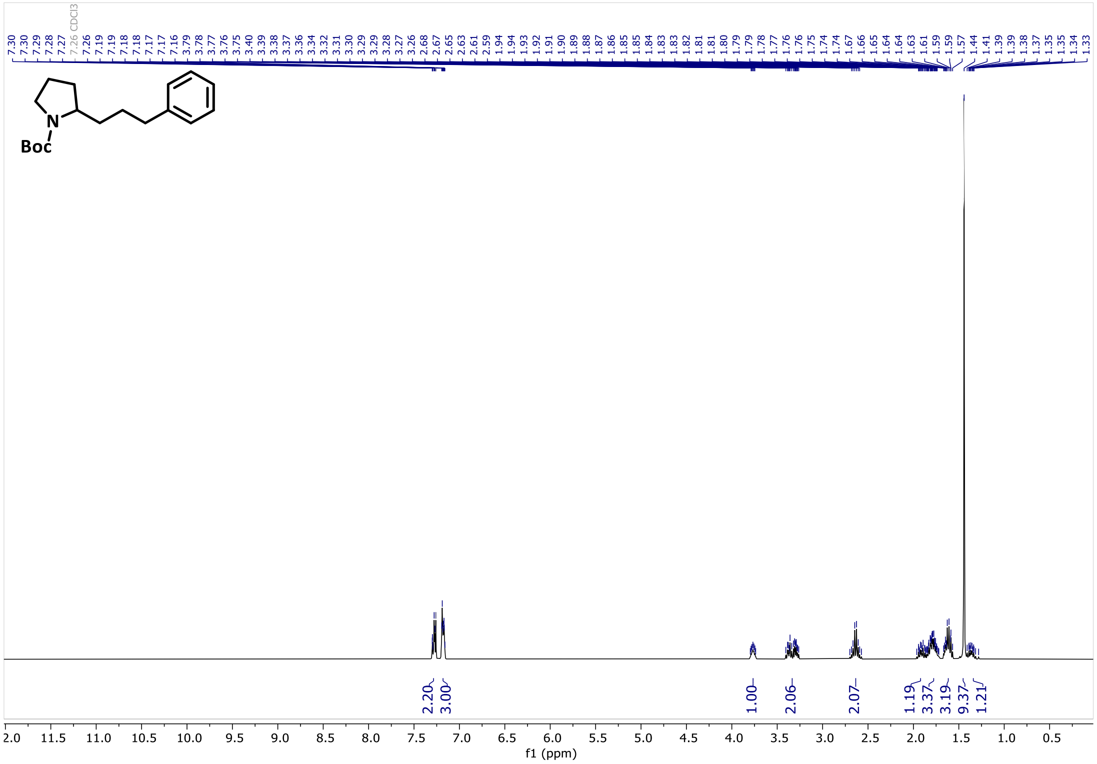

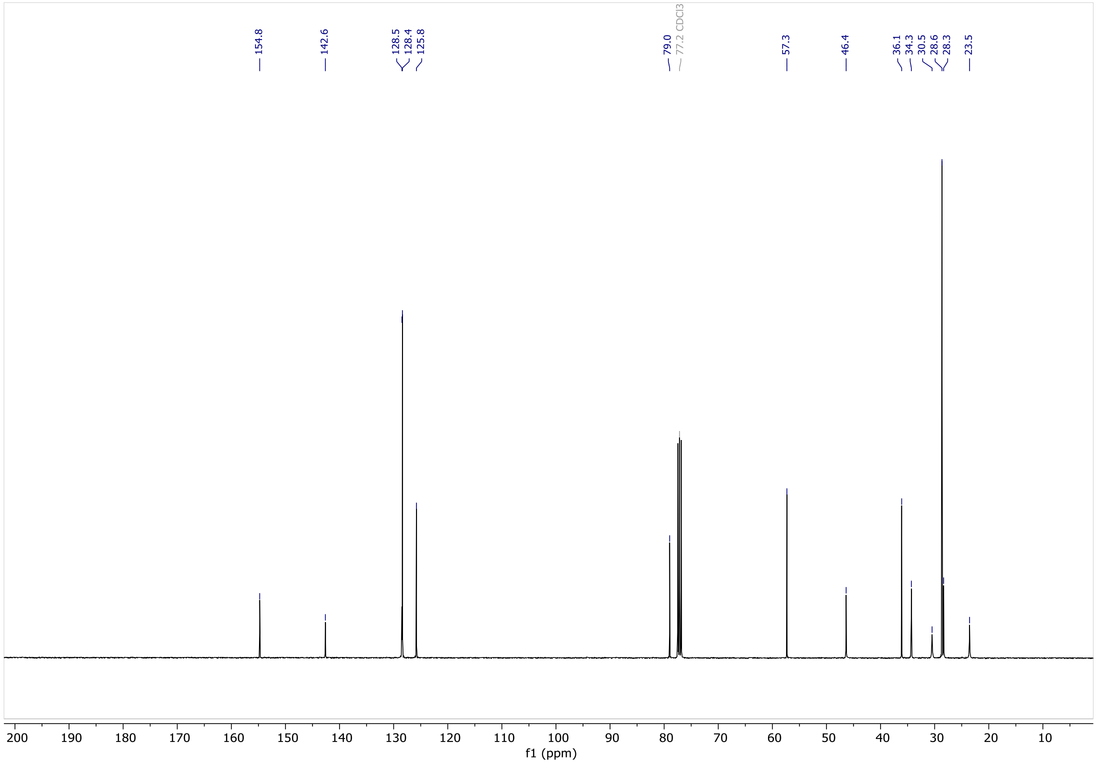

tert-Butyl 2-(3-phenylpropyl)azetidine-1-carboxylate (3b)

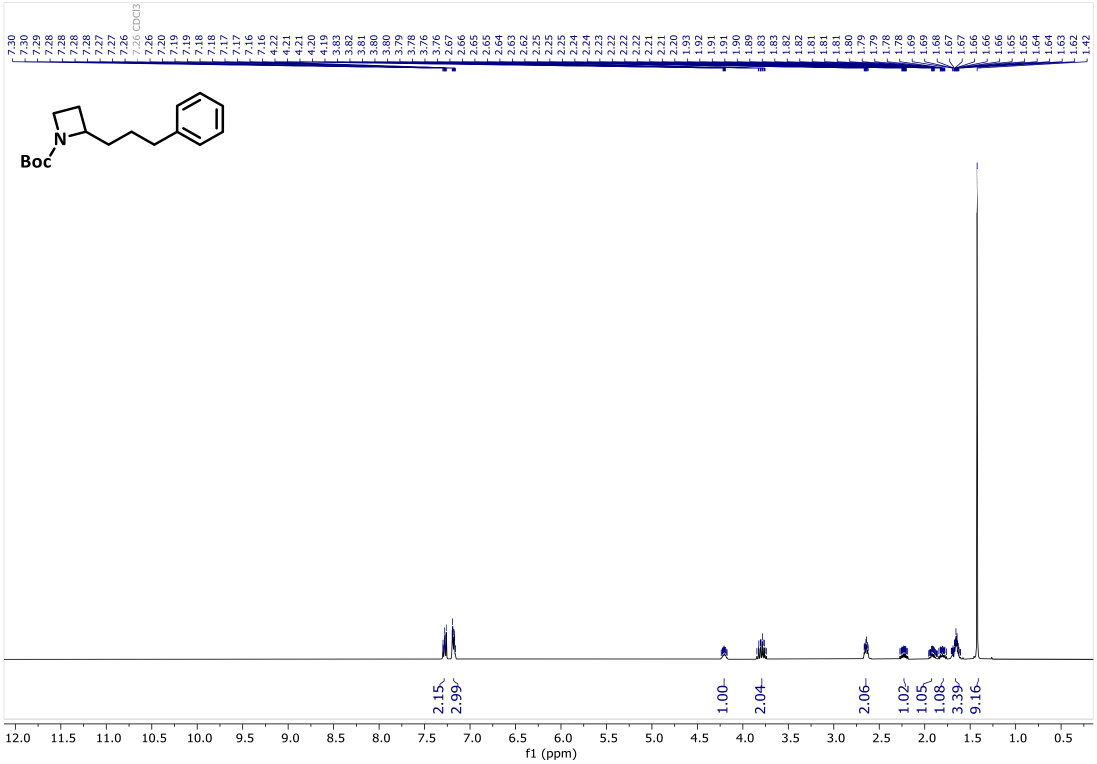

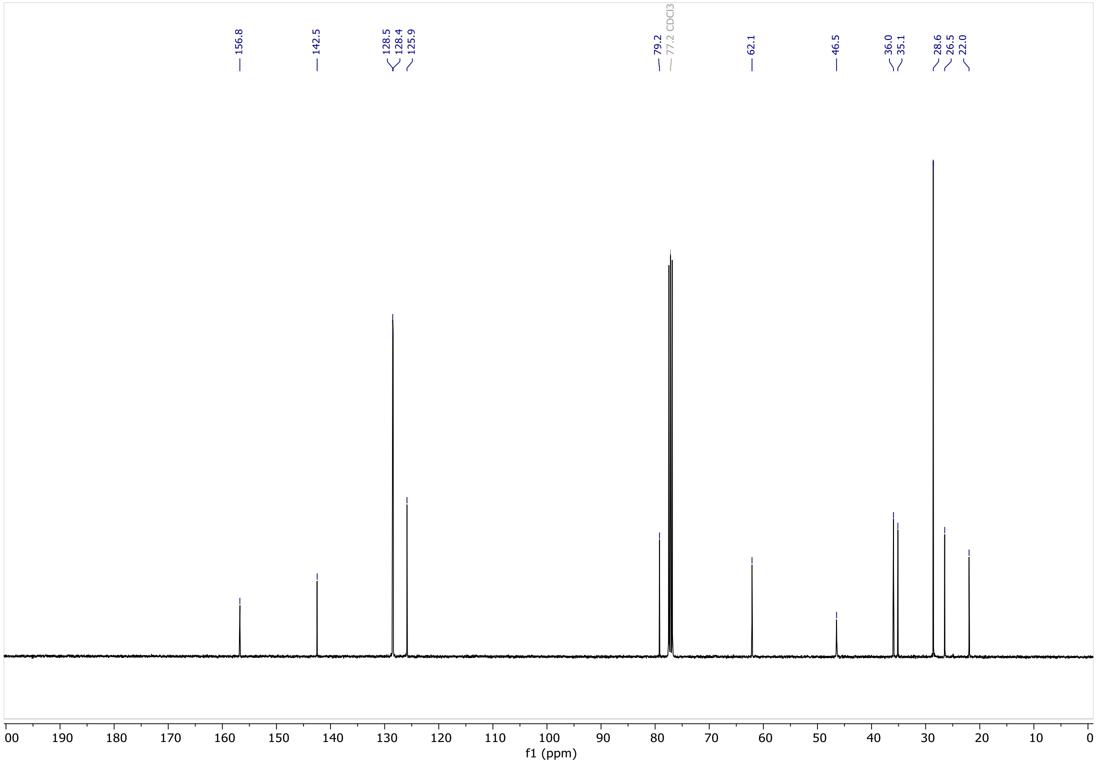

tert-Butyl 2-(3-phenylpropyl)piperidine-1-carboxylate (3c)

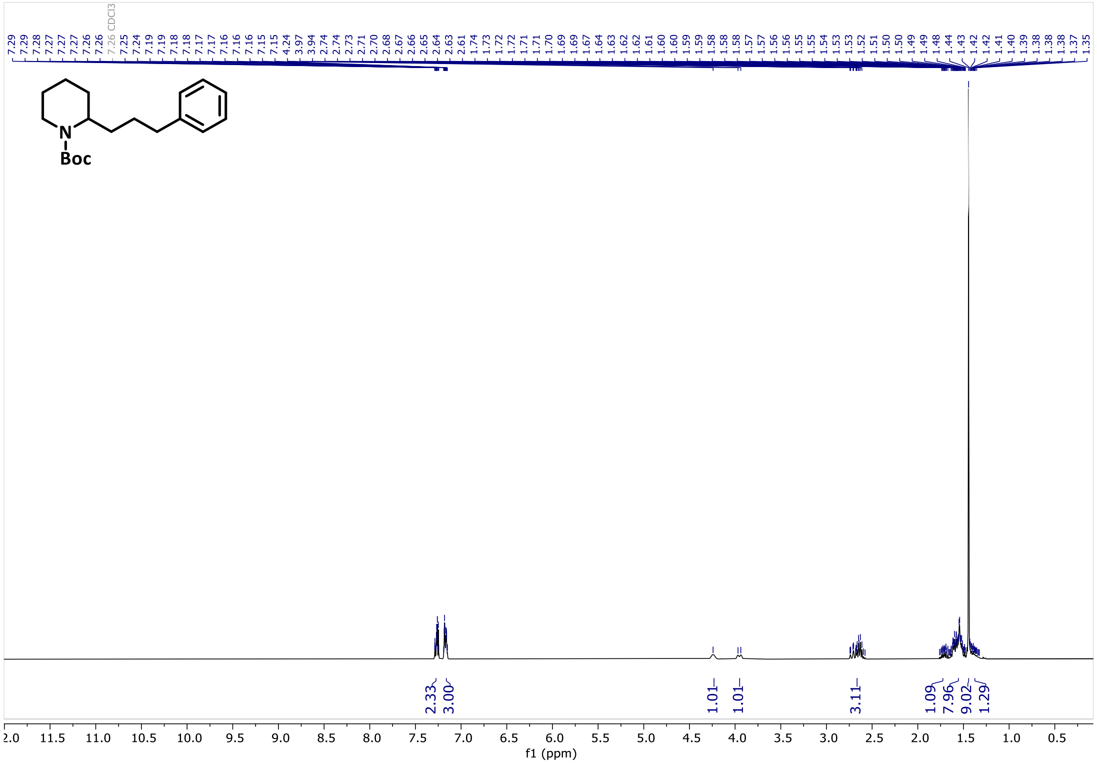

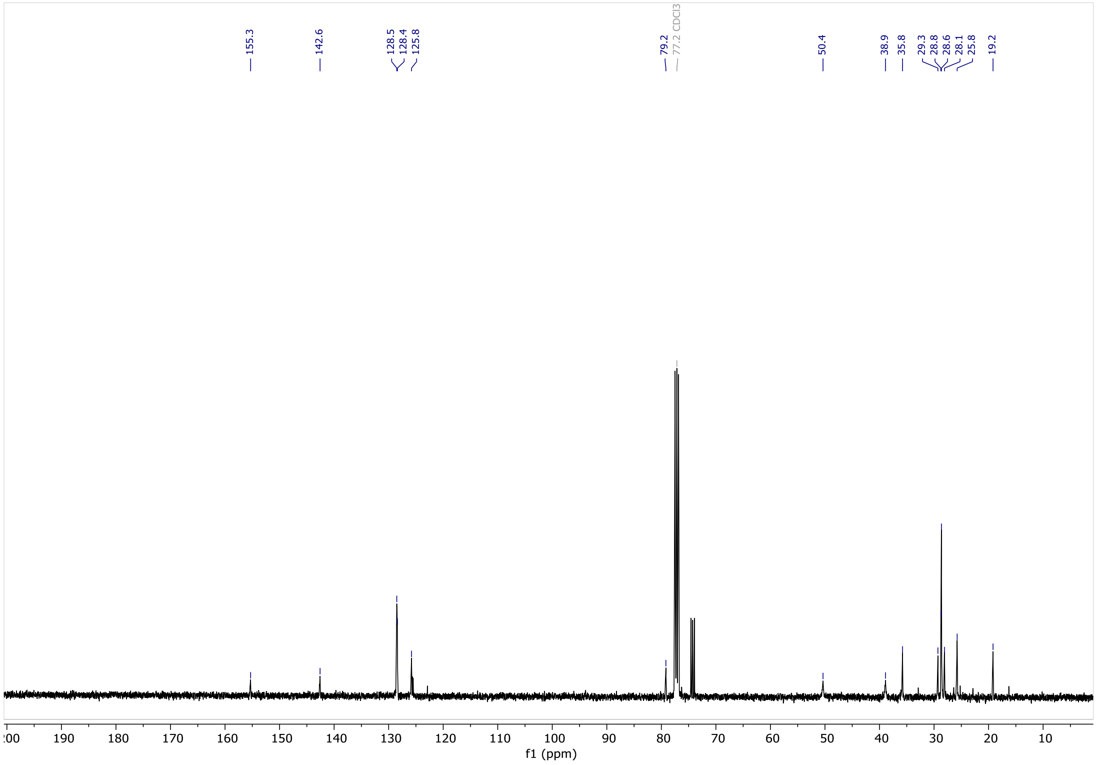

tert-Butyl (5-phenylpentan-2-yl)carbamate (3d)

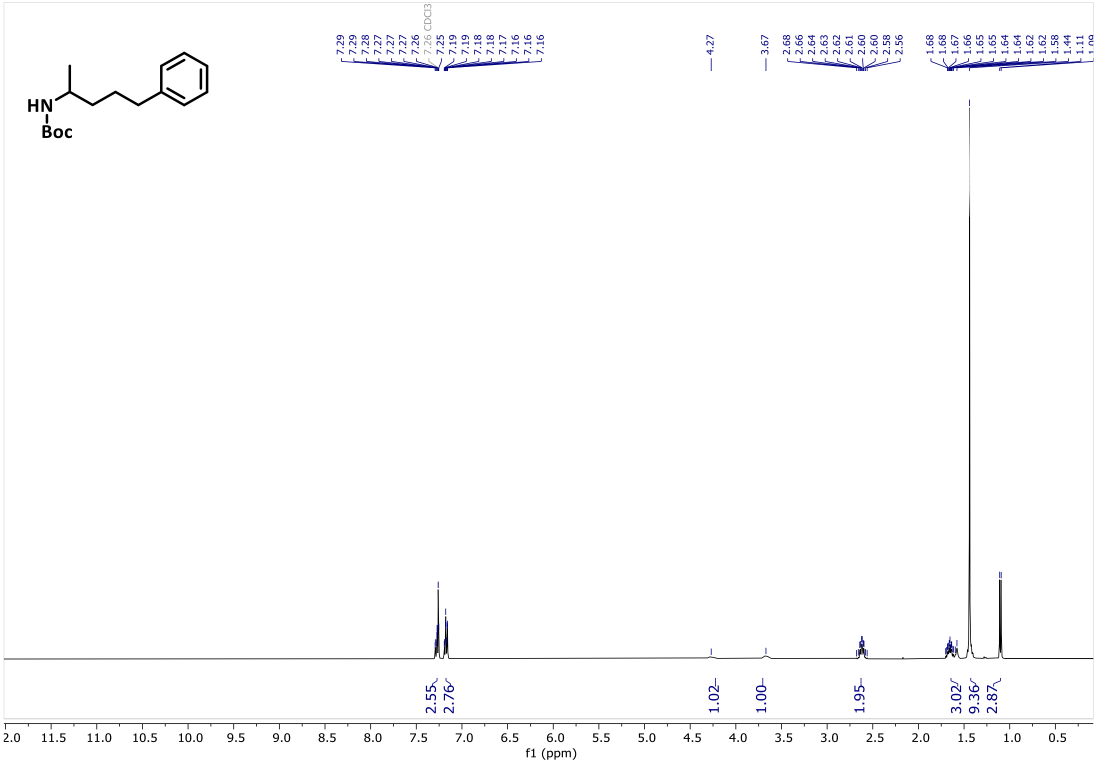

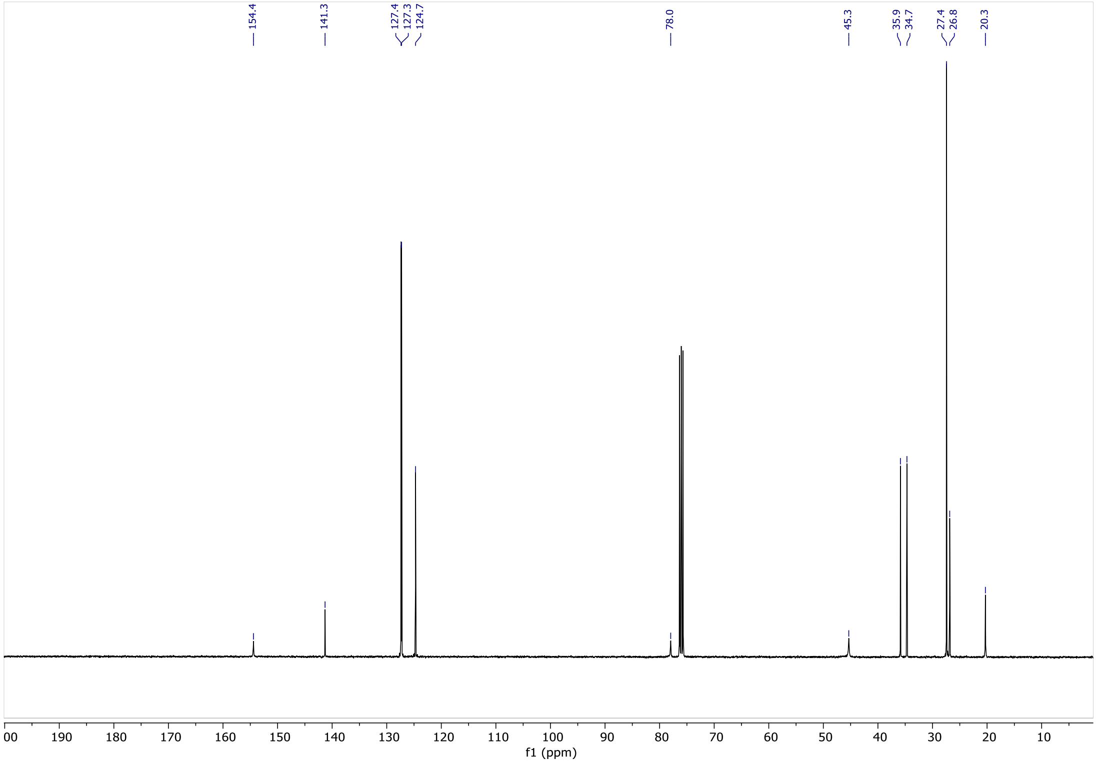

***tert*-Butyl (2-methyl-6-phenylhexan-3-yl)carbamate (3e)**

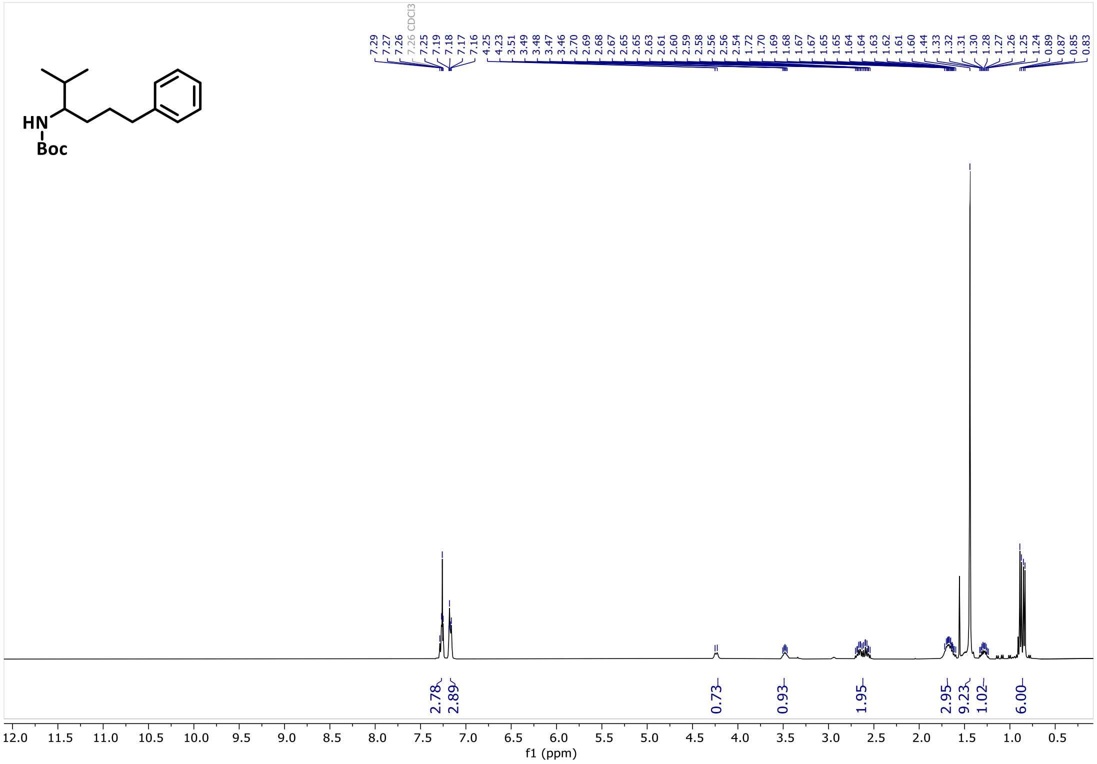

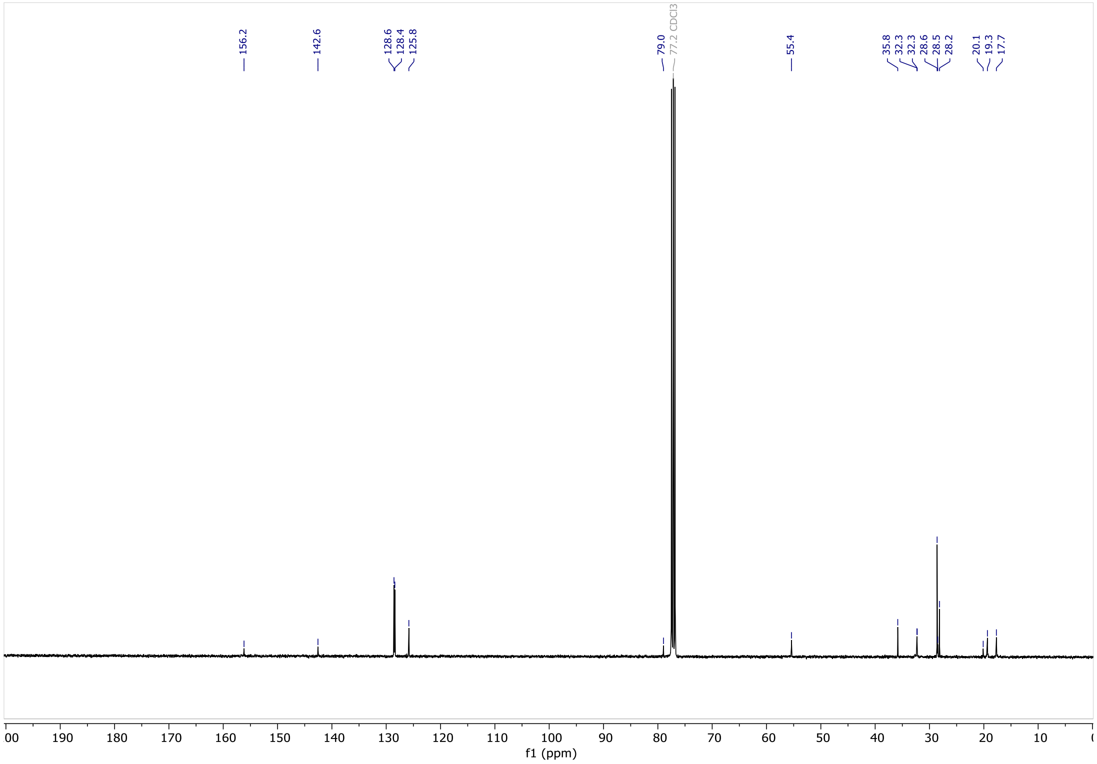

***tert*-butyl (1,5-diphenylpentan-2-yl)carbamate (3f)**

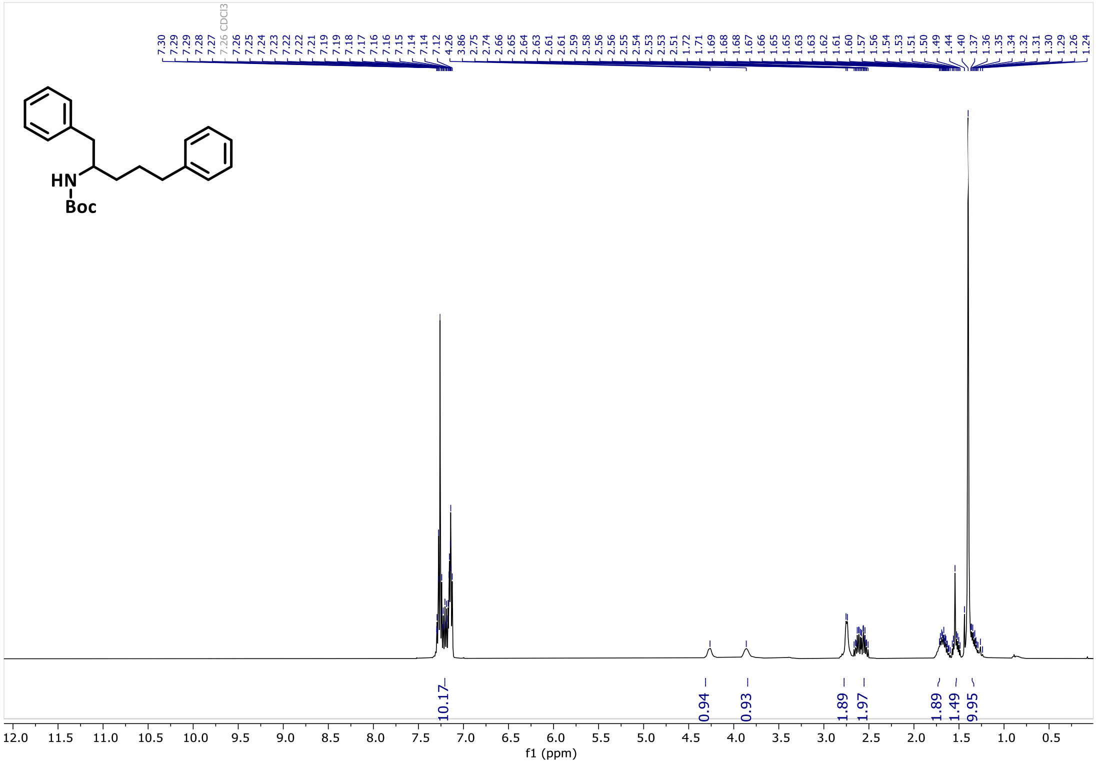

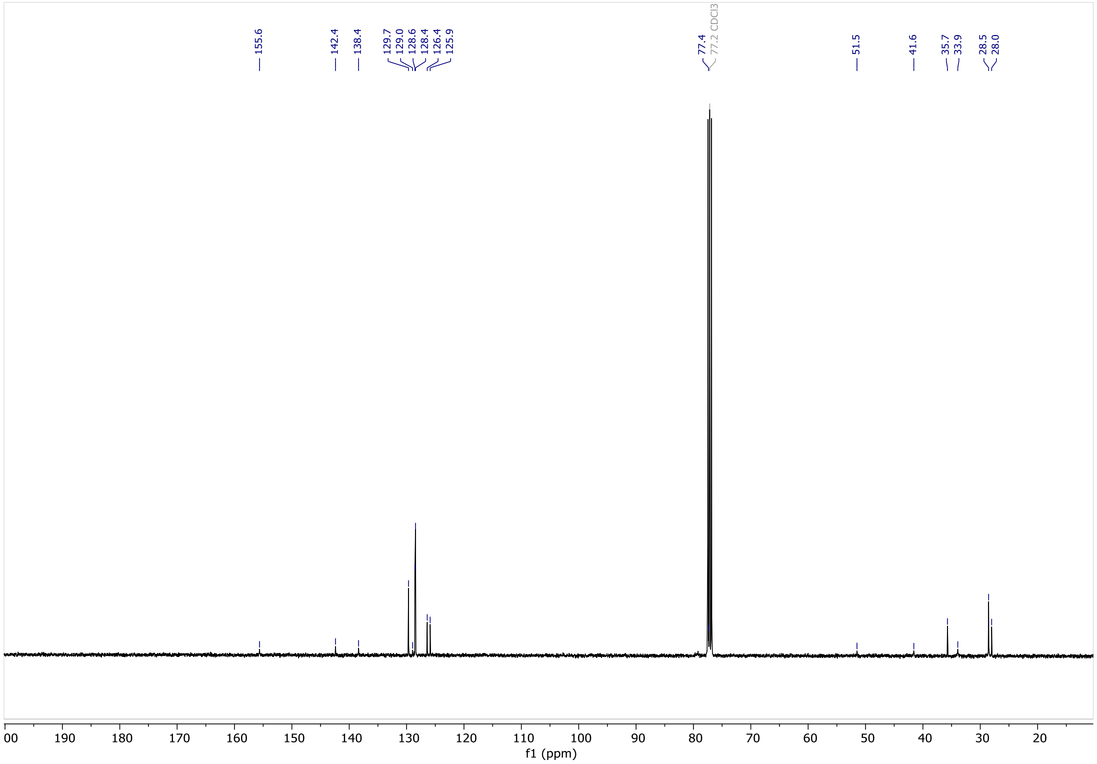

Benzyl *tert*-butyl (8-phenyloctane-1,5-diyl)dicarbamate (3g)

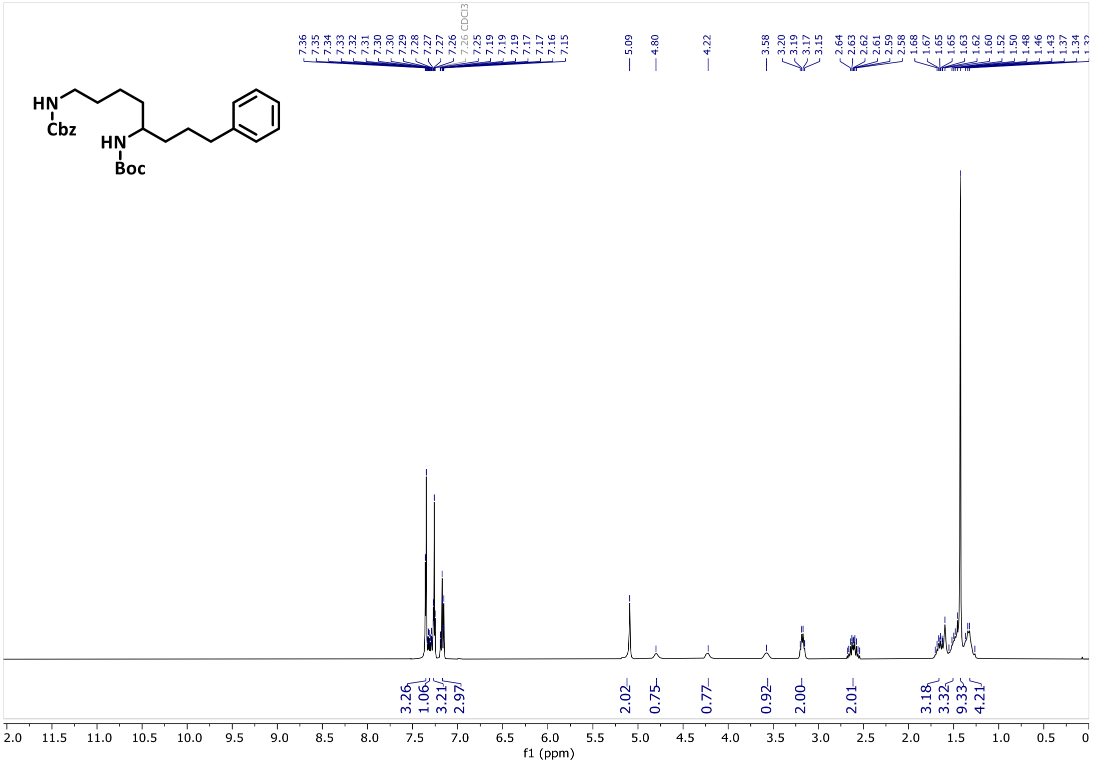

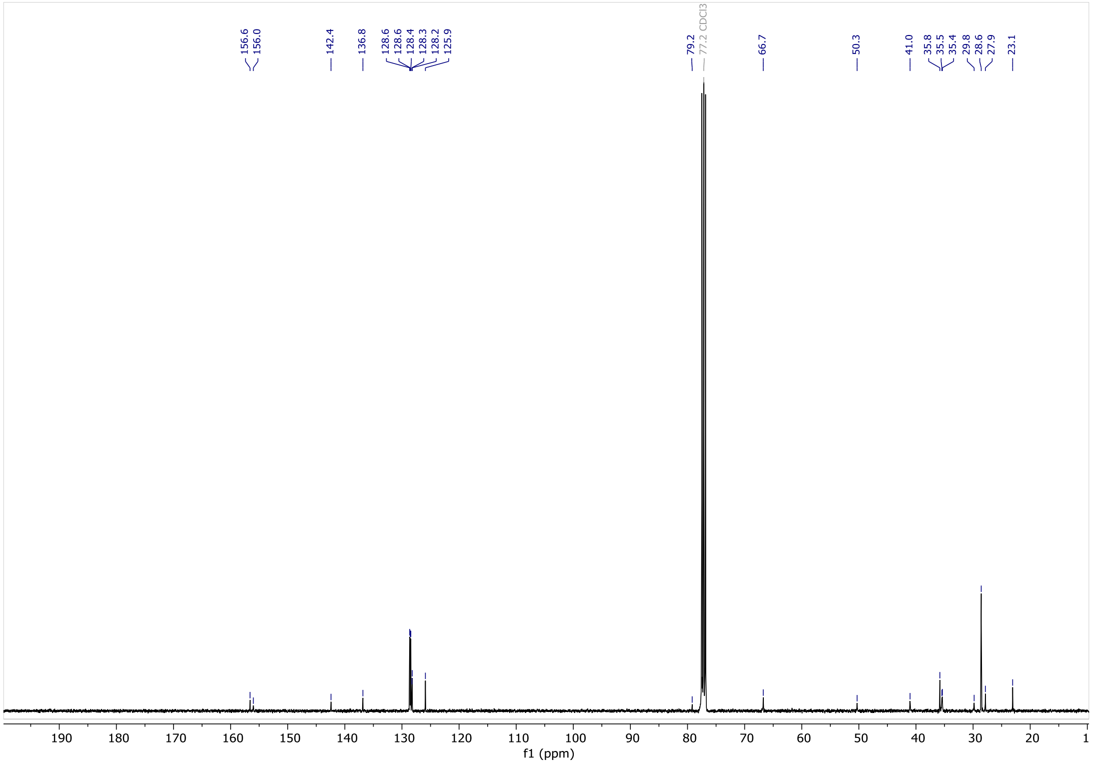

***tert*-Butyl 2-(3-phenylpropyl)octahydro-1H-indole-1-carboxylate (3h)**

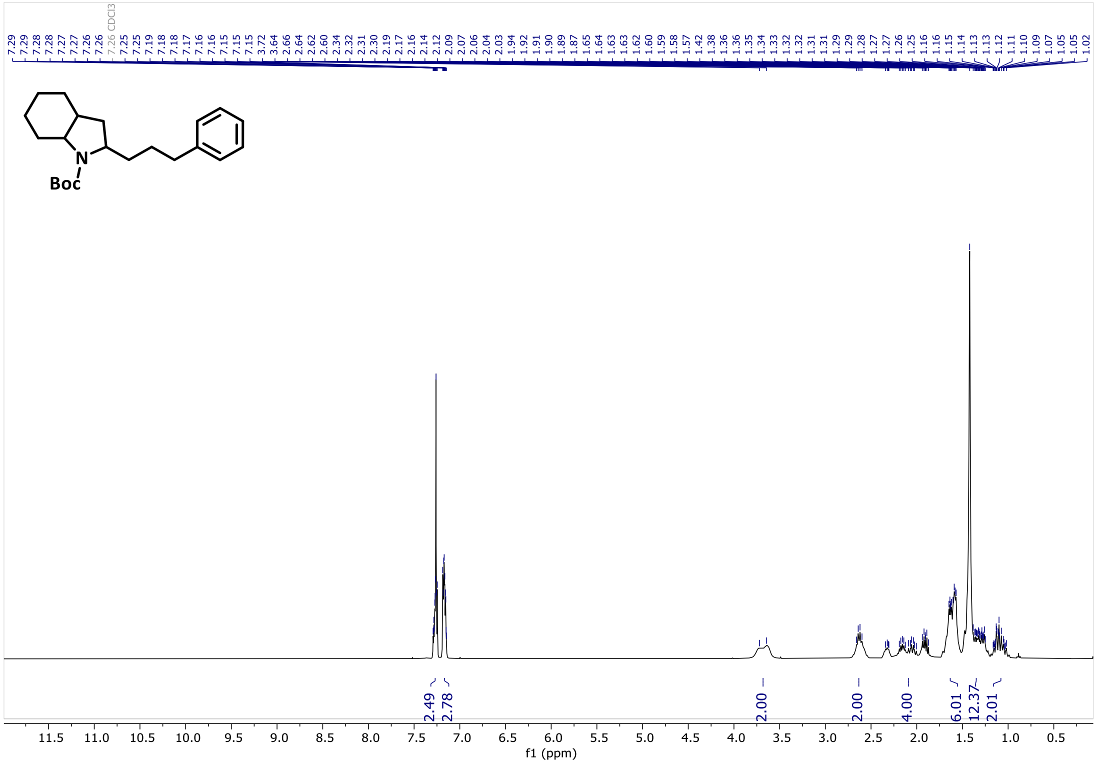

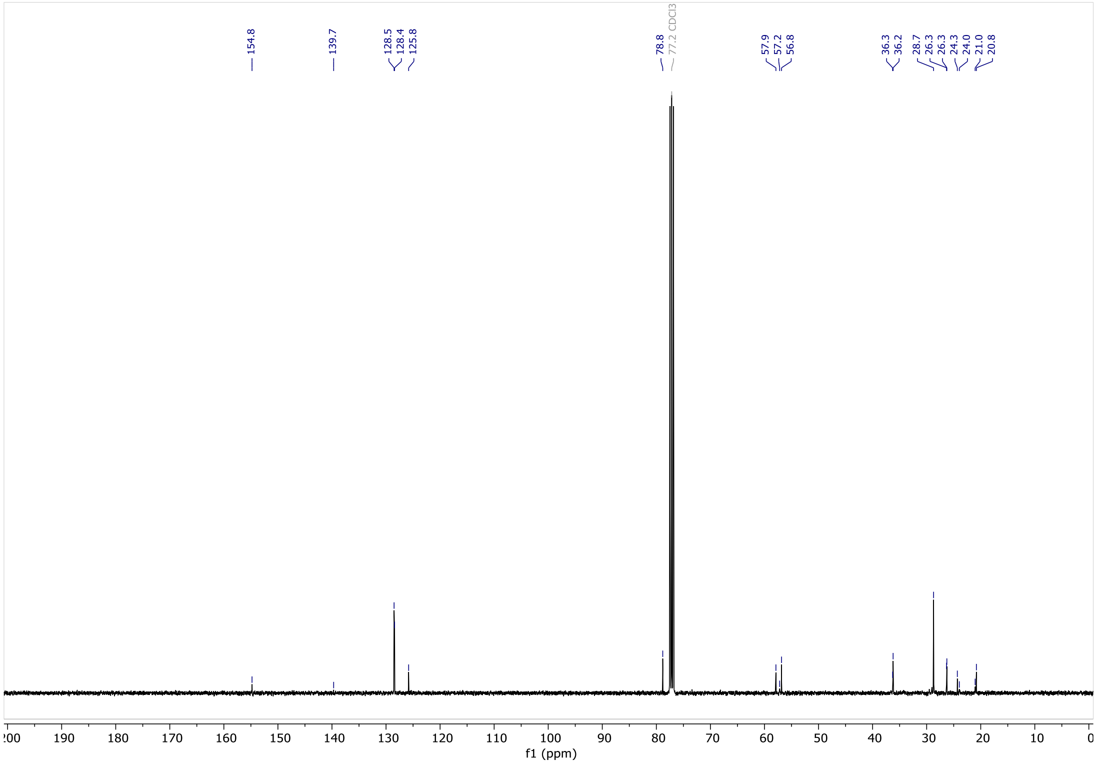

***tert*-Butyl 3-(3-phenylpropyl)-2-azabicyclo[2.2.1]heptane-3-carboxylate (3i)**

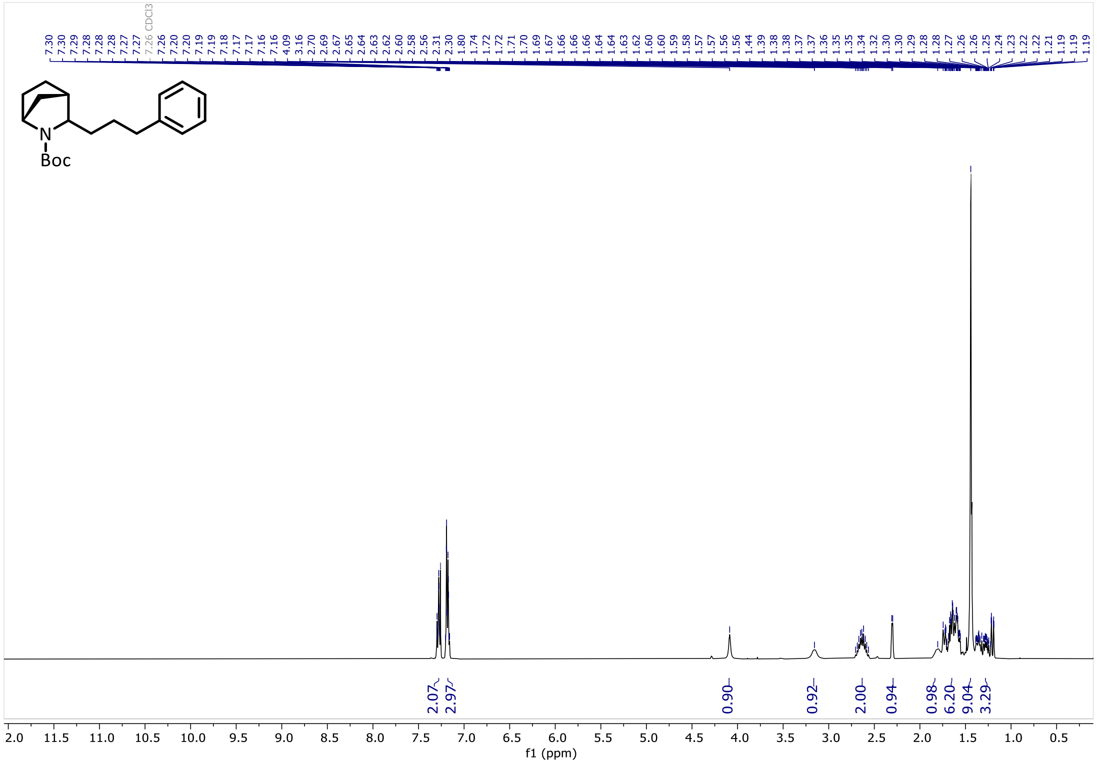

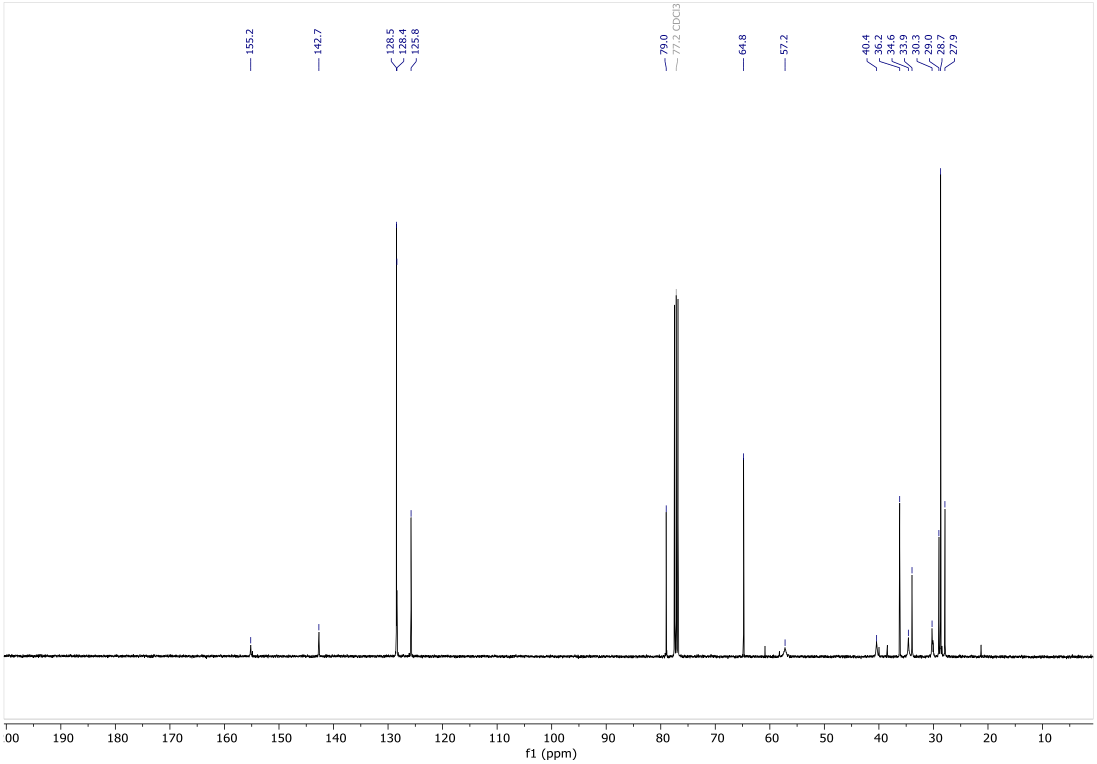

tert-Butyl 2-(3-phenylpropyl)-3-fluoropyrrolidine-1-carboxylate (3j)

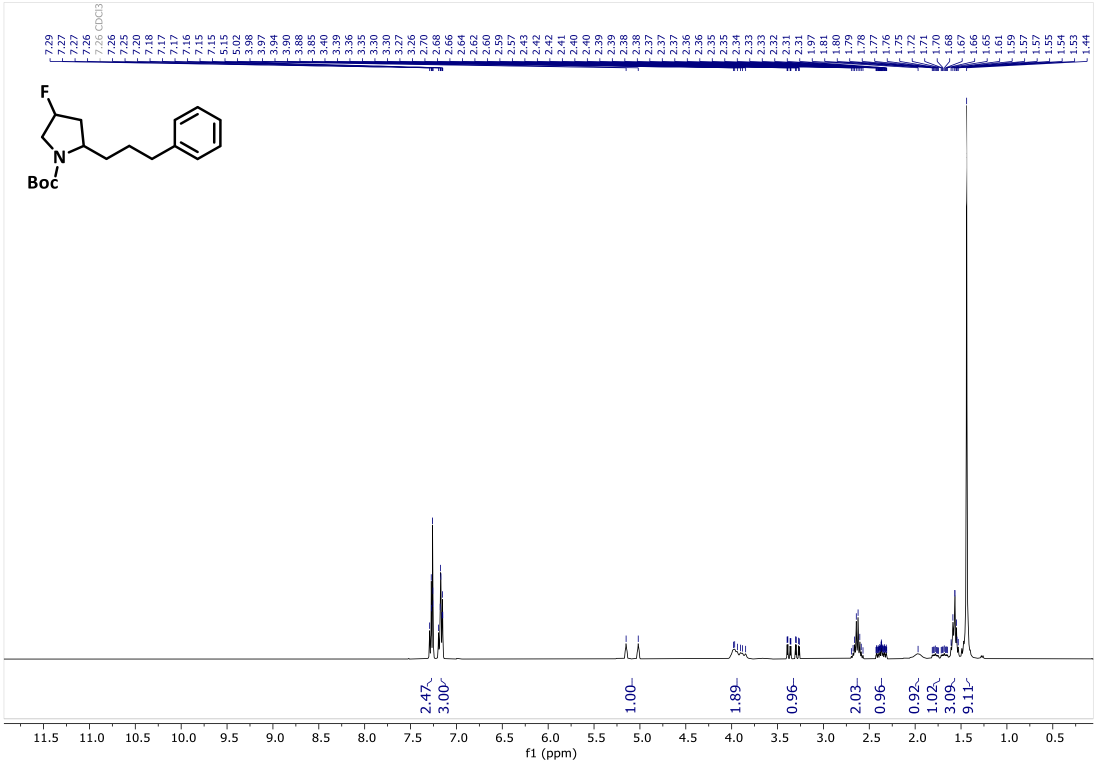

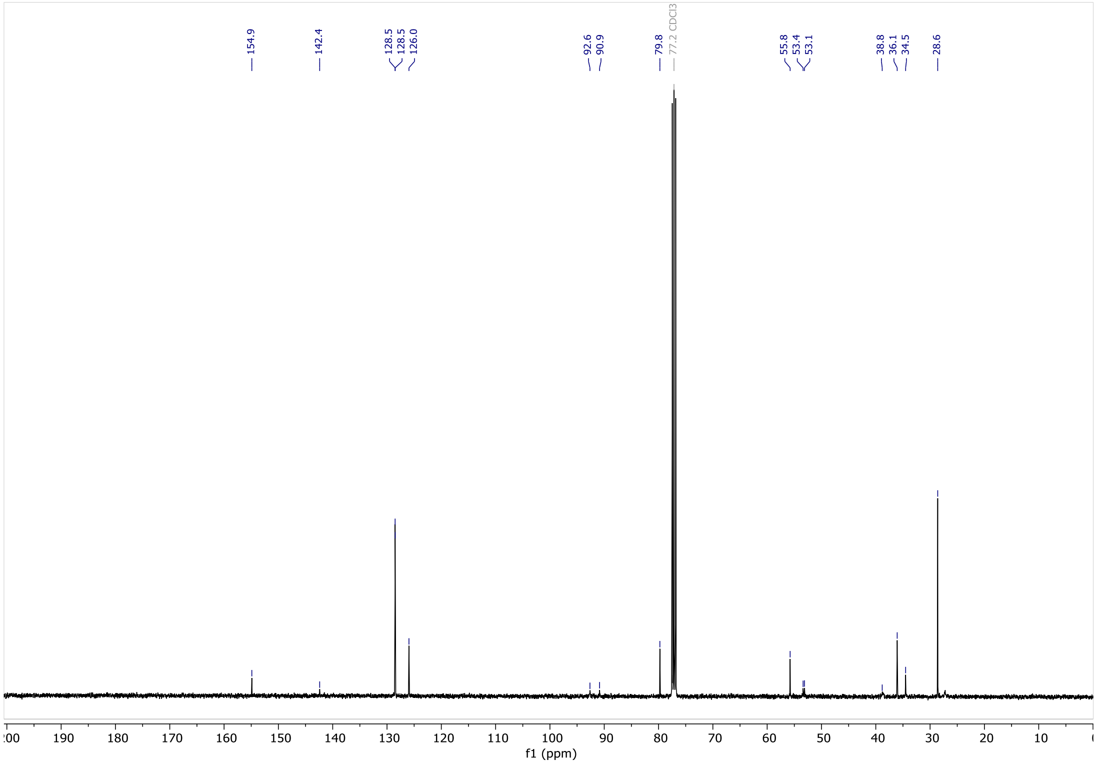

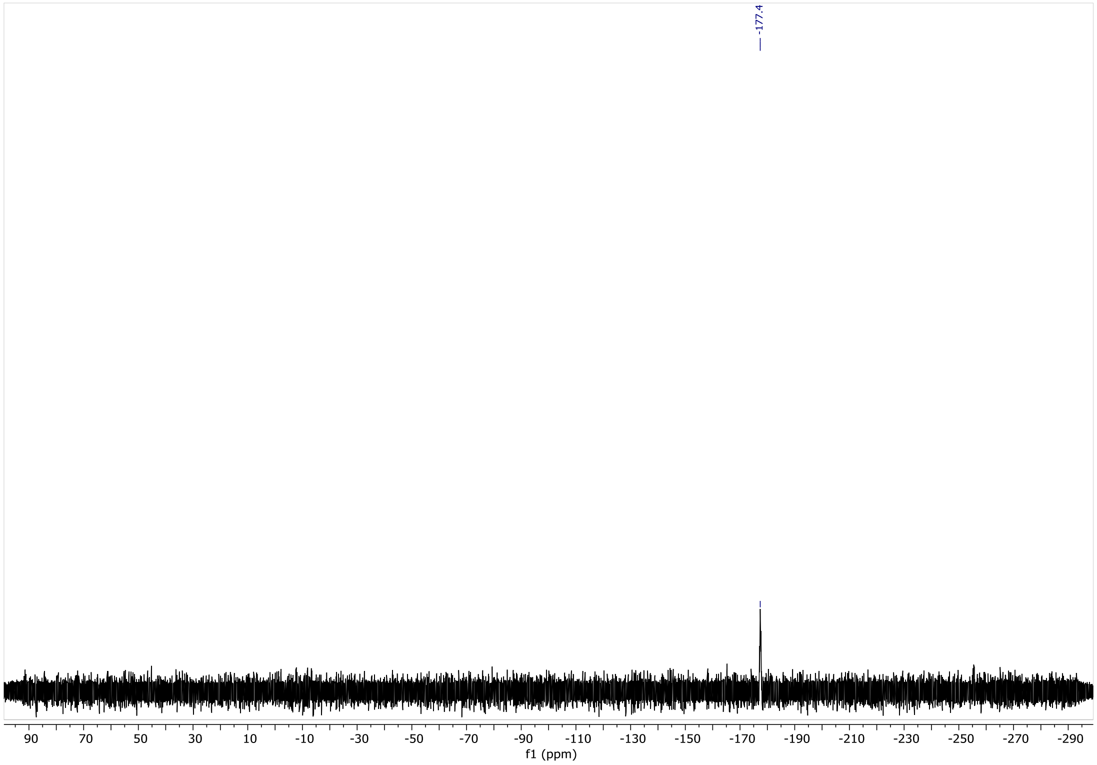
-177.4
90
70
50
30
10
-10
-30
-50
-70
-90
-110
-130
-150
-170
-190
-210
-230
-250
-270
-290
f1 (ppm)

tert-Butyl 2-(3-phenylpropyl)-3-hydroxypyrrolidine-1-carboxylate (3k)

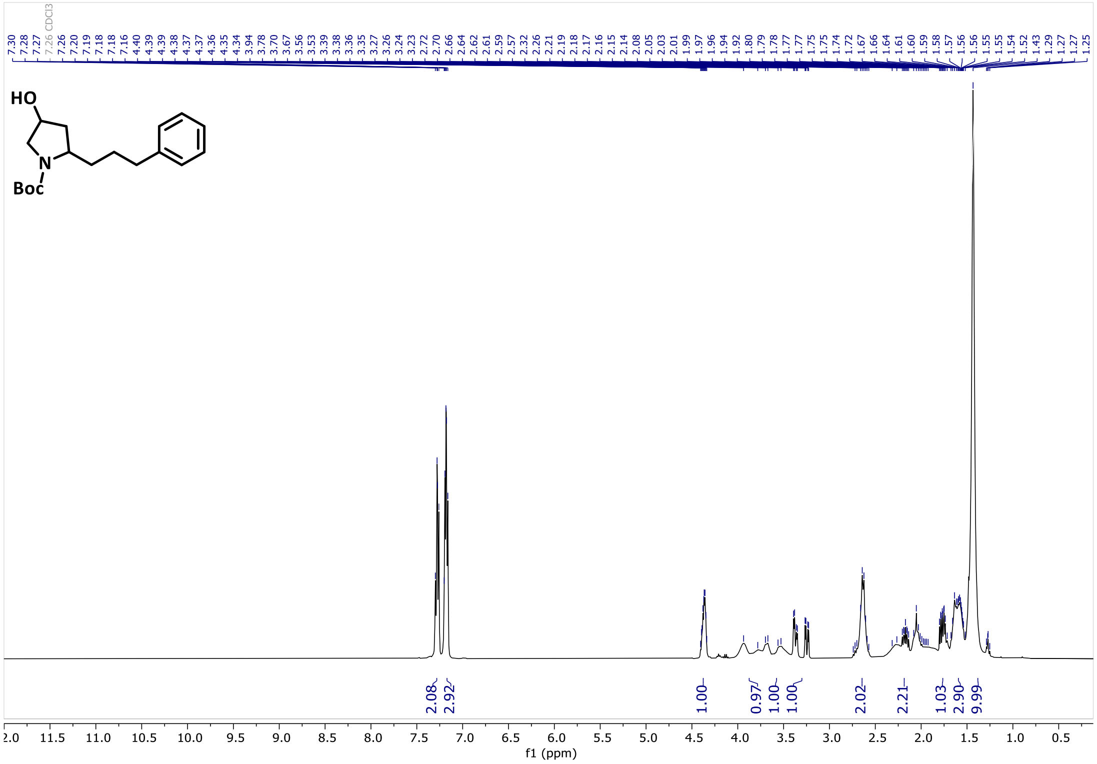

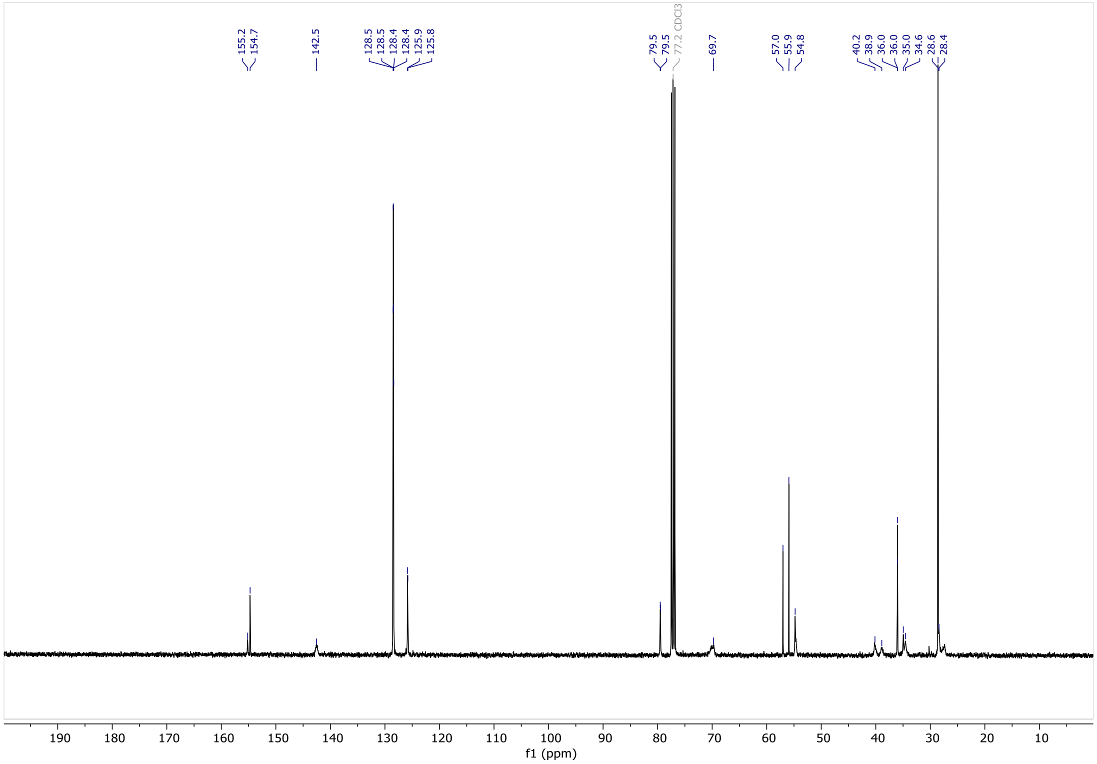

Benzyl 2-(3-phenylpropyl)pyrrolidine-1-carboxylate (3l)

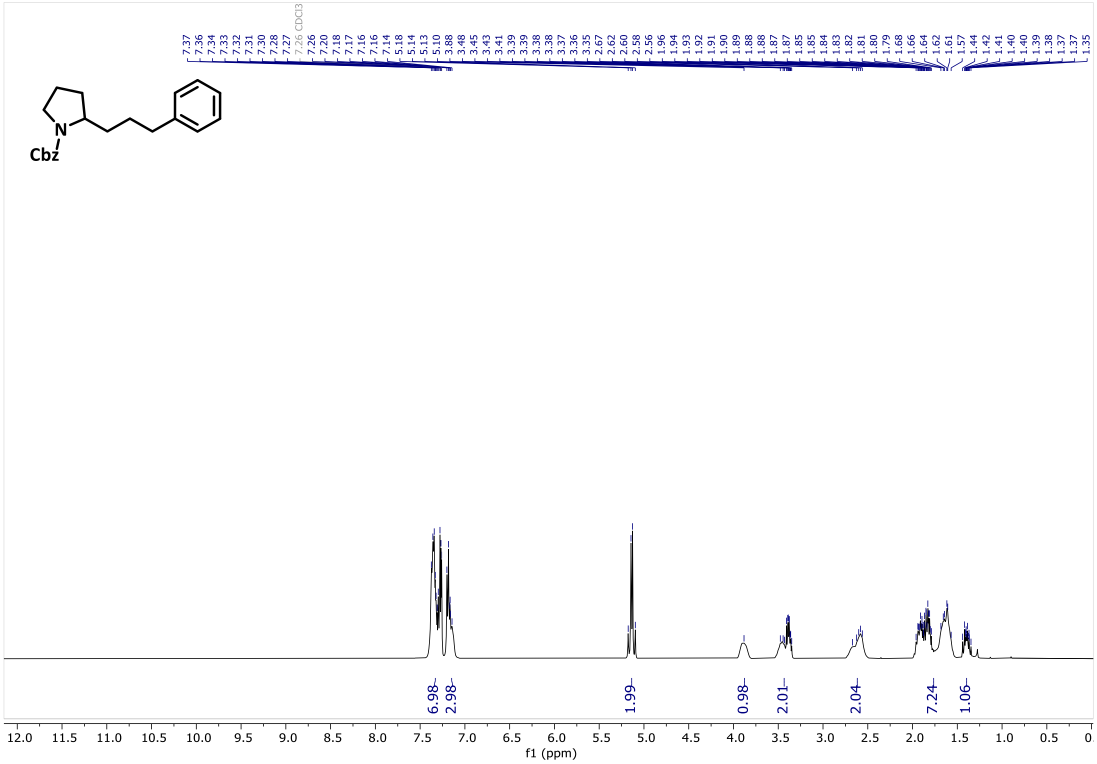

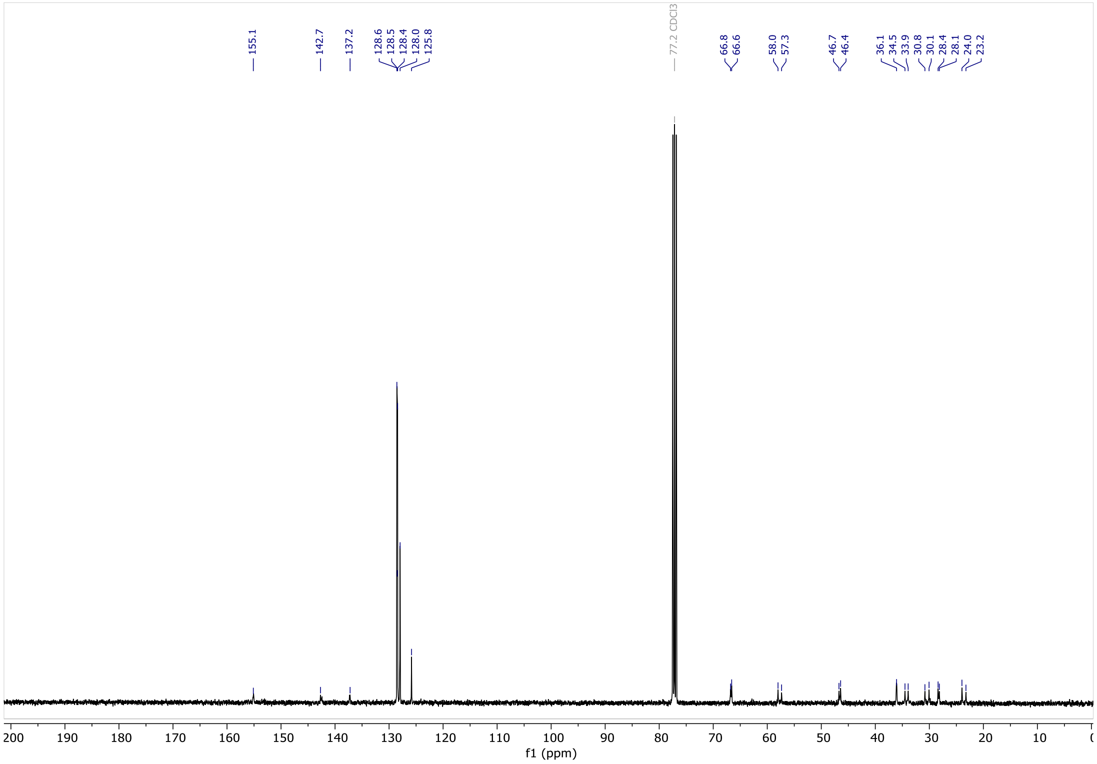

Benzyl 2-(4-ethoxy-4-oxobutyl)pyrrolidine-1-carboxylate (3m)

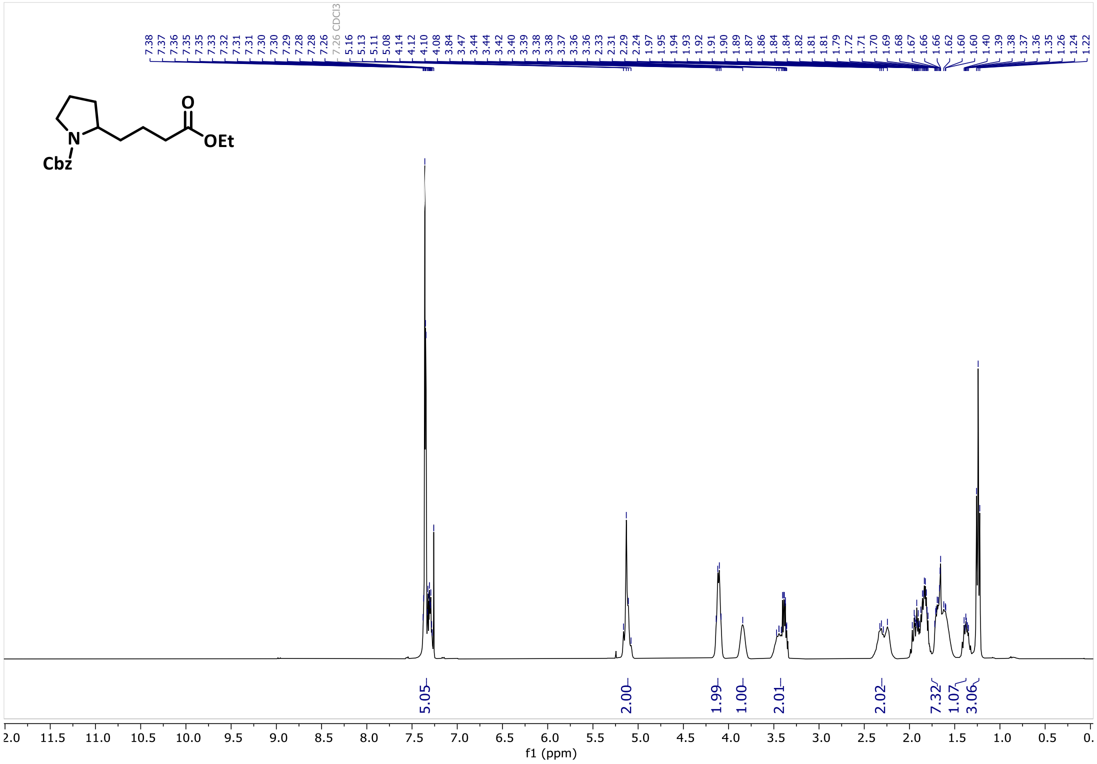

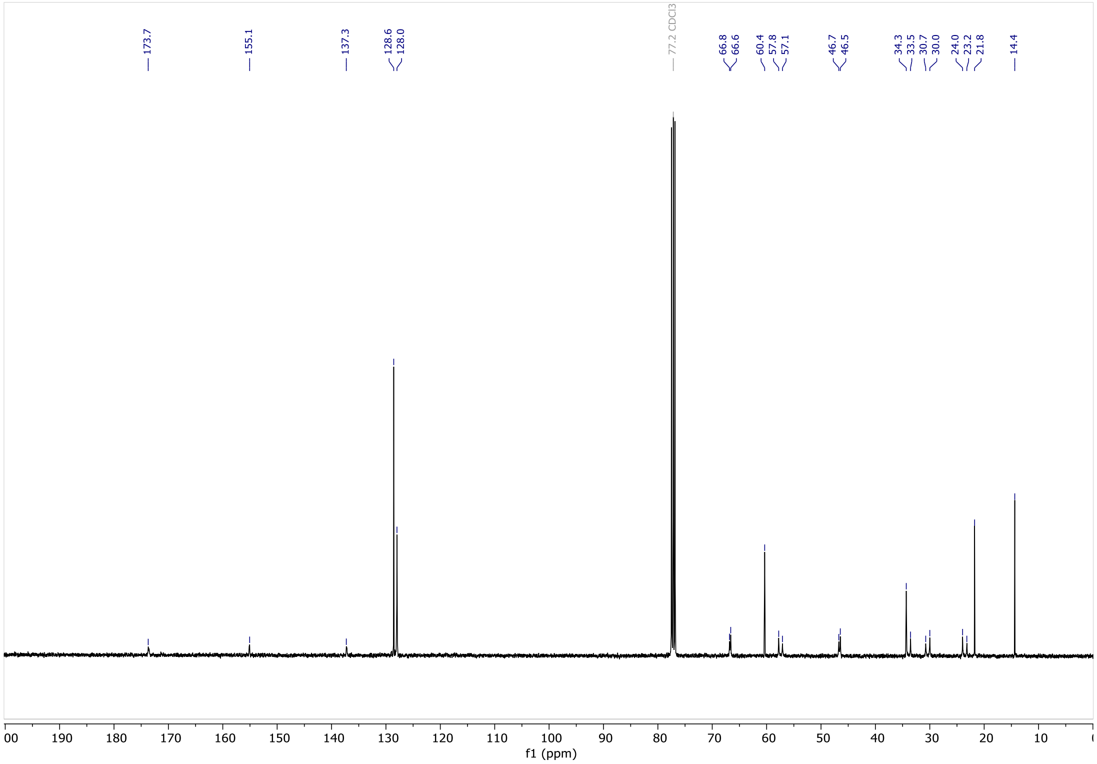

Benzyl 2-(4,4,4-trifluorobutyl)pyrrolidine-1-carboxylate (3n)

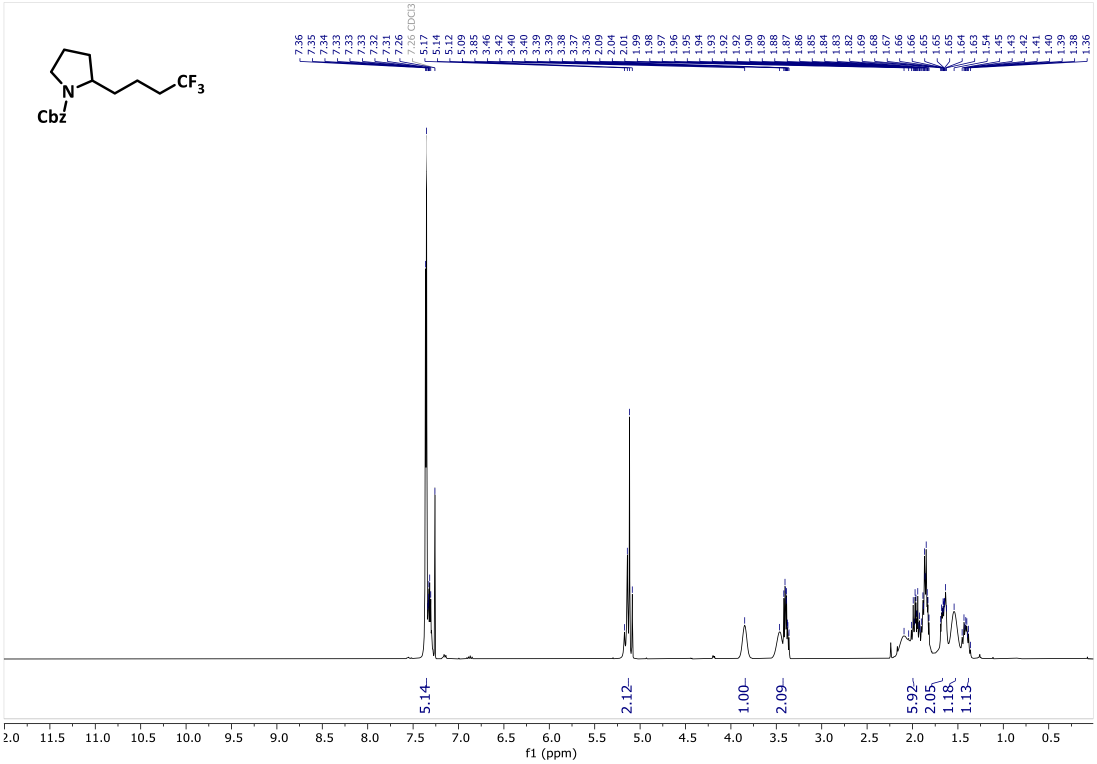

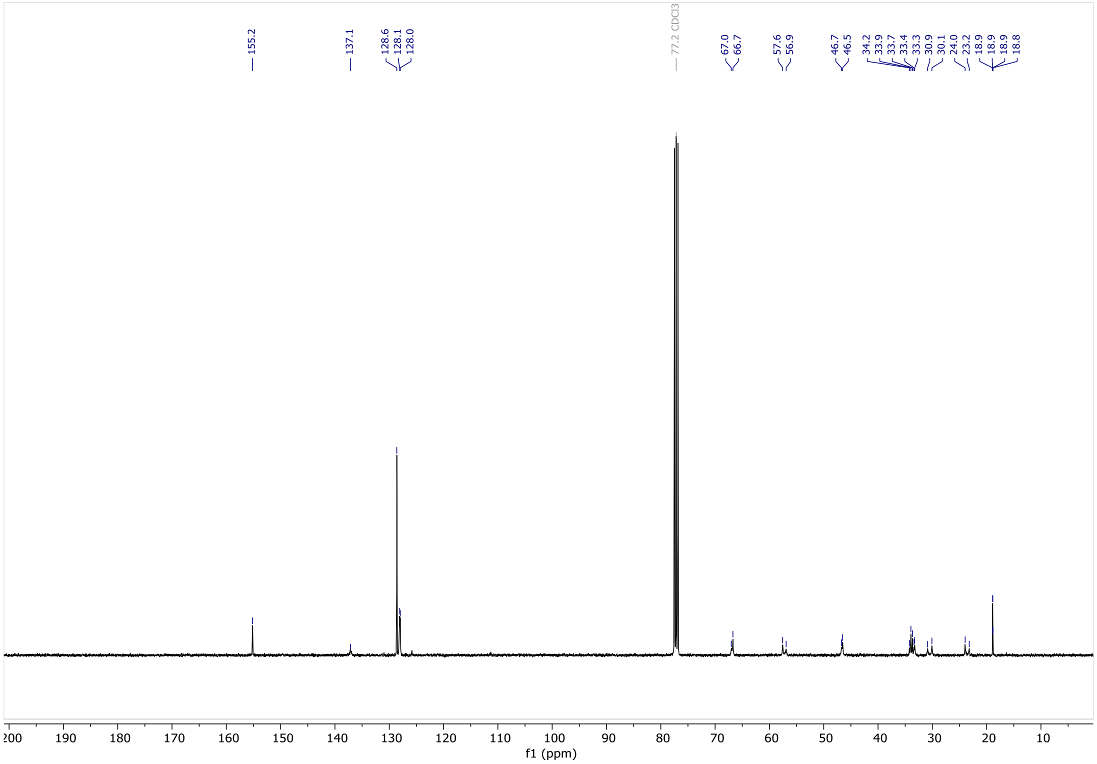

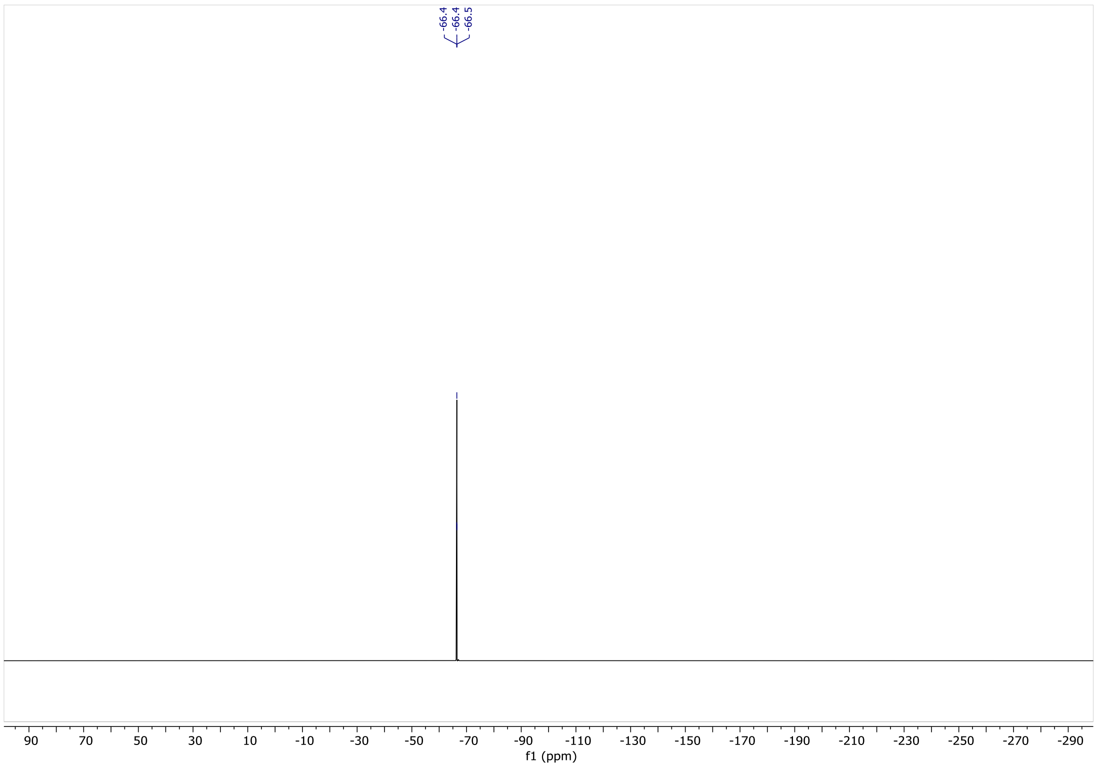
-66.4
-66.4
-66.5
90
70
50
30
10
-10
-30
-50
-70
-90
-110
-130
-150
-170
-190
-210
-230
-250
-270
-290
f1 (ppm)

***tert*-Butyl-2-(2-(benzyloxy)ethyl)pyrrolidine-1-carboxylate (3o)**

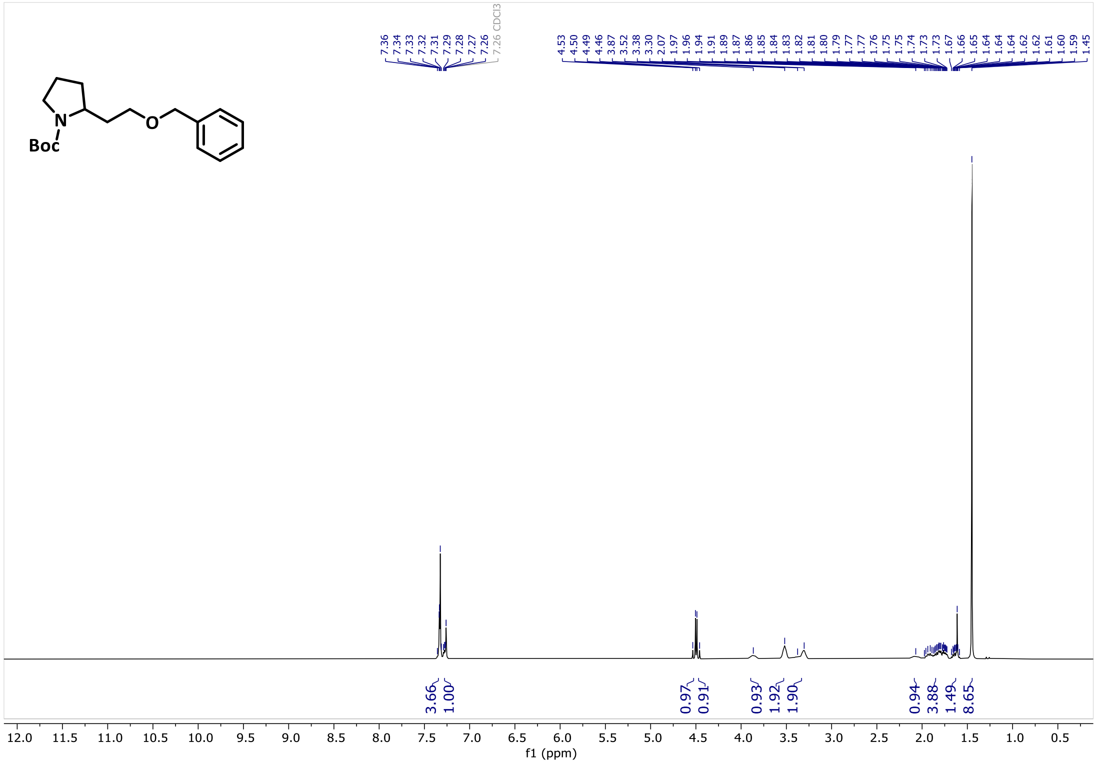

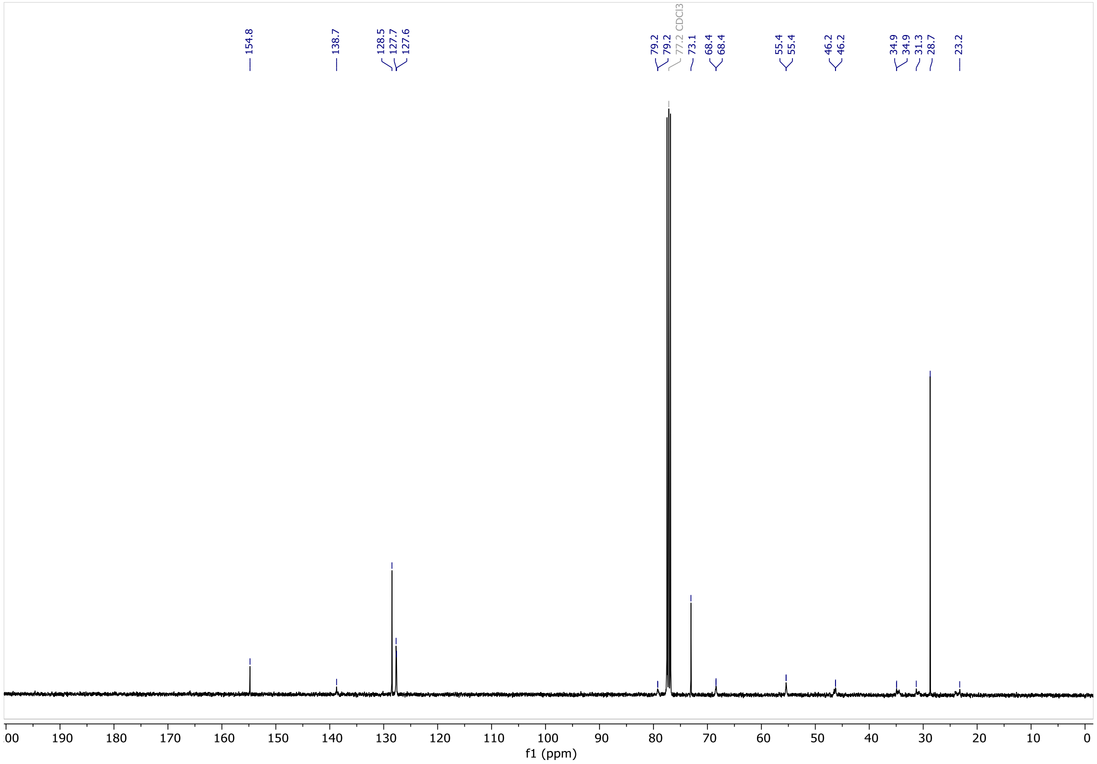

***tert*-Butyl 2-(pent-4-en-1-yl)pyrrolidine-1-carboxylate (3p)**

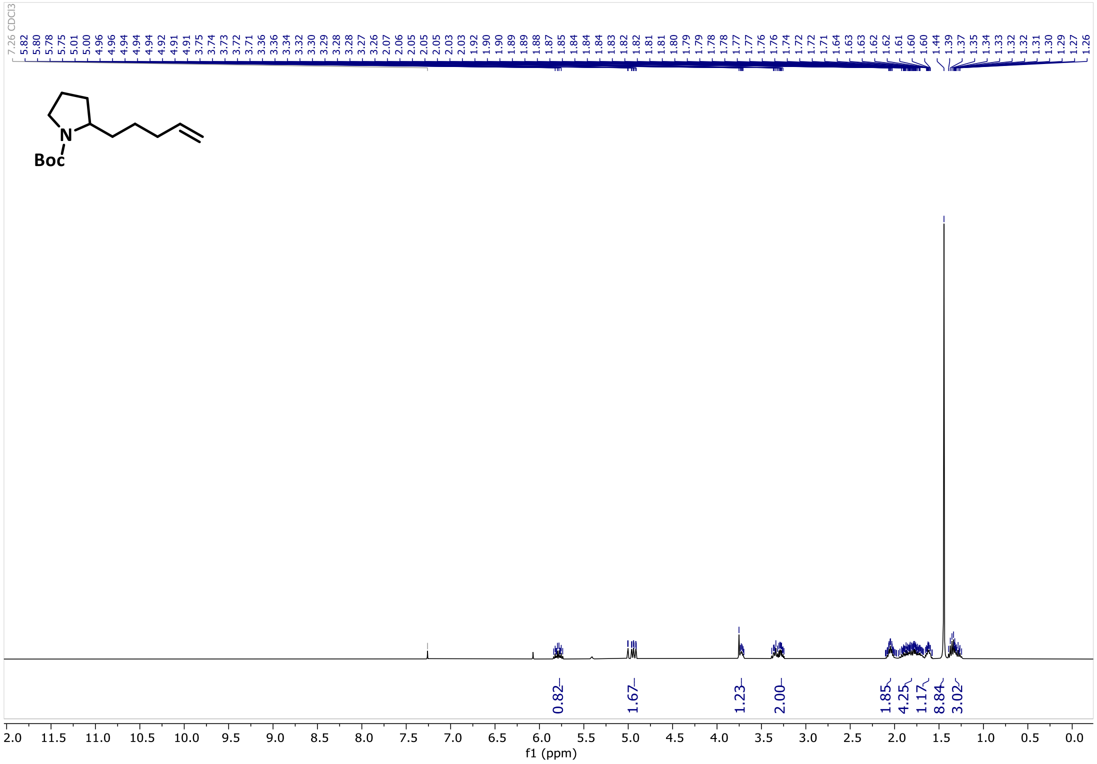

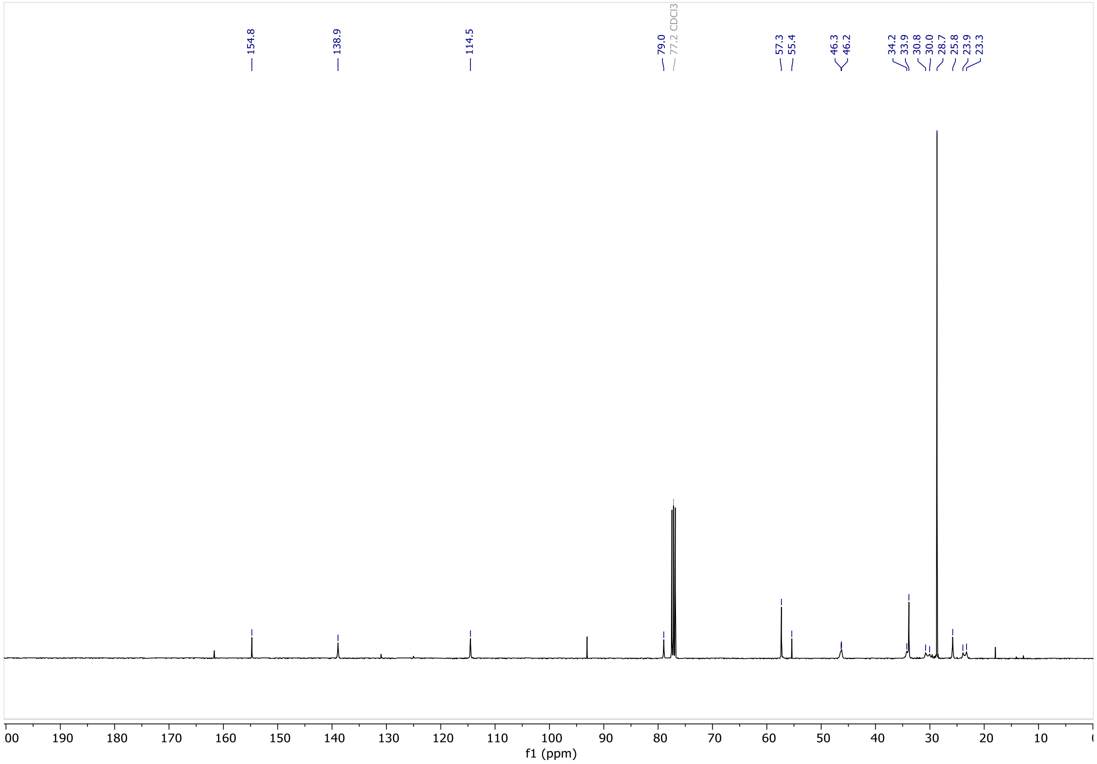

tert-Butyl 2-(3-hydroxypropyl)pyrrolidine-1-carboxylate (3q)

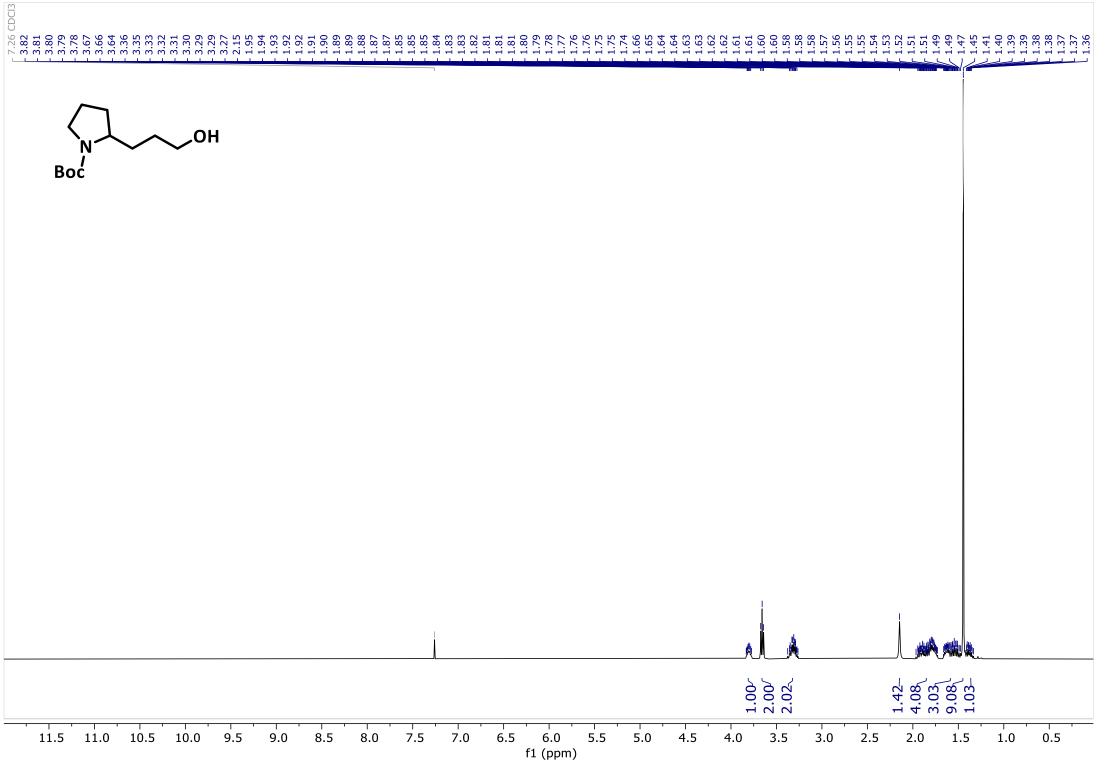

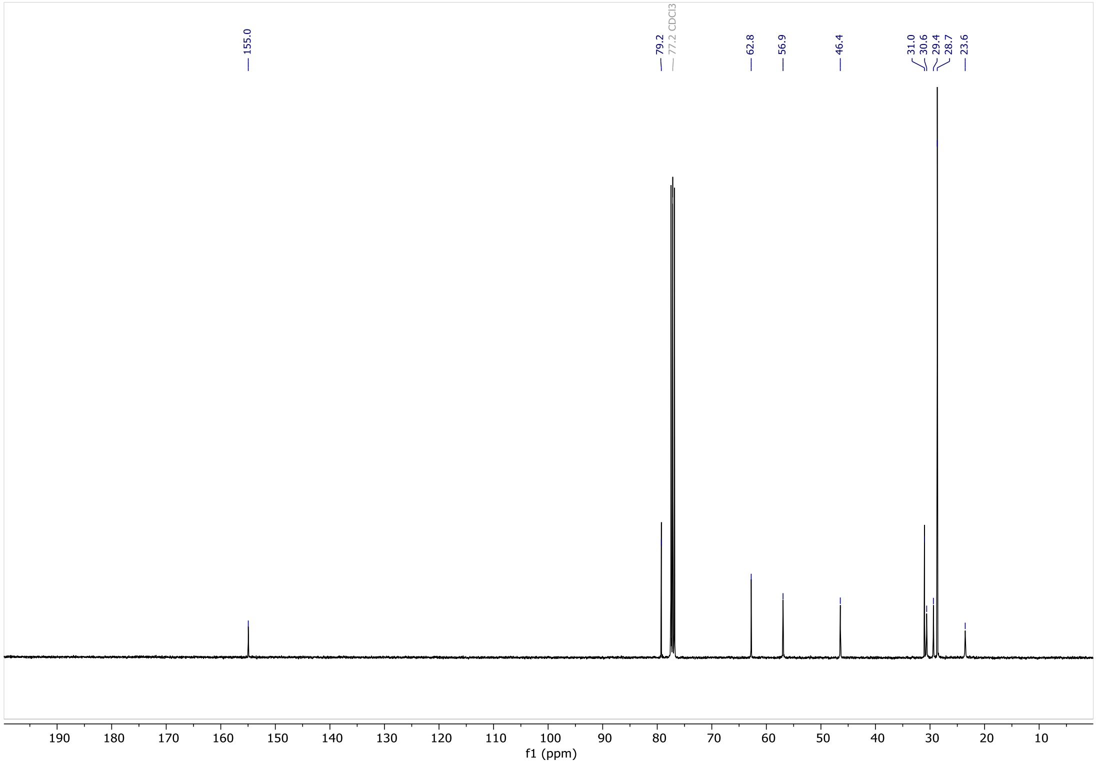

Benzyl 2-(3-chloropropyl)pyrrolidine-1-carboxylate (3r)

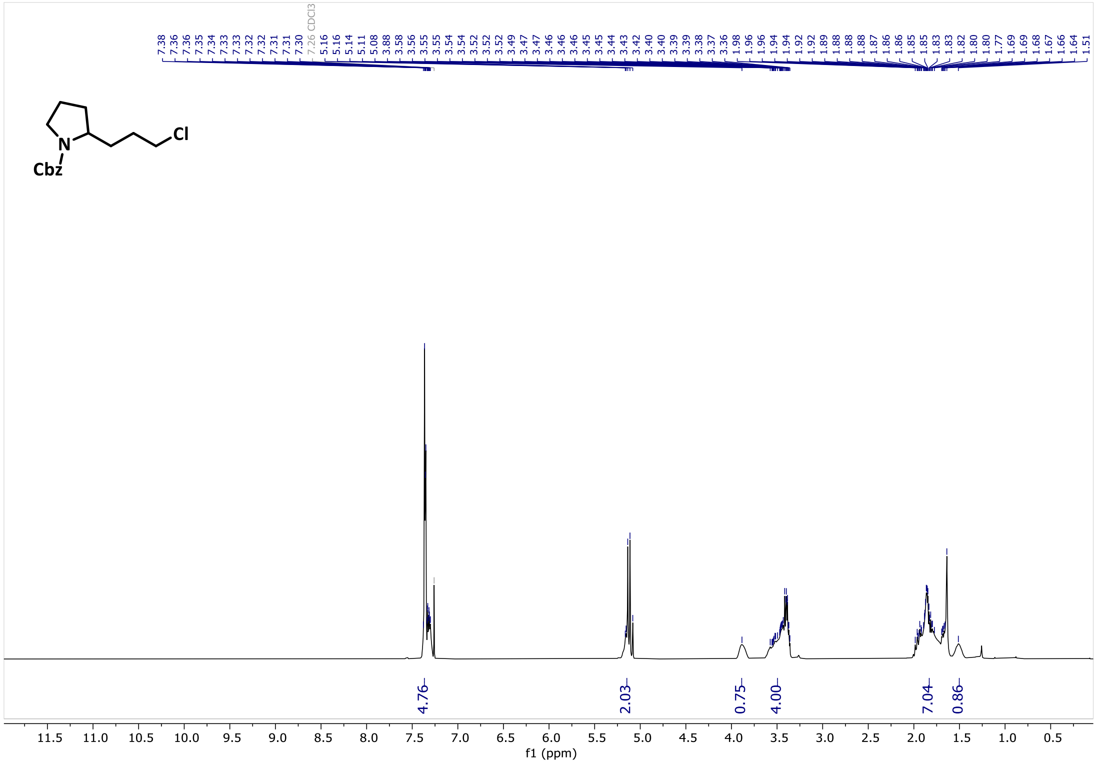

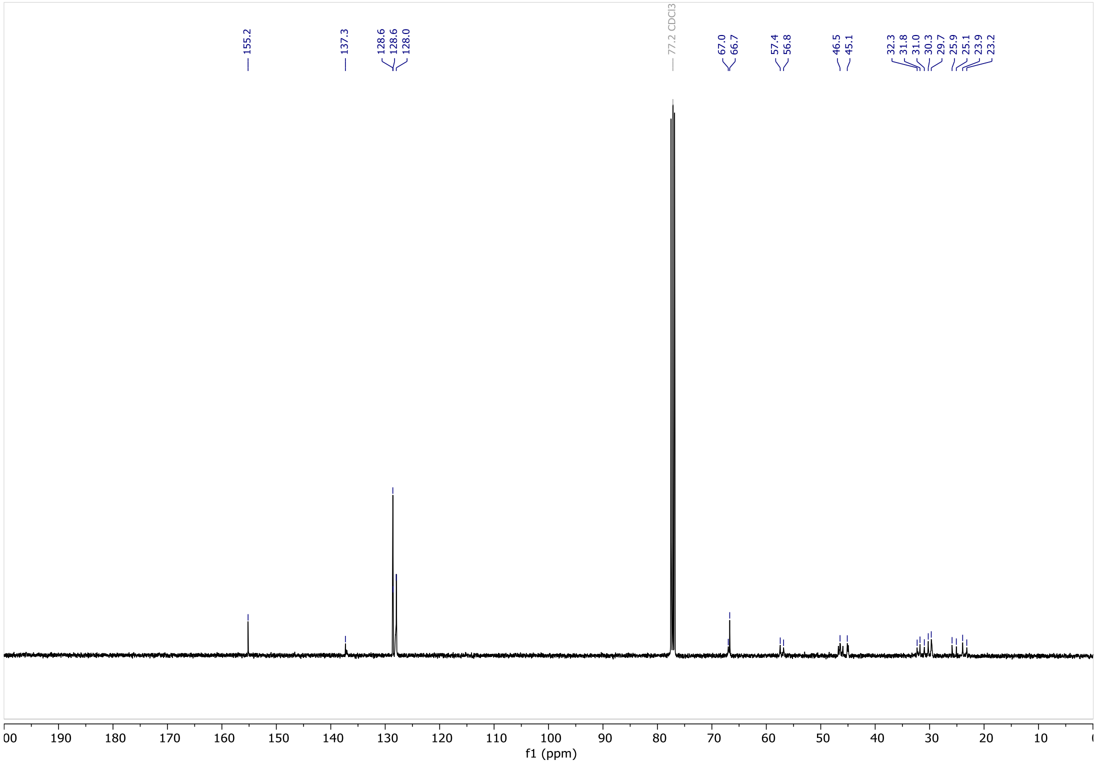

tert-butyl 2-neopentylpyrrolidine-1-carboxylate (3s)

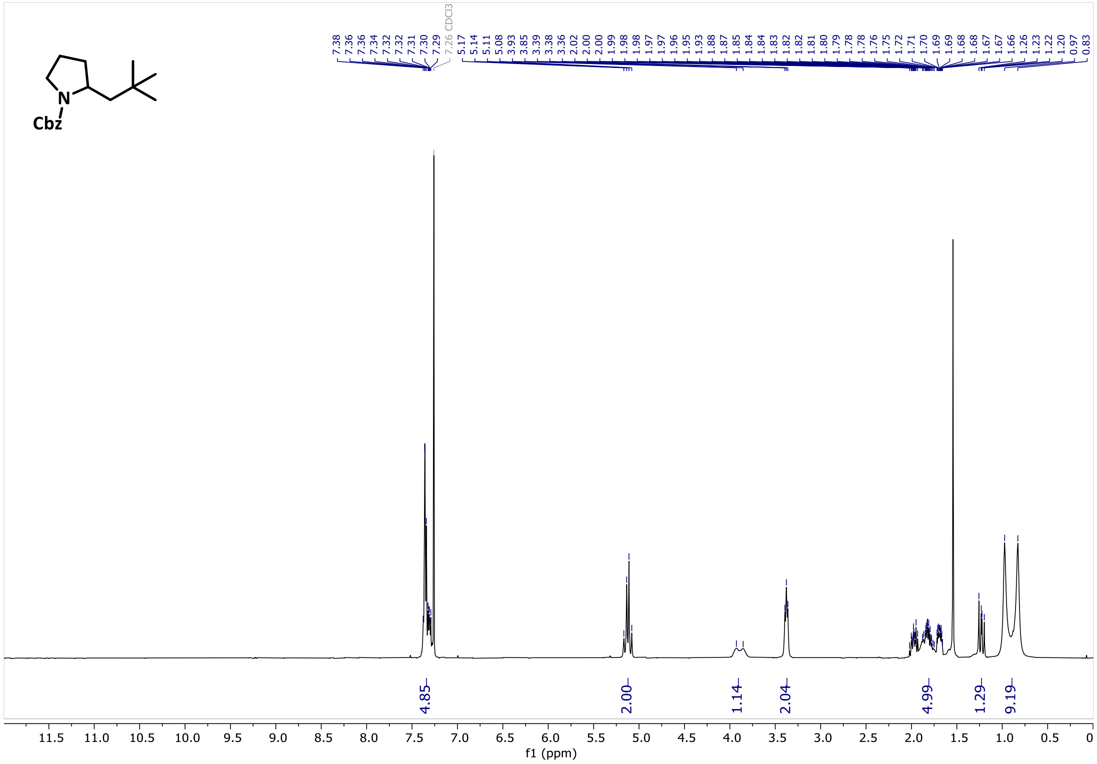

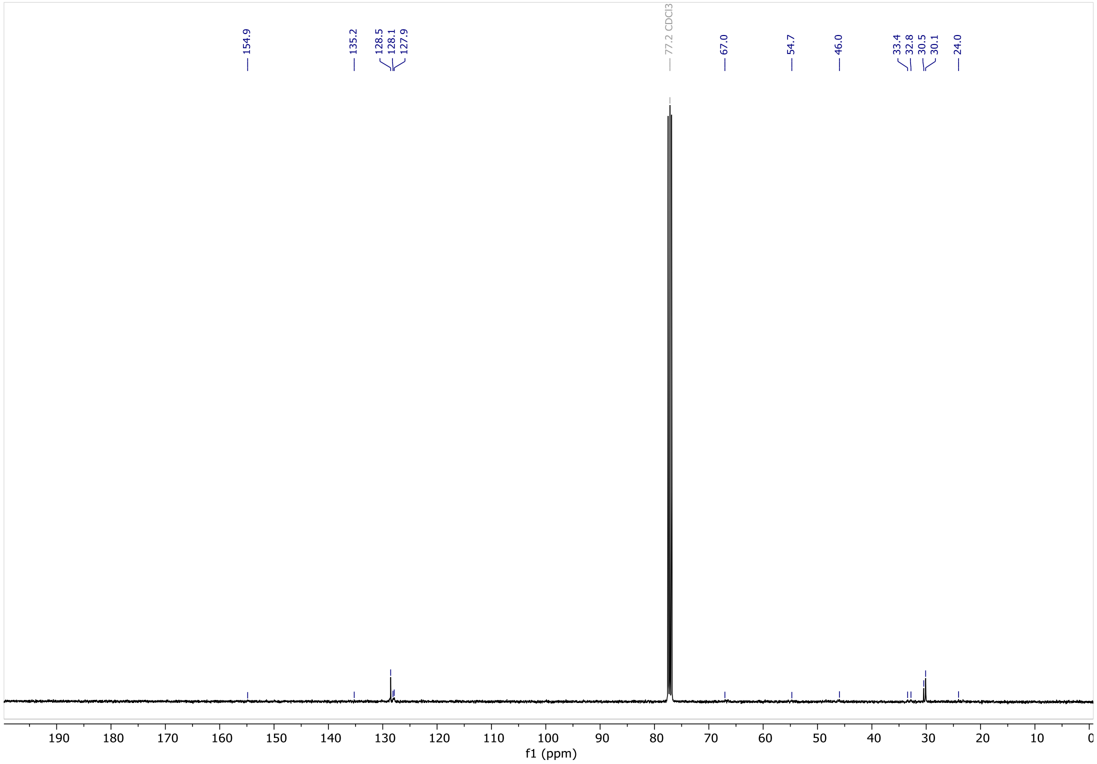

Benzyl 2-(oxetan-3-yl)pyrrolidine-1-carboxylate (3t)

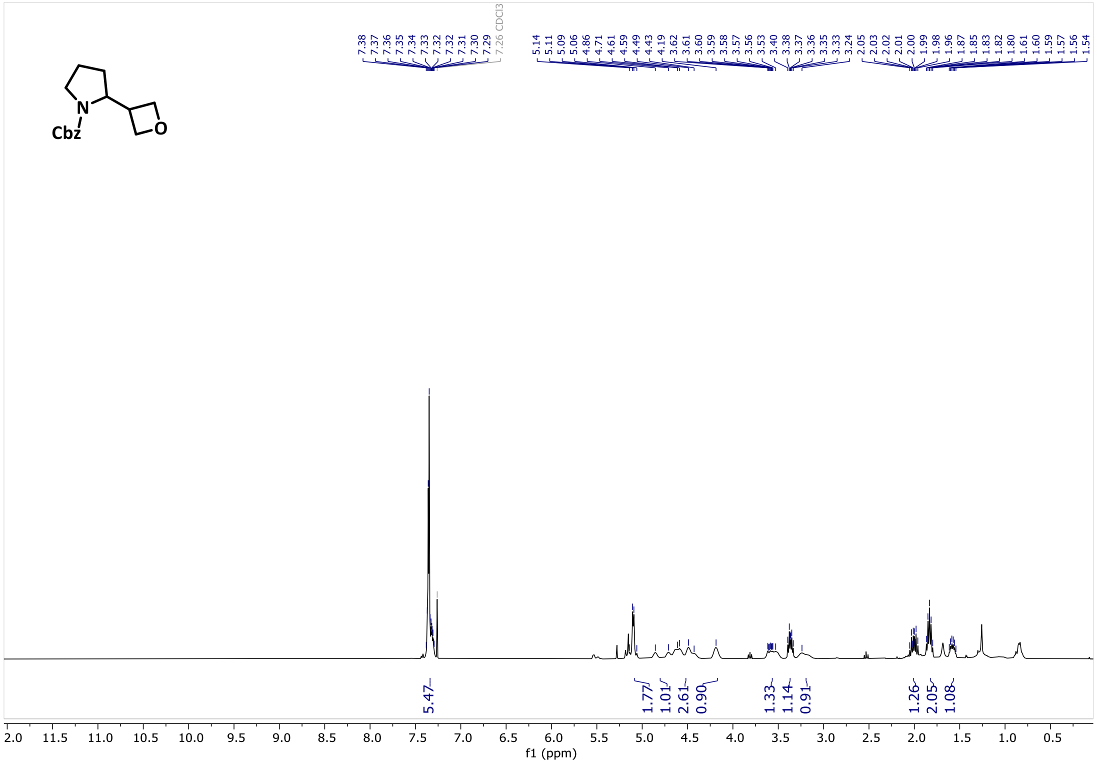

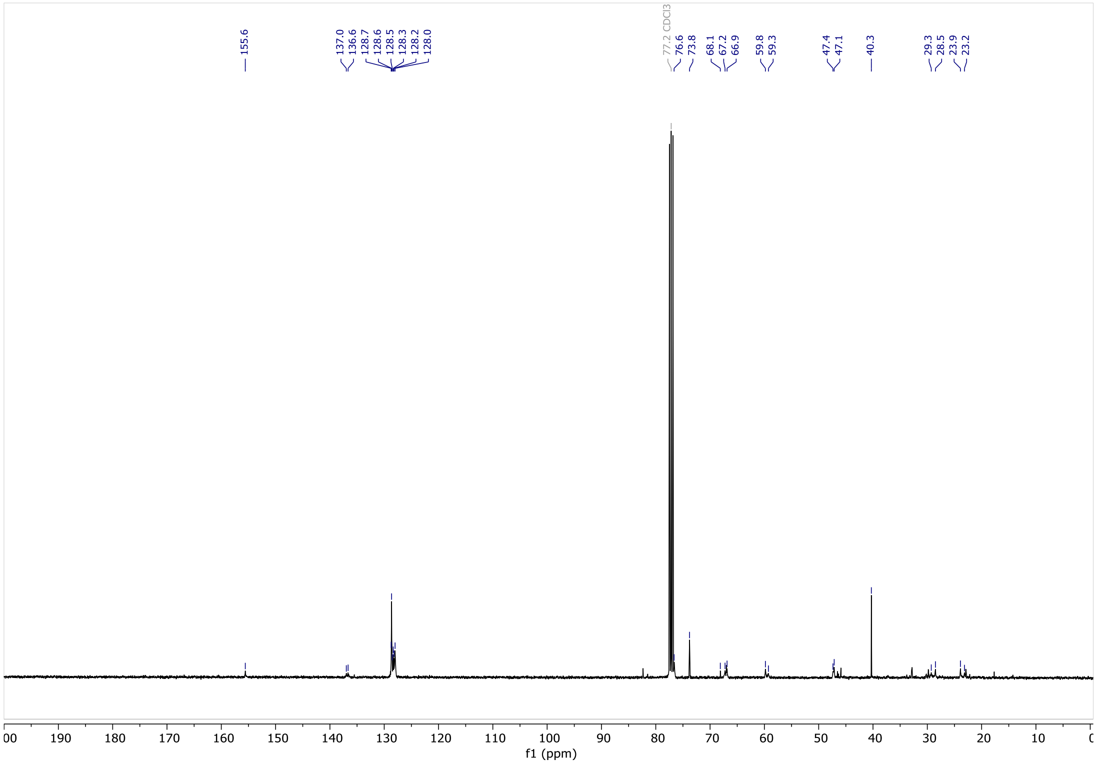

Benzyl 2-(tetrahydropyran-4-yl)pyrrolidine-1-carboxylate) (3u)

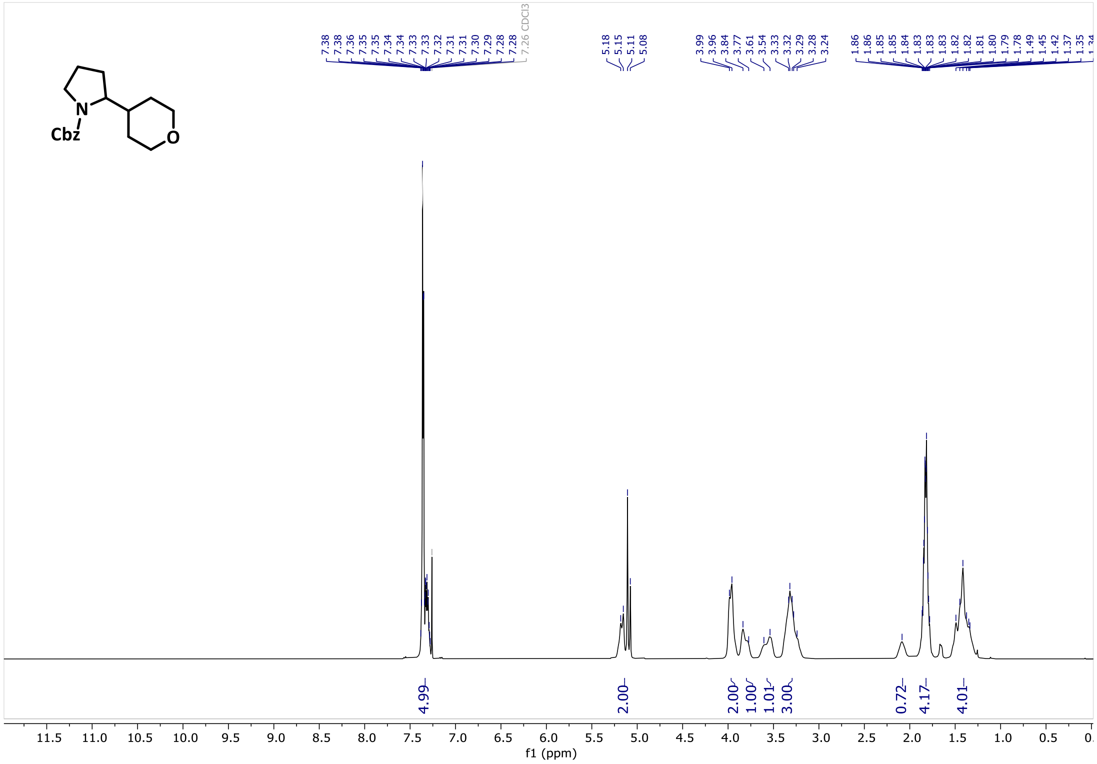

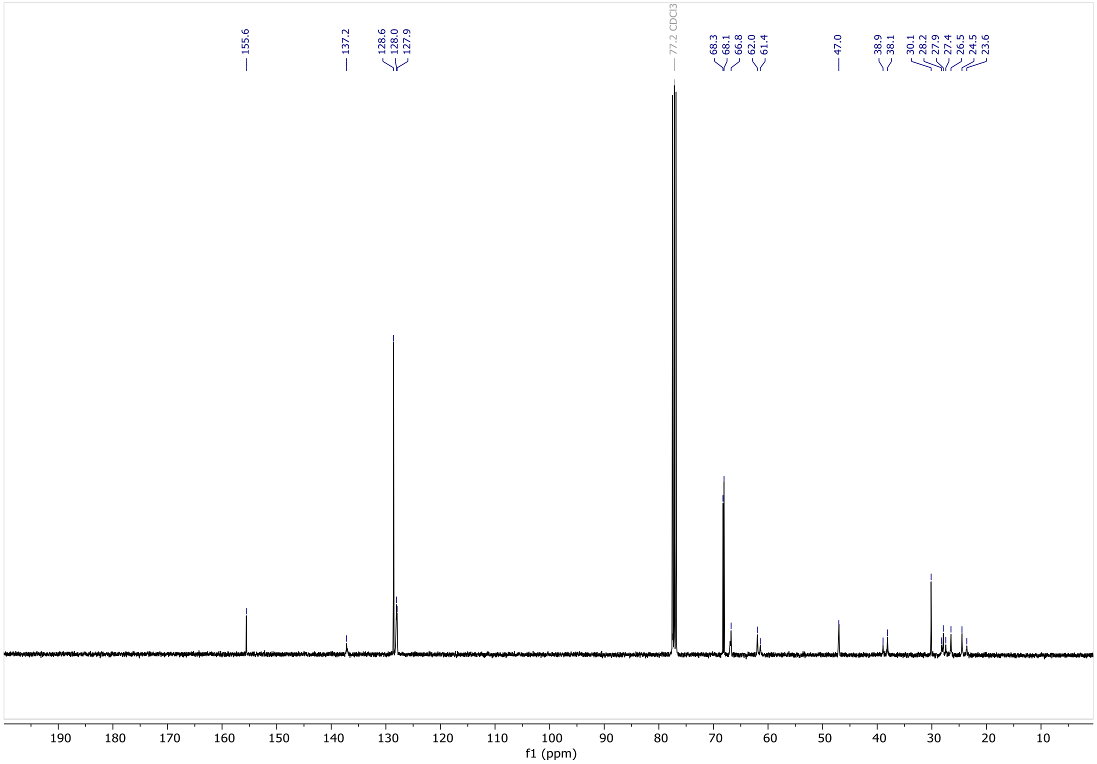

Benzyl 2-cyclopentylpyrrolidine-1-carboxylate (3v)

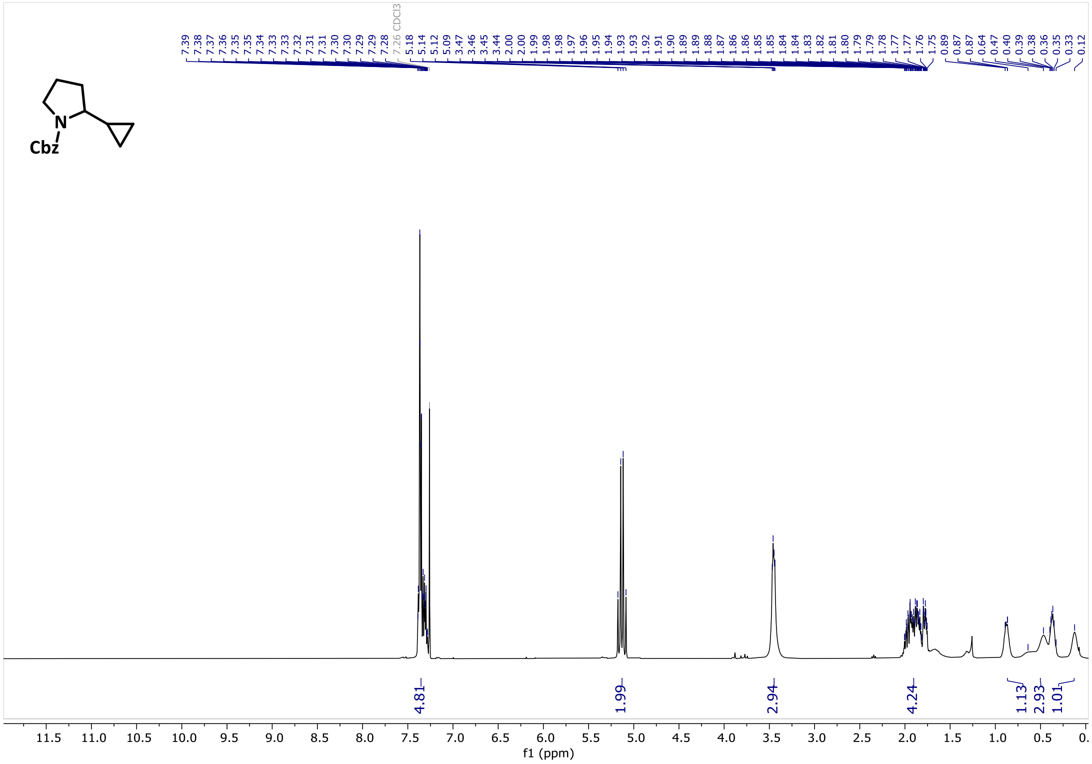

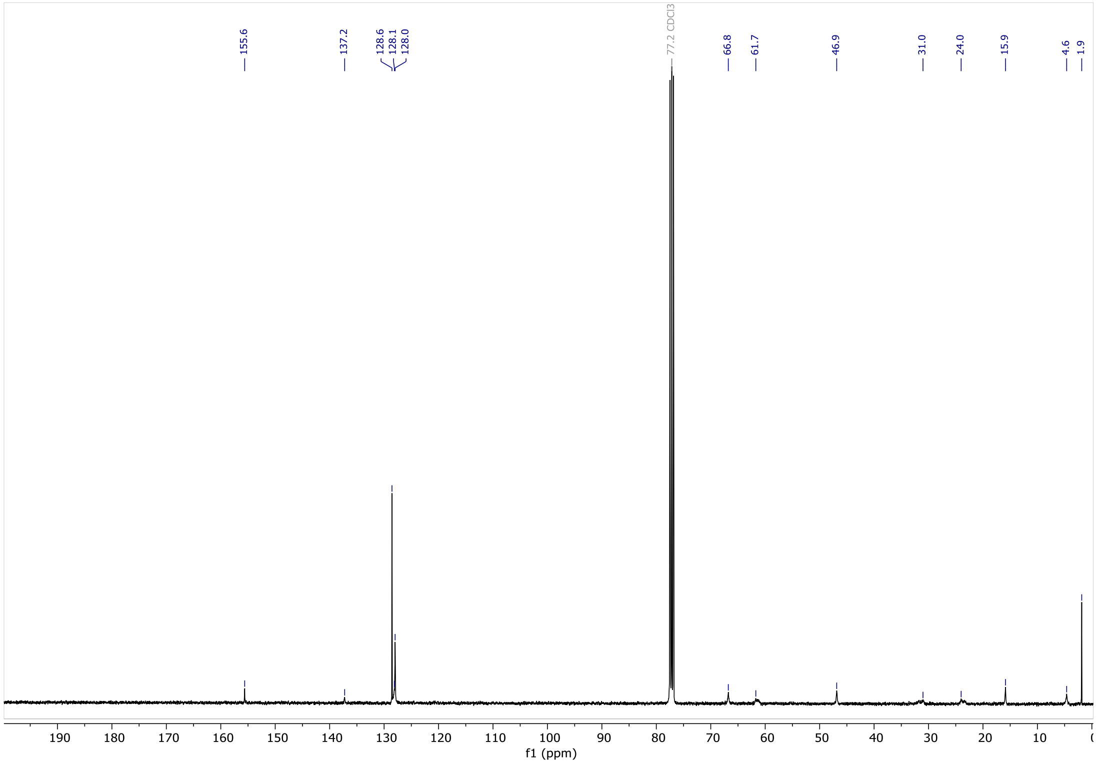

Benzyl 2-cyclopentylpyrrolidine-1-carboxylate (3w)

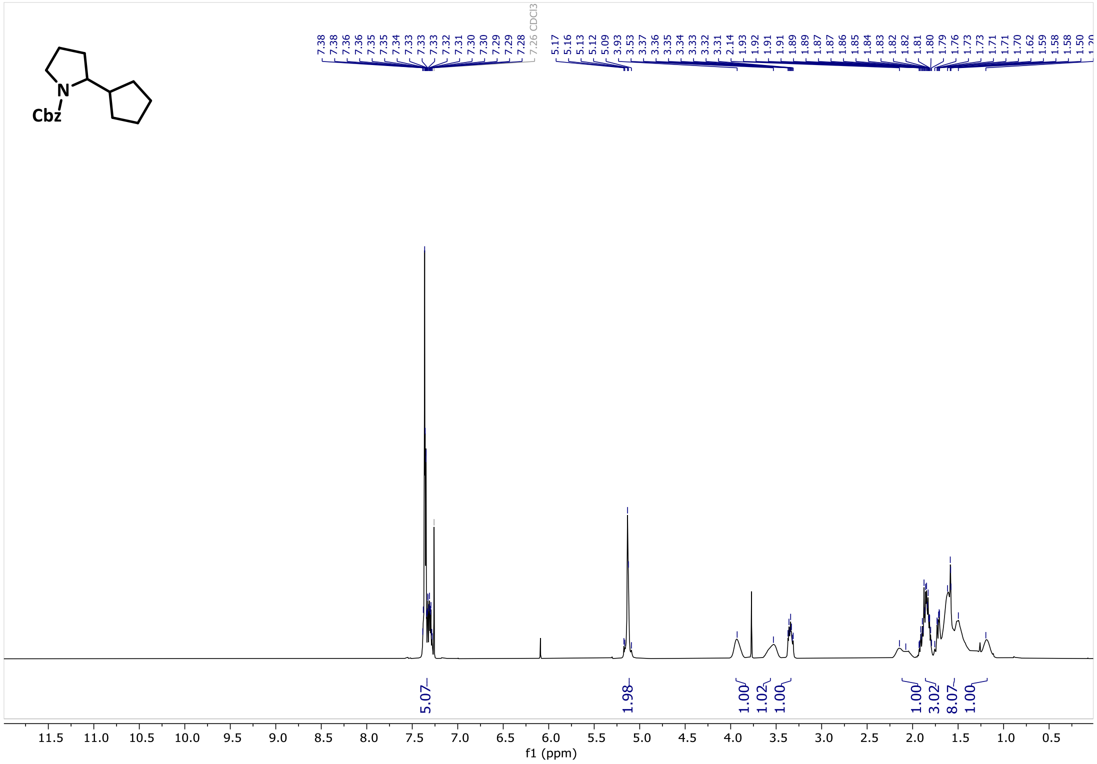

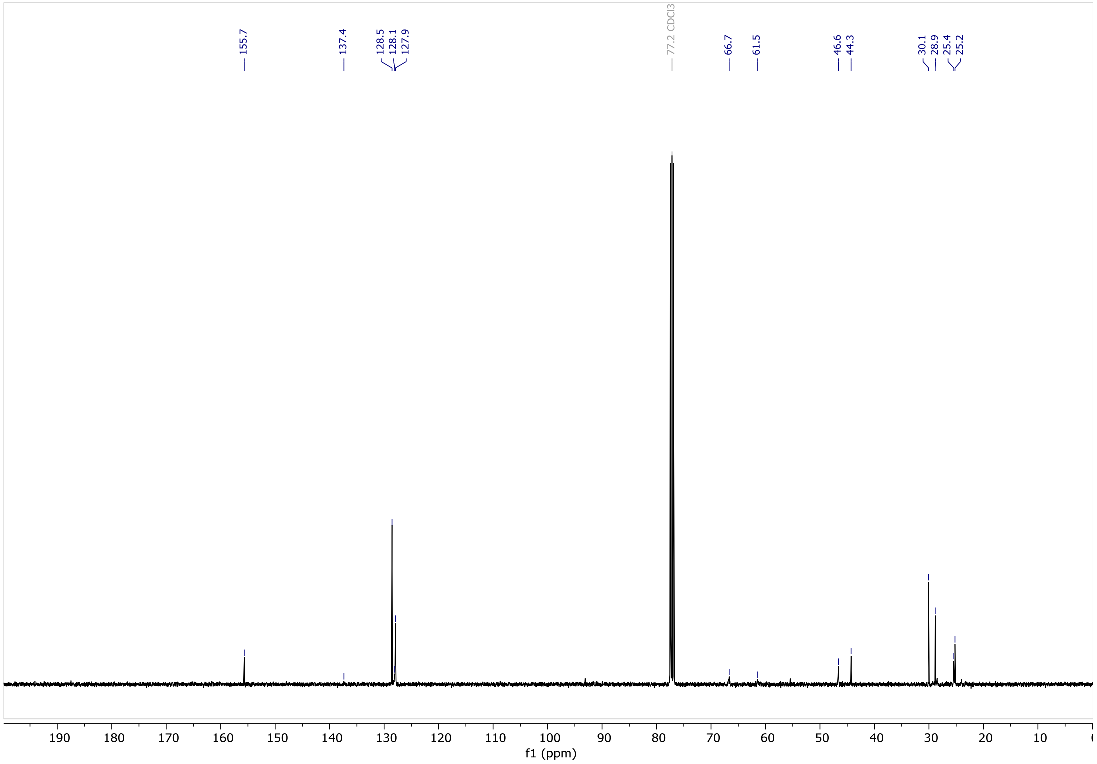

***tert*-Butyl 2-cyclohexylpyrrolidine-1-carboxylate (3x)**

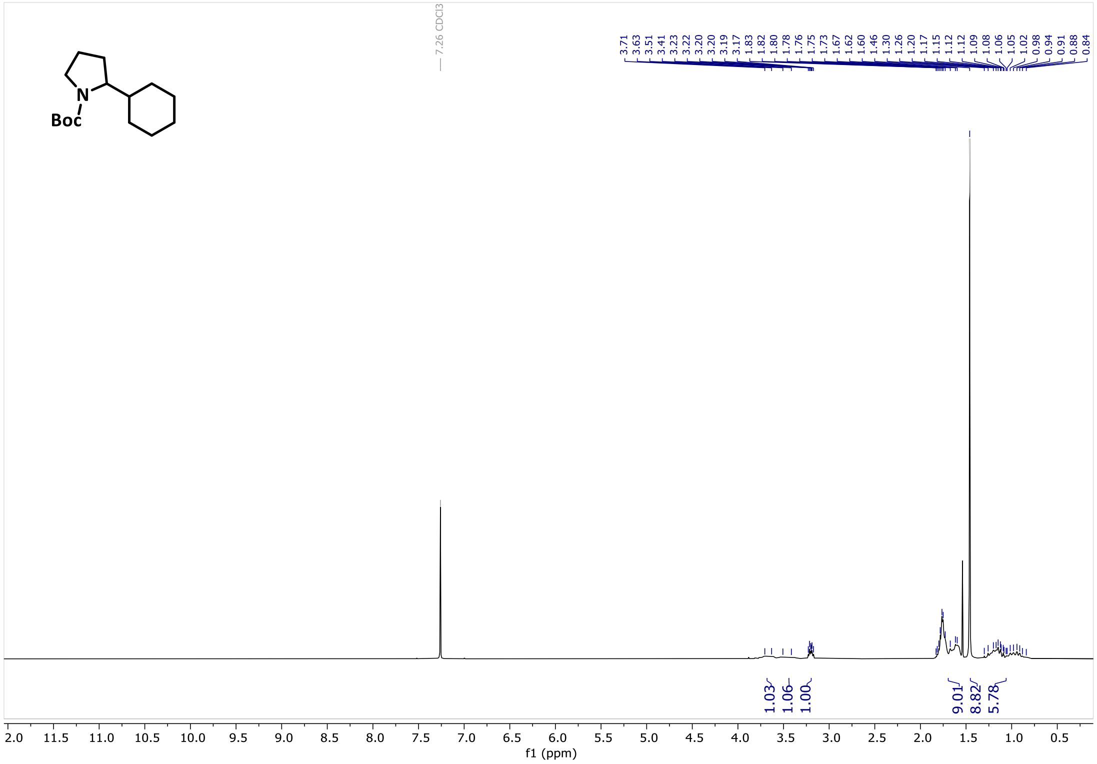

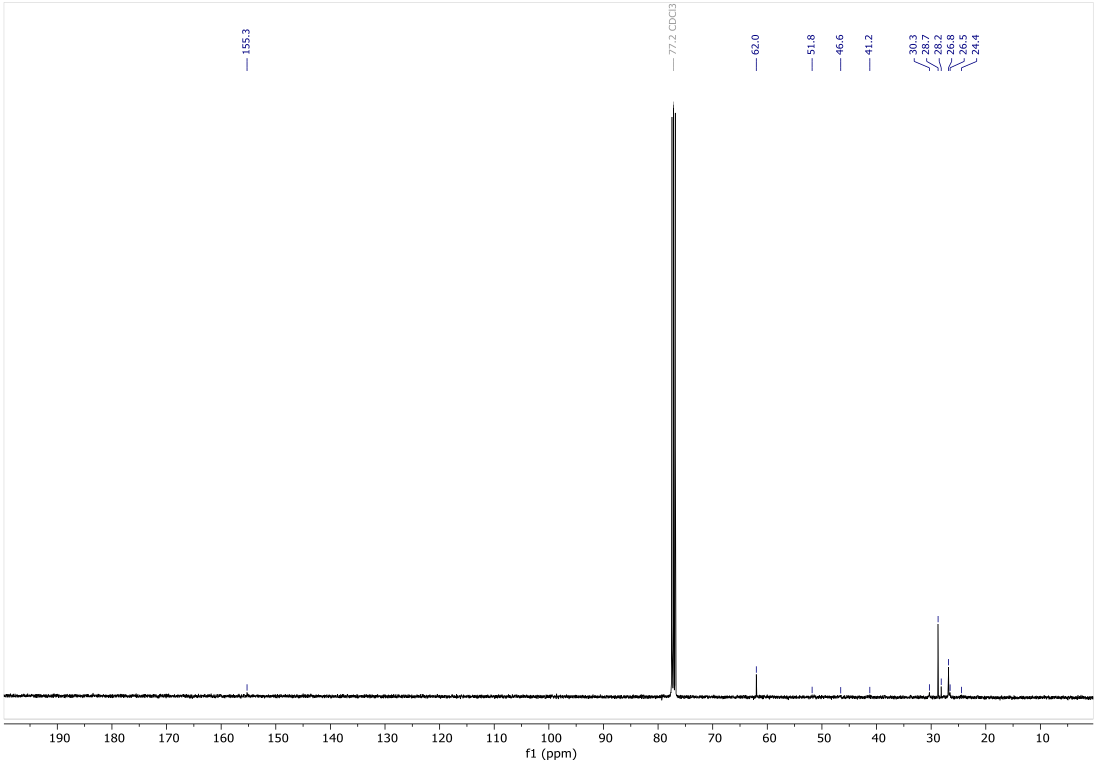

***tert*-butyl (1-(3,4-dimethoxyphenyl)-5-phenylpentan-2-yl)carbamate (3y)**

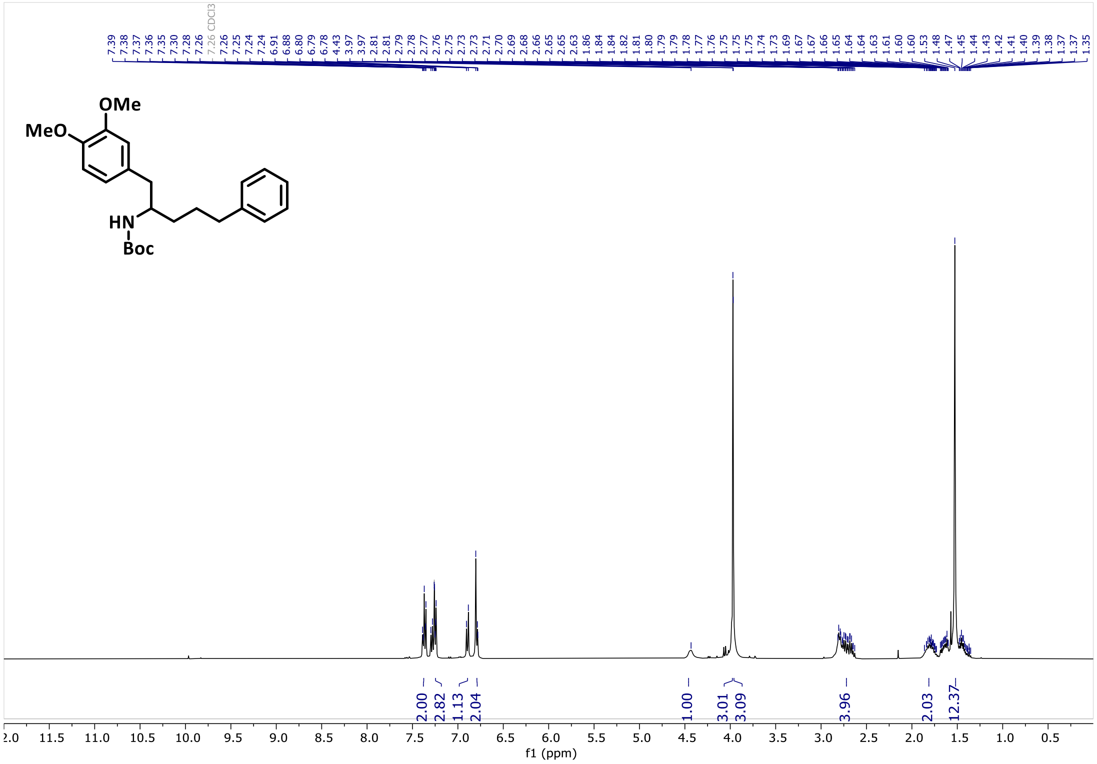

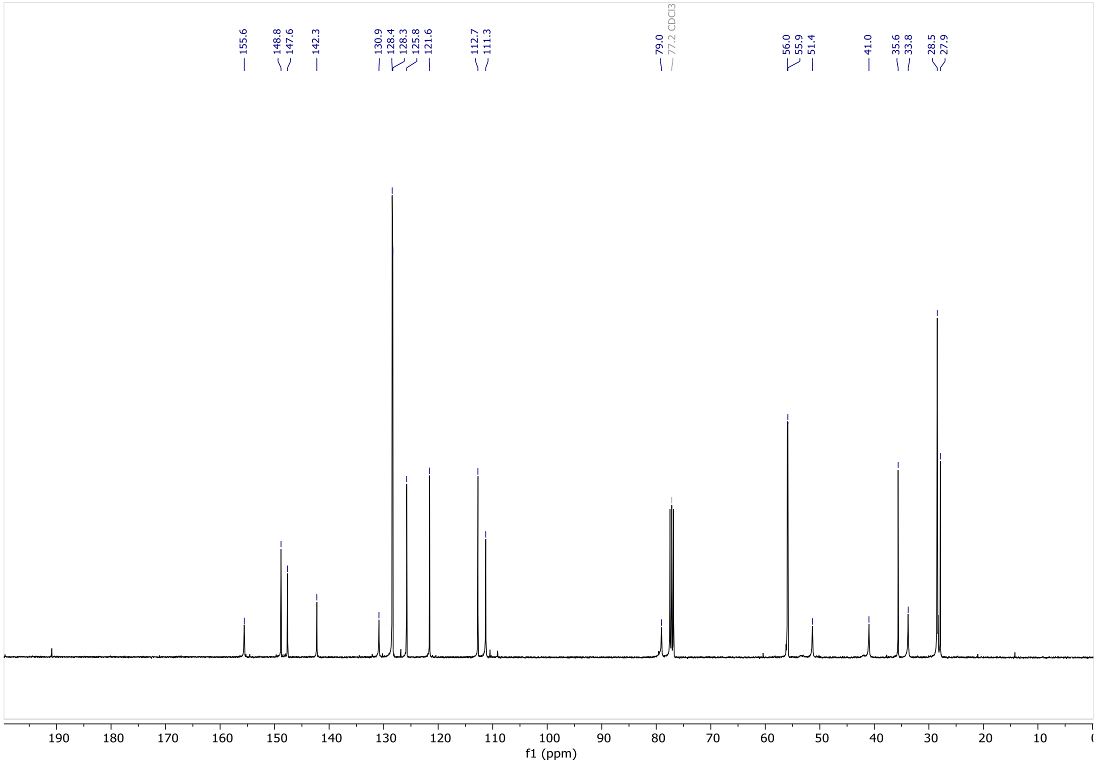

Supplementary references.

(1) Ruta, V.; Sivo, A.; Bonetti, L.; Bajada, M. A.; Vilé, G. Structural Effects of Metal Single-Atom Catalysts for Enhanced Photocatalytic Degradation of Gemfibrozil. *ACS Appl. Nano Mater.* **2022**, *5* (10), 14520–14528.

(2) Sivo, A.; Ruta, V.; Granata, V.; Savateev, O.; Bajada, M. A.; Vilé, G. Nanostructured Carbon Nitride for Continuous-Flow Trifluoromethylation of (Hetero)Arenes. *ACS Sustainable Chem. Eng.* **2023**, *11* (13), 5284–5292.

(3) Wang, W.; Shu, Z.; Liao, Z.; Zhou, J.; Meng, D.; Li, T.; Zhao, Z.; Xu, L. Sustainable One-Step Synthesis of Nanostructured Potassium Poly(Heptazine Imide) for Highly Boosted Photocatalytic Hydrogen Evolution. *J. Chem. Eng.* **2021**, *424* (130332).

(4) Savateev, A.; Tarakina, N. V.; Strauss, V.; Hussain, T.; ten Brummelhuis, K.; Sánchez Vadillo, J. M.; Markushyna, Y.; Mazzanti, S.; Tyutyunnik, A. P.; Walczak, R.; Oschatz, M.; Guldi, D. M.; Karton, A.; Antonietti, M. Potassium Poly(Heptazine Imide): Transition Metal-Free Solid-State Triplet Sensitizer in Cascade Energy Transfer and [3+2]-Cycloadditions. *Angew. Chem. Int. Ed.* **2020**, *59* (35), 15061–15068.

(5) Lee, W.; Kim, D.; Seo, S.; Chang, S. Photoinduced α-C−H Amination of Cyclic Amine Scaffolds Enabled by Polar-Radical Relay. *Angew. Chem. Int. Ed.* **2022**, *61* (25), e202202971.

(6) Smith't, S. R. P.; Dravnieks, F.; Wertz, J. E.; Orton, J.; Auzins, P.; Wertz, J. E. Electron-Paramagnetic-Resonance Line Shape of Ni'$^{+}$ in MgO*. *Phys. Rev.* **1969**, *178* (2), 471–480.

(7) Krzystek, J.; Park, J. H.; Meisel, M. W.; Hitchma, M. A.; Stratemeier, H.; Brunel, L. C.; Telser, J. EPR Spectra from "EPR-Silent" Species: High-Frequency and High-Field EPR Spectroscopy of Pseudotetrahedral Complexes of Nickel(II). *Inorg. Chem.* **2002**, *41* (17), 4478–4487.

(8) Yano, J.; Sauer, K.; Girerd, J. J.; Yachandra, V. K. Single Crystal X- and Q-Band EPR Spectroscopy of a Binuclear Mn_2(III,IV) Complex Relevant to the Oxygen-Evolving Complex of Photosystem II. *J. Am. Chem. Soc.* **2004**, *126* (24), 7486–7495.

(9) Marchi, M.; Raciti, E.; Gali, S. M.; Piccirilli, F.; Vondracek, H.; Actis, A.; Salvadori, E.; Rosso, C.; Criado, A.; D'Agostino, C.; Forster, L.; Lee, D.; Foucher, A. C.; Rai, R. K.; Beljonne, D.; Stach, E. A.; Chiesa, M.;

Lazzaroni, R.; Filippini, G.; Prato, M.; Melchionna, M.; Fornasiero, P. Carbon Vacancies Steer the Activity in Dual Ni Carbon Nitride Photocatalysis. *Adv. Sci.* **2023**, *10* (26).

(10) Jacobs, S. A.; Margerum, D. W. Solution Properties of Bis(Dipeptide)Nickelate(III) Complexes and Kinetics of Their Decomposition in Acid. *Inorg. Chem* **1984**, *23* (9), 1195–1201.

(11) Henry, R. J. W.; Division, S.; Peak [1]tional Observatory, E.; Tucson, A.; Burke, P. G.; Sinfailam, A.-L. Scattering of Electrons by C, N, O, N^+, O^+, and O^{++}. *Phisc. Rev.* **1964**, *178* (1), 218–225.

(12) Actis, A.; Melchionna, M.; Filippini, G.; Fornasiero, P.; Prato, M.; Salvadori, E.; Chiesa, M. Morphology and Light-Dependent Spatial Distribution of Spin Defects in Carbon Nitride. *Angew. Chem. Int. Ed.* **2022**, *61* (43), e20221064.

(13) Kresse, G.; Hafner, J. Ab. Initio Molecular Dynamics for Liquid Metals. *Phys. Rev. B* **1993**, *47* (1), 558–561.

(14) Kresse, G.; Furthmü, J. Efficient Iterative Schemes for Ab Initio Total-Energy Calculations Using a Plane-Wave Basis Set. *Phys. Rev. B* **1996**, *54* (16), 11169–11186.

(15) Kresse, G.; Furthmiiller B, J. Efficiency of Ab-Initio Total Energy Calculations for Metals and Semiconductors Using a Plane-Wave Basis Set. *Comput. Mater. Sci.* **1996**, *6* (1), 15–50.

(16) Kresse, G.; Joubert, D. From Ultrasoft Pseudopotentials to the Projector Augmented-Wave Method. *Phys. Rev. B* **1999**, *59* (3), 1758–1775.

(17) Blochl, P. E. Projector Augmented-Wave Method. *Phys. Rev. B* **1994**, *50* (24), 17953–17979.

(18) Perdew, J. P.; Burke, K.; Ernzerhof, M. Generalized Gradient Approximation Made Simple. *Phys. Rev. Lett* **1996**, *77* (18), 3865–3868.

(19) Grimme, S.; Antony, J.; Ehrlich, S.; Krieg, H. A Consistent and Accurate Ab Initio Parametrization of Density Functional Dispersion Correction (DFT-D) for the 94 Elements H-Pu. *J. Chem. Phys.* **2010**, *132* (15), 154104.

(20) Grimme, S.; Ehrlich, S.; Goerigk, L. Effect of the Damping Function in Dispersion Corrected Density Functional Theory. *J. Comput. Chem.* **2011**, *32* (7), 1456–1465.

(21) Perdew, J. P.; Ernzerhof, M.; Burke, K. Rationale for Mixing Exact Exchange with Density Functional Approximations. *J. Chem. Phys.* **1996**, *105* (22), 9982–9985.

(22) Adamo, C.; Barone, V. Toward Reliable Density Functional Methods without Adjustable Parameters: The PBE0 Model. *J. Chem. Phys.* **1999**, *110* (13), 6158–6170.

(23) Meroni, D.; Lo Presti, L.; Di Liberto, G.; Ceotto, M.; Acres, R. G.; Prince, K. C.; Bellani, R.; Soliveri, G.; Ardizzone, S. A Close Look at the Structure of the TiO_2-APTES Interface in Hybrid Nanomaterials and Its Degradation Pathway: An Experimental and Theoretical Study. *J. Phys. Chem. C* **2017**, *121* (1), 430–440.

(24) Nørskov, J. K.; Bligaard, T.; Logadottir, A.; Kitchin, J. R.; Chen, J. G.; Pandelov, S.; Stimming, U. Trends in the Exchange Current for Hydrogen Evolution. *J. Electrochem. Soc.* **2005**, *152* (3), 23–26.

(25) Nørskov, J. K.; Rossmeisl, J.; Logadottir, A.; Lindqvist, L.; Kitchin, J. R.; Bligaard, T.; Jónsson, H. Origin of the Overpotential for Oxygen Reduction at a Fuel-Cell Cathode. *J. Phys. Chem. B* **2004**, *108* (46), 17886–17892.

(26) Johnston, C. P.; Smith, R. T.; Allmendinger, S.; MacMillan, D. W. C. Metallaphotoredox-Catalysed sp^3–sp^3 Cross-Coupling of Carboxylic Acids with Alkyl Halides. *Nature* **2016**, *536* (7616), 322–325.
