# Supplementary material for: Photocatalytic C(sp3)–C(sp3) cross-coupling of carboxylic acids and alkyl halides using a nickel complex and carbon nitride
Source: Nat Commun. 2025 Jul 31;16:7016. doi: 10.1038/s41467-025-61639-8 (PMC12313896; doi:10.1038/s41467-025-61639-8)
Supplement: Supplementary file 3 — Source Data [file 41467_2025_61639_MOESM3_ESM.zip › NCOMMS-24-46423_R4_Source_Data_DFT.pdf]

DFT source data file

Below, we report the XYZ coordinates for the DFT-optimized entries, the catalyst, and each intermediate described in the manuscript. These structures were optimized using the VASP code with the recommended pseudopotentials, as specified in the manual.

Entry 1

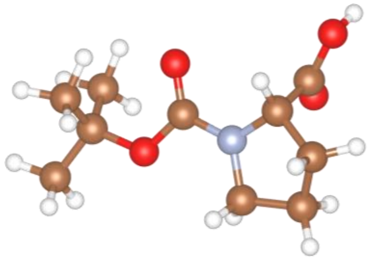

32
Lattice="30.0 0.0 0.0 0.0 30.0 0.0 0.0 0.0 30.0" Properties=species:S:1:pos:R:3 pbc="T T T"

C	17.07367432	14.93689947	16.70247252
C	18.00735664	13.70758675	16.74144144
C	17.44117808	12.80476950	15.63751574
C	15.92526865	13.00507062	15.74684347
C	14.67135739	15.11352830	16.35602946
C	12.24795294	14.97696989	15.94027634
C	17.57573557	15.97064249	15.69551225
C	12.19108117	16.18511729	15.00763829
C	11.38554853	13.83801811	15.40351625
C	11.84937131	15.32698028	17.37285490
H	16.98592675	15.43100529	17.68018599
H	17.91066480	13.22752051	17.72692080
H	19.06220686	13.97439503	16.59687753
H	17.79112053	13.15060370	14.65407307
H	17.73432743	11.75303512	15.75580026
H	15.41370165	12.93225490	14.77748955
H	15.45980167	12.27448337	16.43098290
H	12.52032054	15.90320103	13.99701054
H	11.15320307	16.54423468	14.94253303
H	12.82603246	16.99889414	15.37626500
H	11.46756691	12.95226775	16.04936714
H	11.69795257	13.56077873	14.38678290
H	10.33238989	14.15112935	15.37211831
H	12.47533005	16.13430731	17.77037079
H	10.79820287	15.65047615	17.38966520
H	11.94534880	14.44379501	18.02151318
H	18.72539294	17.46668391	15.58576788
O	13.60329193	14.38439660	15.92517461
O	18.41932249	16.85955338	16.29296179
O	14.65446842	16.27585821	16.76405246
O	17.31740844	15.99161916	14.50580950
N	15.80819201	14.36072164	16.29247720

Entry 2

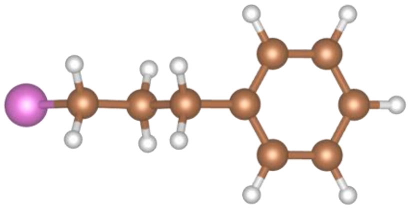

21
Lattice="30.0 0.0 0.0 0.0 30.0 0.0 0.0 0.0 30.0" Properties=species:S:1:pos:R:3 pbc="T T T"

C	15.30356192	15.18406759	16.40883651
C	16.34467922	15.21463582	15.27257319
C	13.89041551	15.19194166	15.88342350
C	17.75395655	15.16740749	15.82541135
C	13.22483427	16.39939080	15.63045408
C	13.22647847	13.99125749	15.59663769
C	11.93143733	16.40830467	15.10521676
C	11.93303414	13.99479667	15.07162209
C	11.28133155	15.20472775	14.82313152
H	15.46426110	14.28318421	17.02439633
H	15.46030127	16.05372736	17.06837560
H	16.20693348	16.12637410	14.67050935
H	16.17782883	14.36101922	14.59710445
H	17.98278026	16.02869163	16.46524873
H	17.95463406	14.24133950	16.37820747
H	13.72668944	17.34483907	15.85401531
H	13.73001762	13.04077649	15.79365489
H	11.42723926	17.35870252	14.91908577
H	11.43035152	13.04902831	14.85951480
H	10.26880302	15.20967375	14.41544916
Br	19.10442911	15.21541366	14.39133253

Entry IV

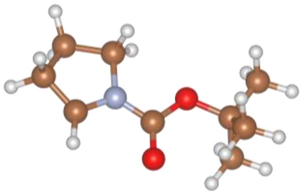

28
Lattice="30.0 0.0 0.0 0.0 30.0 0.0 0.0 0.0 30.0" Properties=species:S:1:pos:R:3 pbc="T T T"

C	17.44645705	15.19266636	16.00150325
C	18.43579342	14.09000398	15.91762164
C	17.80046573	13.15394522	14.86744740
C	16.29255643	13.38388116	15.03310868
C	15.06578913	15.51923962	15.62429347
C	12.65809795	15.38105221	15.12981662
C	12.61650493	16.61776335	14.23377463
C	11.81311717	14.25548286	14.53914144
C	12.22340217	15.68763764	16.56246760
H	17.52349367	16.16063805	16.48340323
H	18.55379453	13.55392159	16.88669839
H	19.44451323	14.42907811	15.63398876
H	18.11407318	13.46416862	13.85977902
H	18.07366123	12.09878833	15.00111976
H	15.72242966	13.29469324	14.10082557
H	15.84410360	12.69994022	15.77258208
H	12.96594759	16.36762412	13.22131761
H	11.57979091	16.97802157	14.15814706
H	13.24188789	17.42067904	14.64147934
H	11.87262108	13.35538627	15.16722360
H	12.15897729	14.00079103	13.52713057
H	10.76177349	14.57092495	14.47944709
H	12.82279720	16.49792583	16.99293890
H	11.16467079	15.98628552	16.56521239
H	12.32682099	14.79113252	17.19147244
O	14.01492467	14.79797573	15.12514153
O	15.03651013	16.66242392	16.08288326
N	16.22182658	14.76522596	15.54613569

Product 3a

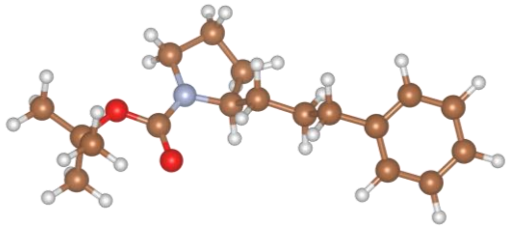

48
Lattice="15.0 0.0 0.0 0.0 15.0 0.0 0.0 0.0 15.0" Properties=species:S:1:pos:R:3 pbc="T T T"
C 9.26757696 6.67544425 6.01478308
C 9.14329591 7.57876217 7.25138158
C 10.34747364 7.12669194 5.06524891
C 8.08322969 7.08489060 8.23288858
C 10.16096177 8.25554967 4.25367683
C 11.57560709 6.45493269 4.99475956
C 11.17037537 8.70175123 3.40022743
C 12.58906188 6.89505637 4.13981259
C 12.39043715 8.02198827 3.34037751
C 7.84715238 8.04125547 9.40957904
C 8.98305538 8.06798279 10.44663191
C 8.58795884 6.97587490 11.44925952
C 7.06778469 7.12433606 11.55121207
C 5.42183282 7.87160039 9.80971456
C 3.07981106 7.60230482 10.53812742
C 2.66067925 6.77110523 9.32636082
C 2.48690071 7.02137411 11.81937575
C 2.70227584 9.07511830 10.38884461
H 9.47207751 5.64175218 6.33942604
H 8.29534056 6.65533751 5.49324185
H 8.89398637 8.60351622 6.92482442
H 10.12899520 7.64577985 7.74214089
H 7.12289872 6.96762443 7.70604014
H 8.36101892 6.08817279 8.61948788
H 9.20661035 8.78857076 4.28974450
H 11.73497500 5.56899726 5.61500162
H 11.00352051 9.58127439 2.77478010
H 13.53593352 6.35274142 4.09555972
H 13.17913356 8.36692393 2.66938895
H 7.64379623 9.04448926 9.00077462
H 9.00858822 9.04855907 10.94705164
H 9.96845784 7.90100455 9.99141633
H 8.84490373 5.98199487 11.05188847
H 9.08321621 7.08302200 12.42385089
H 6.55207905 6.17998302 11.77507818
H 6.78238782 7.85121918 12.33223200
H 2.99360218 5.72935939 9.44512904
H 1.56332674 6.77128136 9.24670994
H 3.08423760 7.17966020 8.40190888
H 2.82669904 7.59050846 12.69626677
H 2.78844091 5.97161114 11.94502294
H 1.38933095 7.06669360 11.77719891
H 3.13856396 9.50221360 9.47856188
H 1.60713437 9.16906178 10.33785582
H 3.05381956 9.64790761 11.25963807
O 4.53145477 7.47187257 10.77115238
O 5.13533446 8.38026702 8.72286201
N 6.69462862 7.62454033 10.22480071

Catalyst V; Ni (0)

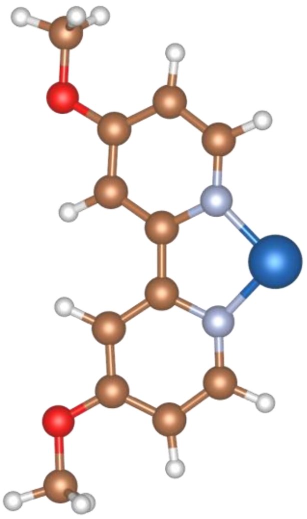

29
Lattice="30.0 0.0 0.0 0.0 30.0 0.0 0.0 0.0 30.0" Properties=species:S:1:pos:R:3 pbc="T T T"
C 14.56833603 15.31757482 14.83516529
C 16.00860763 15.39789268 14.98698950
C 13.92458799 15.18363782 13.59297753
C 16.91566277 15.10567719 13.95396933
C 12.53714923 15.11343770 13.52146134
C 18.28654158 15.19329601 14.17309105
C 11.78225312 15.19834169 14.71107154
C 18.75622589 15.56302556 15.45163572
C 12.47453037 15.34845241 15.90827823
C 17.81137931 15.82207867 16.43887460
C 10.57440522 14.95525174 12.16819749
C 20.50398368 14.91483265 13.33182156
H 14.49745866 15.16426378 12.66634137
H 16.56292814 14.77420040 12.97776192
H 10.69626602 15.13893434 14.72547591
H 19.81378424 15.66348322 15.68447114
H 11.92709955 15.41629084 16.85038537
H 18.13424933 16.11092218 17.44105786
H 10.35638186 14.89059397 11.09582640
H 10.12414190 15.87404022 12.57991299
H 10.15010837 14.07621810 12.68198625
H 20.95833916 14.61419412 12.38093376
H 20.79974818 14.20418941 14.12161812
H 20.85063148 15.92687794 13.60024497
O 11.99722323 14.98256513 12.27215364
O 19.09444351 14.88839098 13.11306685
N 13.81659600 15.39019160 16.00850254
N 16.47959369 15.77085383 16.24676228
Ni 15.00162366 15.80791124 17.39839911

Intermediate VI; Ni (I)

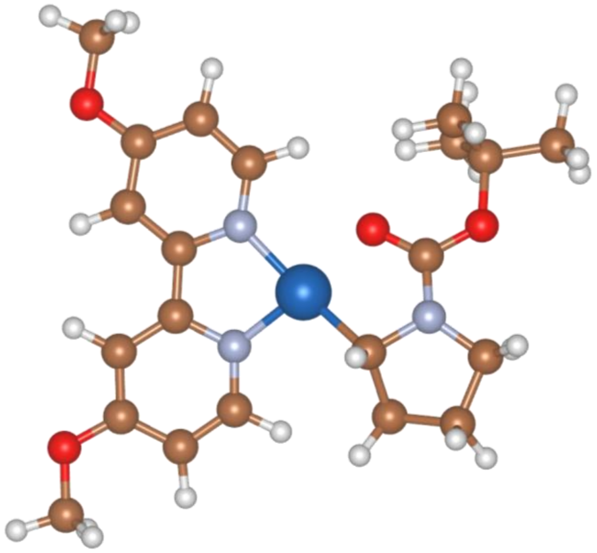

57
Lattice="30.0 0.0 0.0 0.0 30.0 0.0 0.0 0.0 30.0" Properties=species:S:1:pos:R:3 pbc="T T T"

C	14.34224396	14.85966597	11.74368217
C	15.76493232	14.86582253	11.82163074
C	13.59214007	14.85226486	10.54906830
C	16.65376990	14.69695065	10.73959097
C	12.21550165	14.71529875	10.59075072
C	18.02219360	14.73043356	10.96114411
C	11.57661467	14.60774217	11.85115113
C	18.50735514	14.90624878	12.28011503
C	12.36072003	14.70940862	12.99102738
C	17.57391958	15.03446195	13.30241218
C	10.13459562	14.57447026	9.42475333
C	20.23705452	14.56783984	10.07551267
C	13.57886581	15.81684623	15.82985773
C	12.21425174	15.28677199	16.27878084
C	12.06330507	15.82785759	17.71647170
C	13.47972511	15.74651513	18.30885365
C	15.61624675	15.45931492	17.04746976
C	17.70907967	15.11250887	18.30942407
C	18.48504453	16.24331389	17.63919368
C	17.95241325	15.10479305	19.81560781
C	18.02109032	13.74478612	17.70643308
H	14.08858566	14.95963726	9.58454683
H	16.28072827	14.51471243	9.73209515
H	10.50573020	14.45181284	11.95749357
H	19.56821709	14.94805012	12.51490488
H	11.89391820	14.66458354	13.97104576
H	17.89783566	15.15348051	14.33752849
H	9.80738132	14.60767243	8.37873473
H	9.66700433	15.40368948	9.98228534
H	9.82798337	13.61431185	9.87320348
H	20.68541794	14.41013846	9.08746823
H	20.54494171	13.75293556	10.75231000
H	20.58264821	15.53223345	10.48438504
H	13.46811383	16.89063106	15.55972352
H	12.20066457	14.18290350	16.28499641
H	11.39300226	15.63721154	15.63984110
H	11.73448949	16.87770996	17.67946079
H	11.33441209	15.26886556	18.32032993
H	13.71645241	16.58926520	18.97667184
H	13.64486157	14.81383417	18.87274876
H	18.20990889	17.21068118	18.08380916

H	19.56282406	16.08913336	17.79469027
H	18.28696160	16.27845798	16.56139269
H	17.36445158	14.31215737	20.29948368
H	17.67219930	16.07037041	20.25883748
H	19.01699989	14.92230806	20.01794114
H	17.85892396	13.73304699	16.62297084
H	19.07307773	13.49595222	17.91079774
H	17.39269702	12.96995852	18.16835060
O	11.55388116	14.70486317	9.39154133
O	18.82690995	14.56560645	9.86537501
O	16.25424414	15.40055487	18.22943702
O	16.17063789	15.24790678	15.92246547
N	13.70076864	14.84259639	12.99021735
N	16.24596326	15.04110668	13.11141923
N	14.32361602	15.75627658	17.11105778
Ni	14.84357386	15.20694945	14.45204272

Intermediate VII; Ni (II)

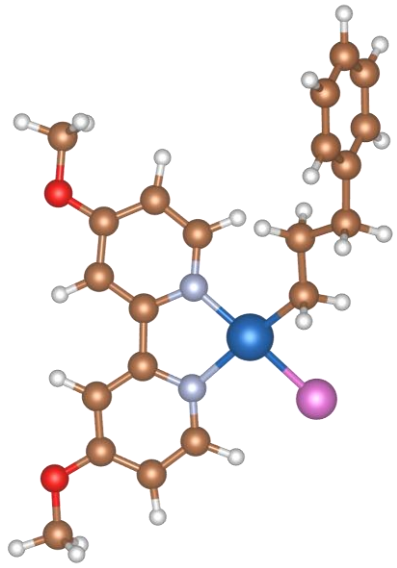

50

Lattice="30.0 0.0 0.0 0.0 30.0 0.0 0.0 0.0 30.0" Properties=species:S:1:pos:R:3 pbc="T T T"

C	13.06904629	15.41521639	12.63958947
C	14.48012667	15.22699290	12.96903517
C	12.60239620	15.86994392	11.41086312
C	15.50485850	15.35210012	12.03969617
C	11.22295919	15.94423669	11.18805658
C	16.81797999	15.03347784	12.39847395
C	10.35888789	15.57287512	12.22834093
C	17.04096710	14.54560448	13.69309338
C	10.90743260	15.14183475	13.43070463
C	15.96924154	14.46535323	14.57208407
C	9.42695113	16.43206282	9.69604148
C	19.11965034	14.87780160	11.79513749
C	15.58529985	15.45705536	19.01422638
C	15.41368016	15.05759887	17.52856193
C	17.03097490	15.51805516	19.43080375
C	13.94650257	14.89843653	17.17508020
C	17.74800688	14.33911680	19.68919354
C	17.70980725	16.74024962	19.53283857
C	19.09763118	14.37921212	20.04110111
C	19.06016418	16.78740078	19.88769080
C	19.75980240	15.60602862	20.14267253
H	13.28298261	16.16383802	10.61270275
H	15.29813183	15.67156462	11.01912480
H	9.27596202	15.60828004	12.12585353
H	18.02371514	14.23054492	14.03815510
H	10.28148145	14.83906790	14.27427817
H	16.12667266	14.09136201	15.57731823
H	9.33556152	16.77995231	8.66144093
H	8.92438419	17.14223998	10.37201300
H	8.96555042	15.43619992	9.79427126
H	19.71691737	15.10154880	10.90457051
H	19.22050055	13.81032534	12.04970461
H	19.46632035	15.49540134	12.63937855
H	15.10005448	16.43135997	19.18225859
H	15.04654203	14.72177037	19.63411338
H	15.96518552	14.10889755	17.39758727
H	15.92504641	15.81478048	16.90947217
H	13.50625082	14.11786610	17.81137015
H	13.40728401	15.83774354	17.40545651
H	17.23324822	13.37672395	19.62051121
H	17.16521571	17.66839887	19.33957441

H	19.63403721	13.44973833	20.24466183
H	19.56647865	17.75148507	19.97112384
H	20.81365585	15.64041314	20.42570118
O	10.83243466	16.37563683	9.96125301
O	17.76916462	15.19919961	11.44462904
N	12.23188308	15.05068955	13.65424071
N	14.70406005	14.83313693	14.27102455
Br	11.21833578	14.02721655	16.45607110
Ni	13.16482044	14.69173172	15.37931248

Intermediate VIII; Ni (III)

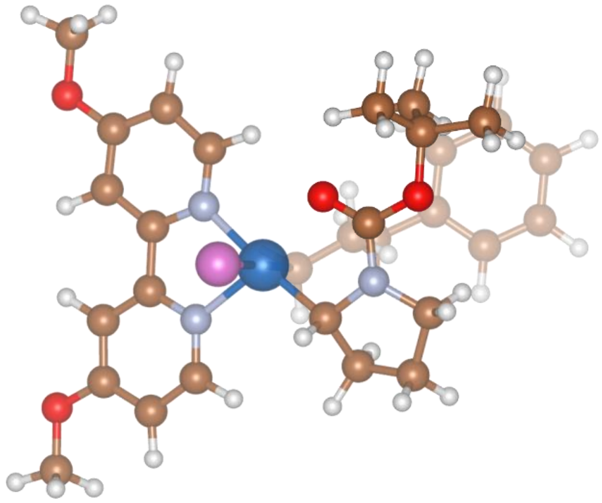

78

Lattice="30.0 0.0 0.0 0.0 30.0 0.0 0.0 0.0 30.0" Properties=species:S:1:pos:R:3 pbc="T T T"

C	13.75794749	16.33689465	12.25877894
C	15.21589975	16.18645670	12.42454125
C	13.11050247	16.29956367	11.02685643
C	16.11189525	16.13090994	11.36139138
C	11.72570120	16.49892690	10.97491456
C	17.48294394	16.03447022	11.62531627
C	11.03319417	16.73491659	12.17121108
C	17.91040044	15.99628093	12.95998884
C	11.76066380	16.73779728	13.35840685
C	16.94879811	16.05992618	13.96317167
C	9.74223982	16.64995851	9.65681268
C	19.71279662	15.94054821	10.78250094
C	12.99315404	16.58436956	16.63286192
C	11.74332153	15.79125109	16.99979169
C	11.53585087	16.15606191	18.48252041
C	12.96244453	16.20955946	19.05492706
C	15.11738758	16.31532433	17.81555695
C	17.12535183	15.96656027	19.20296176
C	17.88581352	17.18374672	18.68021531
C	17.27178972	15.84453406	20.71732624
C	17.55321066	14.66705503	18.52163417
C	14.12732254	12.08082440	16.16772575
C	14.42564766	13.59425636	16.27112670
C	14.60256796	11.34514453	17.39159985
C	13.85620664	14.39734104	15.12424274
C	15.95971386	11.03159609	17.56221158
C	13.71482294	11.02455440	18.42853828
C	16.41511719	10.41731211	18.72971816
C	14.16376929	10.40749344	19.59818704
C	15.51768582	10.10254952	19.75389821
H	13.65082990	16.11823561	10.09841515
H	15.77726464	16.18580403	10.32626970
H	9.96018182	16.91480699	12.19796164
H	18.96179597	15.93231561	13.23288484
H	11.26473676	16.91758754	14.31407077
H	17.22396414	16.05752471	15.01833482
H	9.49533533	16.56069996	8.59322740
H	9.46359317	17.65288117	10.01870794
H	9.19523816	15.88329914	10.22944195
H	20.18097262	15.93335810	9.79210599
H	19.98776417	15.02312722	11.32811053
H	20.05274157	16.82406249	11.34667499
H	12.72753577	17.64511528	16.47597952

H	11.90247576	14.70502975	16.90270646
H	10.87554913	16.05918052	16.38025863
H	11.06336277	17.14702370	18.55882568
H	10.90539943	15.43805959	19.02632399
H	13.09083204	16.99758890	19.81235607
H	13.26405983	15.25267782	19.51168401
H	17.53890734	18.09626820	19.18579562
H	18.95897250	17.06093195	18.88939006
H	17.74313431	17.30875554	17.60047598
H	16.69708352	14.98654904	21.09377427
H	16.91052477	16.75561609	21.21442003
H	18.32953315	15.69917800	20.97859843
H	17.48711746	14.74781814	17.43081911
H	18.59421175	14.44179776	18.79772289
H	16.92268591	13.82915618	18.85263843
H	13.04236542	11.93303289	16.03977366
H	14.61399953	11.67878004	15.26332600
H	15.51645737	13.72909280	16.33329315
H	14.02327429	13.94701692	17.23504825
H	14.28502034	14.05926737	14.16526808
H	12.76286494	14.28693595	15.05981071
H	16.66753631	11.27568069	16.76532550
H	12.65358638	11.26068088	18.31100566
H	17.47513514	10.17891737	18.84049697
H	13.45281671	10.16018661	20.38955851
H	15.87121170	9.61768570	20.66573484
O	11.15612428	16.44587460	9.74300547
O	18.30292385	15.99535468	10.54272397
O	15.67053239	16.18047180	19.04512269
O	15.75890403	16.27648416	16.74002511
N	13.08002274	16.53893652	13.41678960
N	15.63542049	16.14842834	13.71552510
N	13.78544818	16.47433999	17.86676390
Br	14.66569732	18.83040490	15.04072411
Ni	14.24112599	16.34949925	15.12139423

Intermediate IX; Ni (I)

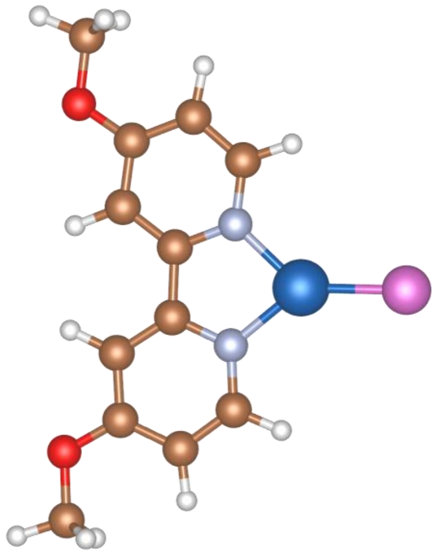

30

Lattice="30.0 0.0 0.0 0.0 30.0 0.0 0.0 0.0 30.0" Properties=species:S:1:pos:R:3 pbc="T T T"

C	14.67972211	14.29586484	14.23149131
C	16.13718665	14.32966425	14.39061558
C	14.01534073	14.12616370	13.02276006
C	17.05481418	14.14398967	13.36332819
C	12.61541896	14.11339817	13.00131983
C	18.42511989	14.19175444	13.64649079
C	11.92352794	14.26828993	14.21408198
C	18.83008784	14.43563564	14.96949038
C	12.65933119	14.43643225	15.38068733
C	17.85209008	14.61001945	15.94096325
C	10.60156882	13.94518433	11.73171630
C	20.67205467	14.00857565	12.86290757
H	14.55917648	14.00781034	12.08578220
H	16.73309035	13.95324526	12.33968592
H	10.83675853	14.26201535	14.26800287
H	19.87859286	14.49053420	15.25536201
H	12.16199242	14.56256581	16.34427807
H	18.12122024	14.79970349	16.98193036
H	10.34526965	13.82727577	10.67316152
H	10.18641136	14.89376855	12.10902802
H	10.18670462	13.10306368	12.30905643
H	21.15722962	13.81821750	11.89924486
H	20.94922252	13.21814968	13.57932023
H	20.99238552	14.98932852	13.25066424
O	12.03117392	13.95130691	11.78436225
O	19.26634468	13.99221598	12.59715966
N	14.00709831	14.45752514	15.41626593
N	16.52953304	14.55828501	15.68459592
Br	14.90468702	15.07653070	19.16124305
Ni	15.10811648	14.72967639	16.96344635
